# Synthesis and properties of l-*lyxo*-thioBsNA-pyrimidine nucleoside: a sulfur-containing analogue of boat-shaped pyranosyl nucleic acid

**DOI:** 10.1039/d6cb00034g

**Published:** 2026-03-25

**Authors:** Miyu Ito, Suguru Matsumoto, Natsuki Yokota, Hidekazu Hiroaki, Tetsuya Kodama

**Affiliations:** a Graduate School of Pharmaceutical Sciences, Nagoya University, Furo-cho, Chikusa-ku Nagoya Aichi 464-8601 Japan kodama.tetsuya.c6@f.mail.nagoya-u.ac.jp; b Center for One Medicine Innovative Translational Research (COMIT), Nagoya University, Furo-cho, Chikusa-ku Nagoya Aichi 464-8601 Japan

## Abstract

l-*lyxo*-thioBsNA, a boat-shaped nucleic acid (BsNA) analogue designed to enhance hydrophobicity – a property strongly influencing its physical characteristics – was synthesized. Oligonucleotides containing l-*lyxo*-thioBsNA were efficiently synthesized using the phosphoramidite method with levulinyl chemistry, and their hydrophobicity was higher than that achieved using PS-modification, as revealed by analysis using reverse-phase HPLC. The double helix with the complementary RNA strand is highly stable, and the introduction of sulfur atoms with large atomic radii did not adversely affect thermal stability. These characteristics demonstrate that l-*lyxo*-thioBsNA is an ideal nucleic acid modifier for RNA-targeting technologies such as antisense methods, offering a new option for use in situations requiring hydrophobic nucleic acids.

## Abstract 2

l-*lyxo*-thioBsNA, a boat-shaped nucleic acid (BsNA) analogue designed to enhance hydrophobicity – a property strongly influencing its physical characteristics – was synthesized.

## Graphical abstract

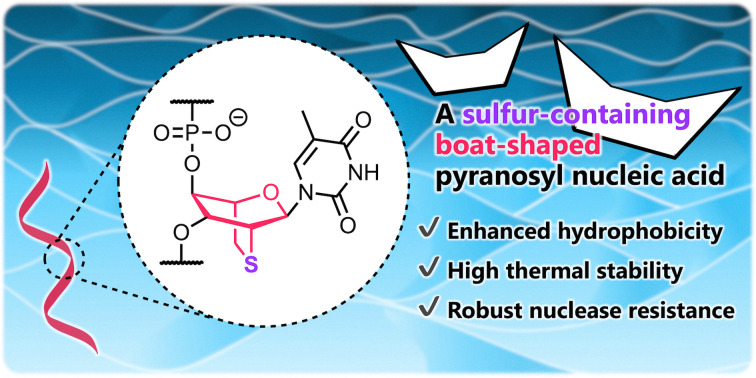

## Introduction

Oligonucleotide analogues possess immense potential not only as therapeutic agents but also as recognition units for nanotechnology, driving accelerated research into nucleic acid mimetics over the past decade.^1^ Research on 2'-modified RNA and conformationally restricted nucleic acids, which mimic natural RNA structures, has been particularly remarkable, and some of the analogues are now widely recognized as artificial nucleic acids capable of forming stable duplexes and readily available for anyone to use in their research.^1^

Artificial nucleic acids exhibit properties useful for chemical biology, for example furanosyl nucleic acids based on furanoses such as ribose. In particular, since it was reported that the locked nucleic acid (LNA)/2′-O,4′-C-methylene bridged acid (2′,4′-BNA),^2^ an RNA mimic that restricts conformational changes during duplex formation, exhibit extremely high duplex formation ability with complementary nucleic acids, numerous studies^3–5^ have been conducted aiming to create LNA analogues that exhibit functions unattainable with the original LNA/BNA modification. Similarly, tricyclo-DNA, which can be regarded as a DNA mimic, has been reported as a unique artificial nucleic acid that exhibits high duplex-forming ability and strong resistance to degradation, and is therefore considered a promising candidate for therapeutic applications.^6^

On the other hand, pyranosyl nucleic acids possessing pyranose sugars useful for these purposes have not been found, with the exception of d-altritol nucleic acid (ANA)^7^ and the deoxy analogue hexitol nucleic acid (HNA)^8^ with a nucleobase at the C2′ position. In this context, it has been reported that pyranosyl nucleic acids bearing a nucleobase at the C1′ position are generally unable to form duplexes with complementary DNA or RNA,^9^ except for one example in which an unstable duplex can be formed.^10^ These findings suggest that a conformationally restricted design strategy—demonstrated to be highly effective for furanosyl nucleic acids—cannot be readily applied to pyranosyl nucleic acids. Furthermore, the observation that constrained altriol nucleic acid (cANA), which was designed to control the conformation of HNA capable of forming stable duplexes, actually decreases duplex stability further highlighted the inherent difficulty of achieving conformational control in pyranosyl nucleic acids.^11^ Meanwhile, we have studied pyranosyl nucleic acids whose conformation is restricted in a boat-shape (BsNAs),^12^ and found that isoBsNA and l-*lyxo*-BsNA form stable duplexes with the complementary RNA under physiological conditions.^13^ A series of BsNAs are the only analogues that have high affinity with RNA in the pyranosyl nucleic acids having nucleobases at the anomeric C1′ position. Therefore, by creating BsNA analogues with various physical properties, it becomes possible to discover characteristics not present in previously developed artificial nucleic acids.

**Fig. 1:**
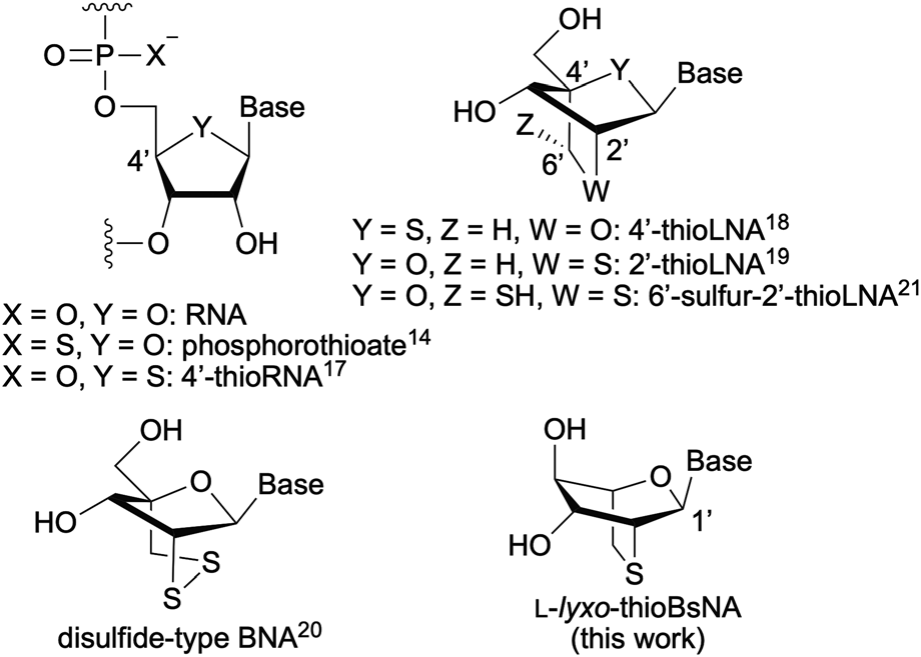
Examples of sulfur-containing artificial nucleic acids.

The most representative example of nucleic acid modification is phosphorothioate (PS) modification of the phosphate backbone, in which one of the oxygen atoms of the phosphate group is replaced by a sulfur atom, which is essential for enhancing *in vivo* stability, such as strong resistance to nucleases and appropriate binding ability to serum proteins (Fig. 1).^14^ However, it is also true that reducing or eliminating PS modification has emerged as a major challenge in oligonucleotide-based medicinal chemistry because PS modification has been implicated in toxicity^15^ and undesirable tissue distribution.^16^Similarly, interesting properties have been reported for artificial nucleic acids bearing a sulfur-modified sugar moiety. For example, 4′-thio nucleic acids^17^ and their LNA analogues^18^ exhibit RNA-like structural properties and high duplex forming ability. 2′-thioLNA^19^ can also show high duplex forming ability, and the analogues possess the redox sensing feature^20^ and post-synthesis modification capabilities^21^ in addition to these properties. In particular, the common features of sulfur-modification are increased hydrophobicity and potential resistance to nucleases, suggesting that the use of sugar-modified sulfur-containing artificial nucleic acids has the potential to avoid phosphorothioate modification.

In this study, we synthesized l-*lyxo*-thioBsNA which is a boat-shaped pyranose nucleic acid analogue containing a sulfur atom and evaluated the hybridization properties and the tolerance to the nuclease of l-*lyxo*-thioBsNA oligonucleotides (Fig. 1).

## Results and discussion

### Synthesis of l-*lyxo*-thioBsNA thymine and 5-methylcytosine nucleosides

**Scheme 1:**
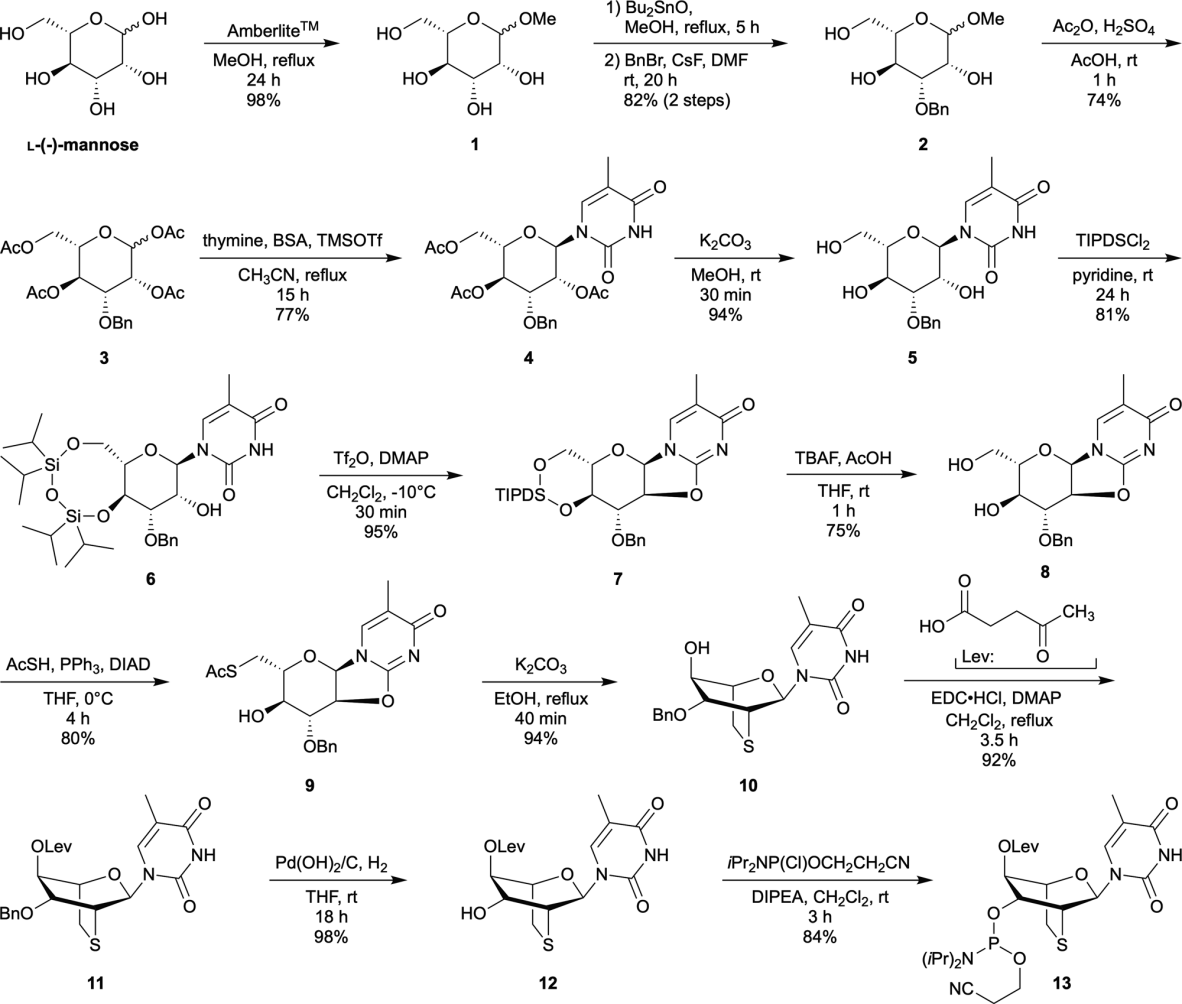
Synthesis of l-*lyxo*-thioBsNA-T phosphoroamidite.

An l-*lyxo*-thioBsNA thymine analogue, l-*lyxo*-thioBsNA-T, was synthesized from l-mannose as shown in Scheme 1. In the synthesis process, all reaction conditions were optimized using methyl α-d-mannoside as the starting material (Scheme S1), and an established synthetic route for d-*lyxo*-thioBsNA was applied. First, methylation of l-mannose was performed under acidic conditions to obtain a 1 : 1 anomeric mixture of methyl l-mannoside (1). Despite compound 1 being a stereoisomeric mixture, selective benzylation at the 3-position *via* stannylene acetalization of 1 proceeded efficiently, yielding compound 2. The conversion from 2 to tetra-acetate 3 was achieved under sulfuric acid acidic conditions in moderate yield. The NMR spectrum of the resulting 3 was consistent with that of the d-isomer. A thymine base was introduced under TMSOTf acidic conditions, leading to the formation of a protected l-mannopyranosylthymine analogue 4. Compound 6 was obtained by deacetylation of 4 followed by TIPDS protection of resulting 5. Compound 6 was subjected to trifluoromethylation conditions to promote cyclization at the C2 and C2′ positions, giving compound 7, which was subsequently converted to 8 by desilylation using TBAF-AcOH. The introduction of sulfur atoms into the compound was achieved by a Mitsunobu reaction with thioacetic acid, yielding 6-thiocyclonucleoside 9. The thioBsNA skeleton of 10 was quantitatively constructed by treating 9 with potassium carbonate in ethanol under reflux conditions. Initially, we considered protecting the hydroxyl group of the obtained 10 with a DMTr group suitable for oligonucleotide synthesis; however, we opted for the levulinyl group instead, since the DMTr group was simultaneously removed under the debenzylation conditions, preventing the synthesis of the tritylated compound (see Scheme S1). Therefore, the hydroxyl group of 10 was esterified with levulinic acid, and the resulting compound 11 was subjected to hydrogenolysis in the presence of palladium–carbon to give compound 12. Finally, phosphitylation at the 3′-hydroxy group of 12 with 2-cyanoethyl *N*,*N*-diisopropylaminochlorophosphoramidite afforded the desired phosphoramidite building block 13.

**Scheme 2:**
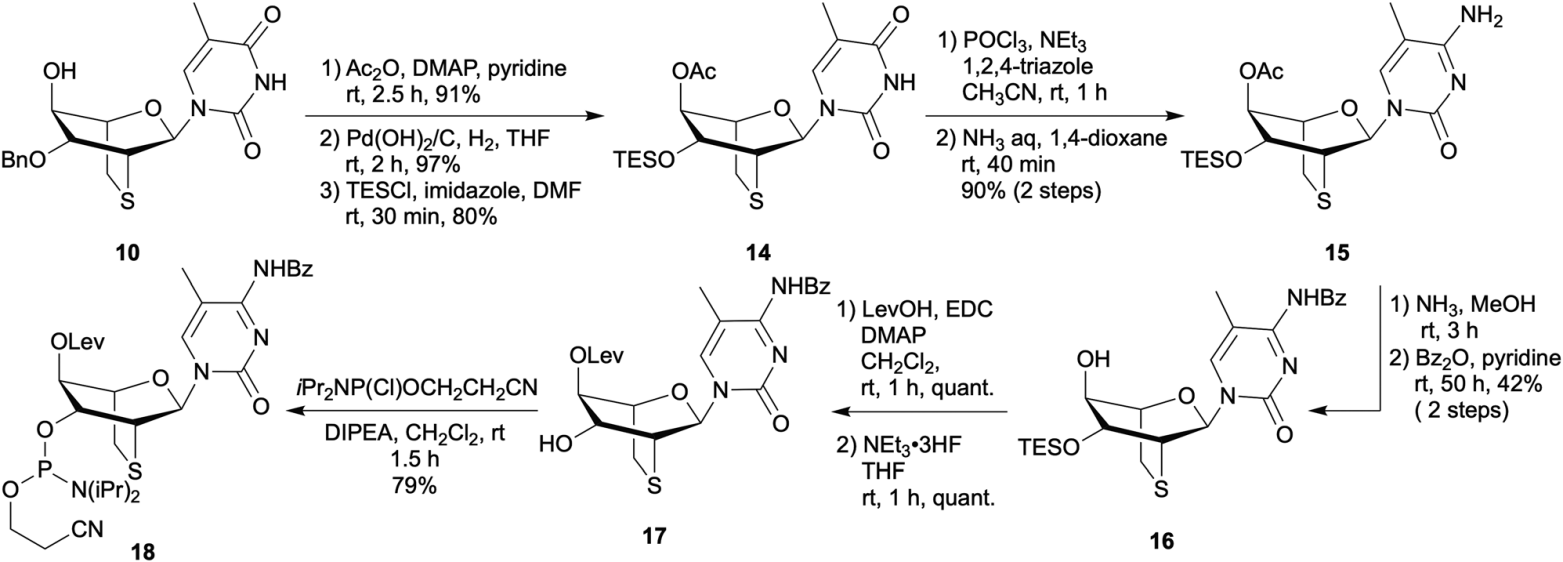
Synthesis of l-*lyxo*-thioBsNA-meC phosphoroamidite.

A 5-methylcytidine analogue of l-*lyxo*-thioBsNA, l-*lyxo*-thioBsNA-mC, was synthesized from intermediate 10 as shown in Scheme 2. Since debenzylation did not proceed after converting thymine to 5-methylcytosine (Scheme S2), the protecting group of 10 was first replaced with the TES group to obtain compound 14. The obtained compound 14 was converted to 5-methylcytosine derivatives 15 in high yield *via* triazolylation at the 4-position, followed by ammonolysis. After deacetylation of 15 with ammonia-methanol solution, the resulting compound was converted to compound 16 by protecting its cytosine base with a benzoyl group. Obtained 16 was then esterified with levulinic acid, followed by desilylation with fluoride ions to yield compound 17. Finally, the phosphoramidite building block 18 was obtained by phosphitylating the 3′-hydroxy group of 17 with 2-cyanoethyl *N*,*N*-diisopropylaminochlorophosphoramidite.

### Synthesis of l-*lyxo*-thioBsNA-containing oligonucleotides

**Fig. 2:**
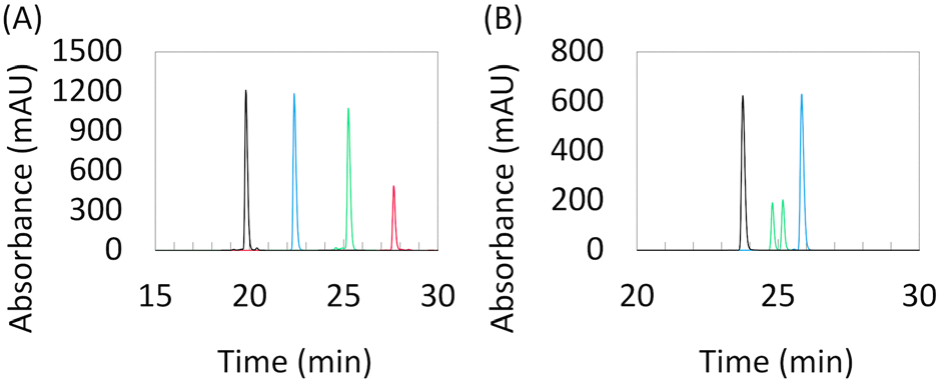
Overlaid reverse-phase HPLC charts of ONs. (A) ON1: black, ON2: blue, ON3: green and ON5: red. (B) ON9: black, ON10: green and ON12: blue.

To evaluate the properties of l-*lyxo*-thioBsNA in oligonucleotides (ONs), phosphoramidites 13 and 19 were incorporated into ONs using an automated DNA synthesizer. Each coupling reaction was accomplished using 5-benzylthio-1*H*-tetrazole as an activator, and the coupling time was prolonged to 12 min for l-*lyxo*-thioBsNA. Hydrazine and trichloroacetic acid were used as deblocking reagents for l-*lyxo*-thioBsNA and natural nucleoside units, respectively. The standard iodine oxidation conditions were applicable to the oxidation of the phosphorus atom, and under these conditions the sulfur atom of l-*lyxo*-thioBsNA was not oxidized to sulfoxide or other higher oxidation states. Synthesized ONs were cleaved from the solid supports and deprotected by treatment with ammonium hydroxide solution. The obtained ONs were purified by reverse-phase HPLC and characterized by MALDI-TOF mass spectrometry. Sequences are shown in Table 1. Comparing the retention times of synthesized l-*lyxo*-thioBsNA-modified oligonucleotides by reverse-phase HPLC, the retention time increases as l-*lyxo*-thioBsNA modification increases, clearly demonstrating the high hydrophobicity of l-*lyxo*-thioBsNA (Fig. 2A).^22^ Furthermore, it was found that l-*lyxo*-thioBsNA-modified oligonucleotides exhibit higher hydrophobicity than PS-oligonucleotides (Fig. 2B). ON10 exhibited two peaks in the HPLC spectrum due to the presence of stereoisomers on the phosphorothioate moiety.

**Table 1:** Sequences of oligonucleotides^a^^b^

		MALDI-TOF-MS	
	Sequences (5′–3′)	Calc.	Found
ON1	GCG TTT TTT GCT	3633.4	3632.7
ON2	GCG TT**T** TTT GCT	3677.5	3677.1
ON3	GCG TT**T** T**T**T GCT	3721.5	3721.3
ON4	GCG TT**T** TT**T** GCT	3721.5	3721.2
ON5	GCG **T**T**T** T**T**T GCT	3765.6	3764.3
ON6	GCG TT**T TT**T GCT	3765.6	3764.2
ON7	GCG TTC TTT GCT	3616.7	3617.6
ON8	GCG TT**C** TTT GCT	3676.5	3675.9
ON9	TTT TTT TTT T	2980.0	2979.6
ON10	TTT TTT TTT^T	2996.4	2994.3
ON11	TTT TTT TTT **T**	3024.1	2023.1
ON12	TTT TTT TT**T** T	3024.1	3022.4

a Capital letters and bold letters indicate DNA and l-*lyxo*-thioBsNA, respectively.

b ^ = phosphorothioate.

### Effect of l-*lyxo*-thioBsNA on the thermal stability of the oligonucleotide duplex

**Fig. 3:**
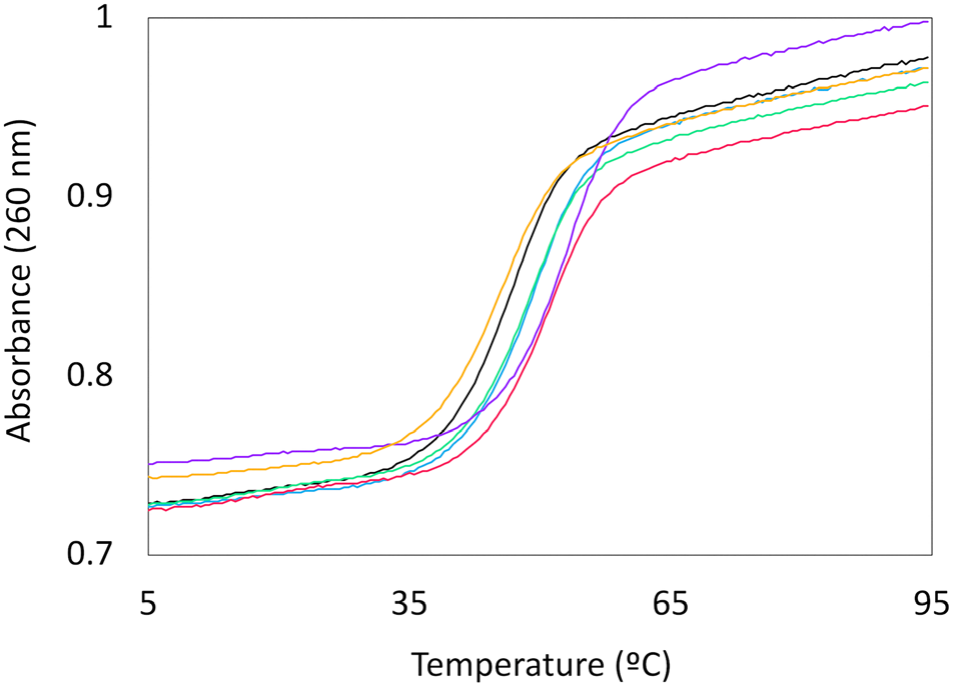
Representative UV melting curves of the l-*lyxo*-thioBsNA-RNA duplex, ON1: black, ON2: blue, ON3: green, ON4: violet, ON5: pink, and ON6: orange and the complementary RNA: 5′-r(AGC AAA AAA CGC)-3′. Conditions: 10 mM sodium phosphate (pH 7.2), 100 mM NaCl, and 4.0 µM of each oligonucleotide.

To evaluate the properties of l-*lyxo*-thioBsNA in oligonucleotides, the thermal stability of l-*lyxo*-thioBsNA-modified ONs with complementary single-stranded DNA and RNA was first evaluated using UV melting experiments. The UV melting curves are shown in Fig. 3 for RNA and in Fig. S1 for DNA and their 50% thermal denaturation temperatures (*T*_m_ values) are summarized in Table 2. A single l-*lyxo*-thioBsNA-T modification in oligonucleotides resulted in a 3.1 °C decrease for the DNA complement and in a 2.4 °C increase for the RNA complement in the *T*_m_ value. The influence of multiple l-*lyxo*-thioBsNA-T modification varied depending on their modification pattern. When the modifiers were spaced apart, a strong stabilizing effect on the duplex with complementary RNA was observed. However, this effect diminished as the modifiers were placed closer together and was completely lost when three modifiers were positioned consecutively. Regarding duplex stability with complementary DNA, l-*lyxo*-thioBsNA-T modification generally caused destabilization, and furthermore, it was found that the extent of this destabilization depended on the modification pattern, just as it did for the duplex stability with RNA. Also, the effect of l-*lyxo*-thioBsNA-meC on *T*_m_ was similar to that of l-*lyxo*-thioBsNA-T, selectively stabilizing the duplex with RNA complement. Eschenmoser and colleagues reported that when homopyrimidine sequences were fully modified with l-α-threofuranosyl nucleic acid (TNA)^23^ or l-α-lyxopyranosyl oligonucleotides,^10^ the *T*_m_ values decreased markedly. Their observations are consistent with the findings of our study. Considering that TNA is a furanosyl nucleic acid with a similar chemical structure to that of l-*lyxo*-thioBsNA, it is possible that BsNA has comparable properties to TNA. They also reported that the full modification of homopurine sequences or mixed sequences with TNA leads to an increase in *T*_m_ values. Therefore, synthesizing l-*lyxo*-(thio)BsNA-purine analogues and elucidating their properties may enable us to uncover even more intriguing characteristics of l-*lyxo*-(thio)BsNA series in the future.

**Table 2:** *T*_m_ values of duplexes containing l-*lyxo*-thioBsNA

	*T*_m_ (Δ*T*_m_/mod.), °C		RNA selectivity
	ssDNA^a^	ssRNA^a^	*T*_m_ (RNA)–*T*_m_ (DNA)
ON1	50.7	46.1	–4.6
ON2	47.5 (–3.1)	48.4 (+2.4)	+0.9
ON3	41.2 (–4.7)	48.2 (+1.1)	+7.1
ON4	49.8 (–0.4)	52.4 (+3.2)	+2.6
ON5	34.6 (–5.3)	50.4 (+1.5)	+15.8
ON6	27.3 (–7.8)	45.0 (–0.4)	+17.6
ON7	52.9	52.6	–0.3
ON8	53.2 (+0.3)	57.2 (+4.5)	+4.0

a For ON1 to ON6, 5′-d/r(AGC AAA AAA CGC)-3′, for ON7 and ON8, 5′-d/r(AGC AAA GAA CGC)-3′. Conditions: 10 mM sodium phosphate buffer (pH = 7.2), 100 mM NaCl solution, and 4 µM each oligonucleotide. The melting profiles were recorded at 260 nm from 5 to 95 °C at a scan rate of 0.5 °C min^−1^. Each number represents the average of *T*_m_ values from three independent experiments.

The base-recognition ability of l-*lyxo*-thioBsNA was then examined using single-mismatch DNA and RNA strands (Table 3 and Fig. S2 to S9). As shown in Table 3, any mismatched l-*lyxo*-thioBsNA-T base pairs in duplex with DNA or RNA strands were shown to cause significant destabilization of the duplex. This result suggests that l-*lyxo*-thioBsNA forms typical Watson–Crick base pairs appropriately.

**Table 3:** Mismatch recognition ability

	*T*_m_ (Δ*T*_m_ = Δ*T*_m_ [mismatch] − Δ*T*_m_ [match]), °C			
	X = A	X = G	X = C	X = T
ON1/DNA	50.7	39.0 (–11.4)	35.6 (–15.1)	36.5 (–14.1)
ON2/DNA	47.5	34.0 (–13.6)	30.9 (–16.6)	34.0 (–13.0)
ON1/RNA	46.1	43.1 (–3.0)	29.9 (–16.2)	32.6 (–13.5)
ON2/RNA	48.4	40.3 (–8.1)	33.0 (–15.4)	34.0 (–14.5)

### Resistance to exonucleolytic degradation of l-*lyxo*-thioBsNA

**Fig. 4:**
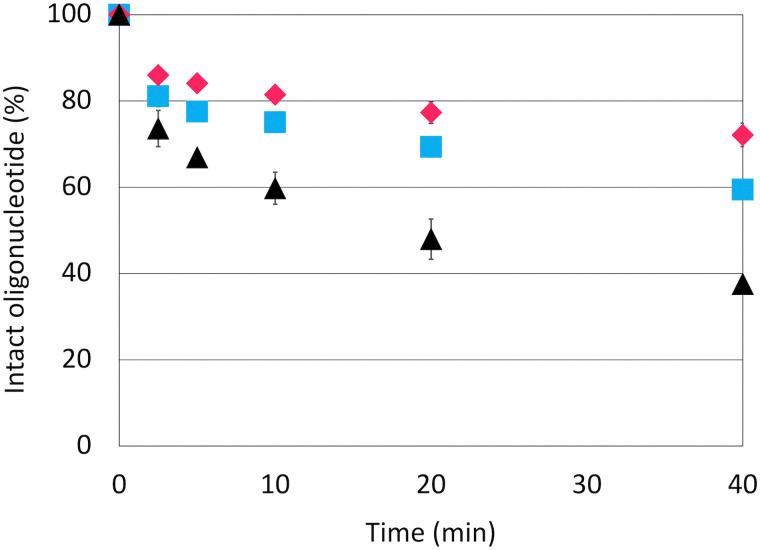
Stability of ON10–ON12 against SVPDE. ON10: black triangles, ON11: pink diamonds, and ON12: blue squares. Conditions: 50 mM Tris–HCl (pH 8.0), 10 mM MgCl_2_, 4.0 µM of each oligonucleotide, and 25 mU PDE at 37 °C. This experiment was conducted in triplicate (*n* = 3 per group). Data are presented as mean ± SD.

Next, we compared the stability of the l-*lyxo*-thioBsNA moiety in a single-stranded thymidine decamer against nucleases with that achieved using phosphorothioate modification. ON10, ON11 and ON12 were treated with snake venom phosphodiesterase I (SVPDE), 5′-exonuclease, and samples collected at each time point were analysed by reverse-phase HPLC. Fig. 4 shows the results of plotting the ratio of the UV area for full-length ONs against time in HPLC (Fig. S10). As can be seen in the results, under conditions where 60% of PS modifications degraded within 40 minutes, only about 30–40% of l-*lyxo*-thioBsNA degraded, indicating that l-*lyxo*-thioBsNA exhibited higher resistance to the nuclease than that observed in PS modification.^24^ Among the many artificial nucleic acids that exhibit degradation resistance at the 3′-terminal side, it was interesting that this modifier showed degradation resistance at both the 3′- and 5′-sides. This result indicates that high resistance to enzymatic degradation can be achieved even without sequential modification, a feature that sufficiently compensates for the potential decrease in duplex stability that l-*lyxo*-thioBsNA may exhibit when sequentially modified, making it suitable for drug discovery applications.

## Conclusions

We successfully synthesized thymidine and 5-methylcytidine analogs of l-*lyxo*-thioBsNA, a sulfur-containing boat-shaped nucleic acid that we designed as a highly hydrophobic artificial nucleic acid. Upon incorporation of l-*lyxo*-thioBsNA into oligodeoxynucleotides, the resulting duplex with its complementary RNA was thermally stabilized, whereas the duplex formed with its complementary DNA was markedly destabilized. This result indicates that the large atomic radius of the sulfur atom did not exert a significant influence on duplex stability. In contrast, it was found by reverse-phase HPLC that the hydrophobicity of ODNs bearing l-*lyxo*-thioBsNA was as high as that of PS-modified ODN, confirming that the sulfur atom had a significant impact on hydrophobicity as intended in the design. Furthermore, l-*lyxo*-thioBsNA exhibited high resistance to degradation by the exonuclease homolog known to be abundant in serum, and hydrolysis at both its 3' and 5' sides required a longer time than that observed for the hydrolysis of the PS-modified moiety. The various properties elucidated in this study demonstrate that L-DNA is an ideal nucleic-acid material for RNA-targeting technologies—particularly those intended for use in biological environments—such as RNA detection platforms and antisense-based therapeutic approaches. Given that its pronounced hydrophobicity is expected to influence the *in vivo* distribution and pharmacokinetic behavior of nucleic acids, we are synthesizing various nucleobase analogs to elucidate their fundamental properties in greater detail, aiming to apply this research to drug discovery.

## Experimental

### Synthesis of l-*lyxo*-thioBsNA phosphoramidites

#### General

All moisture-sensitive reactions were performed under an argon atmosphere in well-dried glassware. Anhydrous acetonitrile (MeCN), dichloromethane (CH_2_Cl_2_), and tetrahydrofuran (THF) were used as received. The other anhydrous solvents were purified as follows: ethanol by drying over activated molecular sieves (3 Å); methanol by distillation; and pyridine and DMF by distillation from KOH and CaH_2_, respectively. All purified solvents were stored over activated molecular sieves (3 Å for methanol, and 4 Å for pyridine and DMF) under an argon atmosphere until use. ^1^H NMR (400 MHz), ^13^C NMR (101 MHz) and ^31^P NMR (162 MHz) spectra were recorded on a JEOL JNM-ECS400 or a JNM-ECZ400 spectrometer. Chemical shifts are reported in parts per million referenced to internal tetramethylsilane (0.00 ppm), residual CHCl_3_ (7.26 ppm), CH_3_OH (3.31 ppm), or DMSO (2.50 ppm) for ^1^H NMR, and CHCl_3_ (77.06 ppm), CH_3_OH (49.03 ppm) or DMSO (39.53 ppm) for ^13^C NMR. H_3_PO_4_ (0.00 ppm) was used as an external standard for ^31^P NMR. High-resolution mass spectra (HRMS) were obtained on a JEOL JMS-T100LP AccuTOF LC-plus spectrometer equipped with an electrospray ionization (ESI) source in positive ion mode. For column chromatography, the Fuji Silysia PSQ-60B silica gel was used. The progress of the reactions was monitored by analytical thin-layer chromatography on pre-coated glass plates (silica gel 60 F254, Merck).

##### Methyl l-mannopyranoside 1

To a solution of l-(-)-mannopyranose (12.61 g, 70.0 mmol) in dry methanol (140 mL) was added Amberlite IRC120 H-form (25 g) at room temperature under an argon atmosphere. After refluxing for 24 h, the reaction mixture was filtered and concentrated under reduced pressure to obtain 1 (13.36 g, 68.8 mmol, 98%) as a pale yellow solid. ^1^H-NMR (400 MHz, DMSO-D6) *δ* 4.71 (d, *J* = 1.4 Hz, 1H), 4.69 (d, *J* = 2.8 Hz, 1H), 4.55 (d, *J* = 6.0 Hz, 1H), 4.48 (d, *J* = 1.4 Hz, 1H), 4.45 (t, *J* = 6.0 Hz, 1H), 3.65 (qd, *J* = 6.0, 2.3 Hz, 1H), 3.59–3.57 (m, 1H), 3.46–3.35 (m, 3H), 3.24 (s, 3H); ^13^C-NMR (101 MHz, DMSO-D6) *δ* 101.0, 73.9, 71.0, 70.2, 67.0, 61.3, 53.9; HRMS (ESI) *m*/*z*: [2M + Na]^+^ calcd for C_14_H_28_NaO_12_^+^ 411.1478; found 411.1461.

##### Methyl 3-*O*-benzyl-l-mannopyranoside 2

To a solution of 1 (12.68 g, 65.3 mmol) in dry methanol (130 mL) was added dibutyltin oxide (19.50 g, 78.3 mmol) at room temperature under an argon atmosphere. After refluxing for 5 h, the reaction mixture was concentrated under reduced pressure. To the residue in dry DMF (130 mL) were added cesium fluoride (11.95 g, 78.7 mmol) and benzyl bromide (11.6 mL, 97.7 mmol) at room temperature under an argon atmosphere. After stirring at room temperature for 20 h, the reaction mixture was filtered and concentrated under reduced pressure. The crude product was purified by flash silica gel column chromatography (CHCl_3_, then CHCl_3_/MeOH = 50 : 1 to 15 : 1) to obtain 2 (15.17 g, 53.4 mmol, 82%) as a brown oil. ^1^H-NMR (400 MHz, CDCL_3_) *δ* 7.38–7.30 (m, 5H), 4.76 (d, *J* = 1.4 Hz, 1H), 4.69 (d, *J* = 11.4 Hz, 1H), 4.58 (d, *J* = 11.5 Hz, 1H), 3.98–3.94 (m, 2H), 3.80 (dd, *J* = 6.4, 3.7 Hz, 2H), 3.67 (dd, *J* = 9.2, 3.2 Hz, 1H), 3.56 (dt, *J* = 9.6, 3.7 Hz, 1H), 3.35 (s, 3H), 3.02 (d, *J* = 2.8 Hz, 1H), 2.86 (d, *J* = 3.2 Hz, 1H), 2.69 (t, *J* = 6.7 Hz, 1H); ^13^C-NMR (101 MHz, CDCL_3_) *δ* 137.6, 128.7, 128.3, 128.1, 100.6, 79.7, 71.9, 71.7, 67.7, 66.2, 62.1, 55.0; HRMS (ESI) *m*/*z*: [M + Na]^+^ calcd for C_14_H_20_NaO_6_^+^ 307.1158; found 307.1131.

##### 1,2,4,6-Tetra-*O*-acetyl-3-*O*-benzyl-l-mannopyranoside 3

To a solution of 2 (11.44 g, 40.2 mmol) in acetic acid (28.6 mL), acetic anhydride (71 mL, 752 mmol) and concentrated sulfuric acid (1.07 mL, 20.0 mmol) were added dropwise at room temperature under an argon atmosphere, and the reaction mixture was stirred at room temperature for 1 h. After the addition of water, the resulting mixture was extracted with AcOEt. The organic layer was washed with brine, dried over Na_2_SO_4_, and concentrated under reduced pressure. The crude product was purified by flash silica gel column chromatography (*n*-hexane, then *n*-hexane/AcOEt = 1 : 1) to obtain 3 (13.02 g, 29.7 mmol, 74%) as a pale yellow oil. ^1^H-NMR (400 MHz, CDCl_3_) *δ* 7.37–7.26 (m, 5H + CHCl_3_), 6.10 (d, *J* = 1.8 Hz, 1H), 5.36 (t, *J* = 2.8 Hz, 1H), 5.29 (t, *J* = 9.9 Hz, 1H), 4.68 (d, *J* = 12.4 Hz, 1H), 4.45 (d, *J* = 12.4 Hz, 1H), 4.23 (dd, *J* = 12.4, 5.0 Hz, 1H), 4.09 (dd, *J* = 12.4, 2.3 Hz, 1H), 3.94 (ddd, *J* = 10.1, 5.0, 2.3 Hz, 1H), 3.86 (dd, *J* = 9.9, 3.4 Hz, 1H), 2.17 (s, 3H), 2.12 (s, 3H), 2.08 (s, 3H), 2.03 (s, 3H); ^13^C-NMR (101 MHz, CDCl_3_) *δ* 170.8, 170.0, 169.6, 168.1, 137.4, 128.5, 128.0, 127.8, 91.1, 74.1, 71.5, 70.8, 67.1, 66.9, 62.4, 20.9, 20.9, 20.8, 20.8; HRMS (ESI) m/z: [M + Na]+ calcd for C_21_H_26_NaO_10_ 461.1424; found 461.1440.

##### 1-(2,4,6-Tri-*O*-acetyl-3-*O*-benzyl-α-l-mannopyranosyl)thymine 4

To a solution of 3 (14.40 g, 32.8 mmol,) in dry acetonitrile (320 mL) were added thymine (8.12 g, 64.4 mmol) and *N*,*O*-bis(trimethylsilyl)acetamide (32.0 mL, 131 mmol) at room temperature under an argon atmosphere. After refluxing for 1 h, to the reaction mixture was added trimethylsilyl trifluoromethanesulfonate (12.0 mL, 64.4 mmol) at room temperature, and the reaction mixture was refluxed for 15 h. After the addition of saturated aqueous NaHCO_3_ at 0 °C, the resulting mixture was extracted with AcOEt. The obtained organic layer was washed with saturated aqueous NaHCO_3_ and brine, dried over Na_2_SO_4_, and concentrated under reduced pressure. The crude product was purified by flash silica gel column chromatography (*n*-hexane/AcOEt = 2 : 3) to obtain 4 (12.78 g, 25.3 mmol, 77%) as a white solid. ^1^H-NMR (400 MHz, CDCl_3_) *δ* 8.48 (brs, 1H), 7.39–7.30 (m, 5H), 7.15 (brs, 1H), 6.26 (d, *J* = 9.6 Hz, 1H), 5.19 (dd, *J* = 9.6, 3.2 Hz, 1H), 5.03 (d, *J* = 3.7 Hz, 1H), 4.75–4.64 (m, 3H), 4.31–4.27 (m, 2H), 4.02 (t, *J* = 3.2 Hz, 1H), 2.16 (s, 3H), 2.07 (s, 3H), 1.93 (s, 6H); ^13^C-NMR (101 MHz, CDCl_3_) *δ* 170.6, 169.8, 169.7, 163.4, 150.3, 136.7, 135.6, 128.6, 128.4, 128.3, 111.5, 76.4, 75.0, 73.9, 73.6, 68.4, 67.6, 61.0, 21.1, 20.8, 20.7, 12.6; HRMS (ESI) *m*/*z*: [M + Na]^+^ calcd for C_24_H_28_N_2_NaO_10_^+^ 527.1642; found 527.1639.

##### 1-(3-*O*-Benzyl-α-l-mannopyranosyl)thymine 5

To a solution of 4 (11.20 g, 22.2 mmol) in dry methanol (222 mL) was added potassium carbonate (9.20 g, 66.6 mmol) at room temperature under an argon atmosphere. After stirring at room temperature for 30 min, the reaction mixture was filtered. The filtrate was neutralized with Amberlite IRC120 H-form. The mixture was filtered and concentrated under reduced pressure. The crude product was purified by flash silica gel column chromatography (AcOEt, then CHCl_3_/MeOH = 9 : 1) to obtain 5 (7.90 g, 20.9 mmol, 94%) as a white solid. ^1^H-NMR (400 MHz, DMSO-d6) *δ* 11.27 (s, 1H), 7.54 (s, 1H), 7.40–7.27 (m, 5H), 5.81 (d, *J* = 9.6 Hz, 1H), 5.26 (d, *J* = 5.5 Hz, 1H), 5.22 (d, *J* = 6.4 Hz, 1H), 4.71–4.64 (m, 3H), 4.14–4.09 (m, 1H), 3.89–3.69 (m, 4H), 3.64–3.59 (m, 1H), 1.81 (s, 3H); ^13^C-NMR (101 MHz, DMSO-D6) *δ* 163.8, 151.1, 138.7, 137.0, 128.2, 127.5, 127.4, 109.3, 81.3, 80.6, 76.9, 72.5, 66.4, 65.1, 59.4, 48.7, 12.1; HRMS (ESI) *m*/*z*: [M + Na]^+^ calcd for C_18_H_22_N_2_NaO_7_^+^ 401.1325; found 401.1344.

##### 1-{3-*O*-Benzyl-4,6-*O*-(1,1,3,3-tetraisopropyl-1,3-disiloxanediyl)-α-l-mannopyranosyl}thymine 6

To a solution of 5 (7.01 g, 18.5 mmol) in dry pyridine (92.5 mL), 1,3-dichloro-1,1,3,3-tetraisopropyldisiloxane (6.1 mL, 19.5 mmol) was added dropwise at 0 °C under an argon atmosphere, and the reaction mixture was stirred at room temperature for 24 h. After the addition of water at 0 °C, the resulting mixture was extracted with AcOEt. The obtained organic layer was washed with 1M HCl, saturated aqueous NaHCO_3_ and brine, dried over Na_2_SO_4_, and concentrated under reduced pressure. The crude product was purified by flash silica gel column chromatography (*n*-hexane/AcOEt = 3 : 2 to CHCl_3_/MeOH = 9 : 1) to obtain 6 (9.33 g, 15.0 mmol, 81%) as a white solid. ^1^H-NMR (400 MHz, CDCl_3_) *δ* 8.58 (brs, 1H), 7.36–7.29 (m, 5H), 7.08 (d, *J* = 0.9 Hz, 1H), 5.36 (d, *J* = 3.2 Hz, 1H), 4.80 (d, *J* = 11.9 Hz, 1H), 4.68–4.64 (m, 2H), 4.33 (dd, *J* = 7.3, 4.1 Hz, 1H), 4.25 (dd, *J* = 9.4, 7.6 Hz, 1H), 4.08 (dd, *J* = 12.8, 1.8 Hz, 1H), 3.87 (dd, *J* = 12.6, 1.6 Hz, 1H), 3.64–3.61 (m, 1H), 2.96 (d, *J* = 3.2 Hz, 1H), 1.90 (d, *J* = 1.4 Hz, 3H), 1.11–0.96 (m, 28H); ^13^C-NMR (101 MHz, CDCl_3_) *δ* 163.8, 150.7, 139.9, 137.6, 128.6, 128.1, 128.0, 111.4, 89.4, 80.9, 76.4, 73.0, 67.4, 66.7, 60.9, 17.4, 17.3, 17.3, 17.1, 13.7, 13.3, 12.7, 12.5, 12.4; HRMS (ESI) *m*/*z*: [M + Na]^+^ calcd for C_30_H_48_N_2_NaO_8_Si_2_^+^ 643.2847; found 643.2859.

##### 2,2′-Anhydro-1-{3-*O*-benzyl-4,6-*O*-(1,1,3,3-tetraisopropyl-1,3-disiloxanediyl)-α-l-glucopyranosyl}thymine 7

To a solution of 6 (8.76 g, 14.1 mmol) in dry dichloromethane (141 mL) was added 4-dimethylaminopyridine (8.62 g, 70.6 mmol) at room temperature and trifluoromethanesulfonic anhydride (4.6 mL, 28.0 mmol) at –10 °C under an argon atmosphere, and the reaction mixture was stirred at –10 °C for 30 min. After the addition of water at –10 °C, the resulting mixture was extracted with AcOEt. The obtained organic layer was washed with 1M HCl, saturated aqueous NaHCO_3_ and brine, dried over Na_2_SO_4_, and concentrated under reduced pressure to obtain 7 (8.08 g, 13.4 mmol, 95%) as a pale yellow solid. ^1^H-NMR (400 MHz, CDCl_3_) *δ* 7.36–7.28 (m, 5H), 7.14 (d, *J* = 1.4 Hz, 1H), 6.00 (d, *J* = 7.4 Hz, 1H), 4.90 (dd, *J* = 7.3, 3.7 Hz,1H), 4.81 (d, *J* = 11.5 Hz, 1H), 4.75 (d, *J* = 11.9 Hz, 2H), 4.14–4.06 (m, 2H), 3.95–3.91 (m, 2H), 3.32 (brd, *J* = 9.6 Hz, 1H), 1.98 (d, *J* = 0.9 Hz, 3H), 1.09–0.92 (m, 28H); ^13^C-NMR (101 MHz, CDCl_3_) *δ* 172.2, 159.1, 136.9, 129.1, 128.6, 128.2, 128.0, 120.3, 84.7, 82.7, 79.5, 73.7, 73.3, 67.3, 60.3, 17.3, 17.3, 17.3, 17.2, 17.2, 17.1, 14.3, 13.5, 13.2, 12.6, 12.6; HRMS (ESI) *m*/*z*: [M + Na]^+^ calcd for C_30_H_46_N_2_NaO_7_Si_2_^+^ 625.2741; found 625.2735.

##### 2,2′-Anhydro-1-(3-*O*-benzyl-α-l-glucopyranosyl)thymine 8

To a solution of 7 (7.89 g, 13.1 mmol) in dry tetrahydrofuran (131 mL) were added acetic acid (3.0 mL, 52.5 mmol) and 1 M tetrabutylammonium fluoride in tetrahydrofuran (26.2 mL, 26.2 mmol) at room temperature under an argon atmosphere. After stirring at room temperature for 1 h, the reaction mixture was concentrated under reduced pressure. The crude product was purified by flash silica gel column chromatography (CHCl_3_/MeOH = 10 : 1) to obtain 8 (3.52 g, 9.77 mmol, 75%) as a white solid. ^1^H-NMR (400 MHz, CD_3_OD) *δ* 7.70 (d, *J* = 1.4 Hz, 1H), 7.42–7.28 (m, 5H), 6.17 (d, *J* = 6.4 Hz, 1H), 5.11–5.08 (m, 1H), 4.79 and 4.77 (each d *J* = 11.9 Hz, each 1H), 3.95 (t, *J* = 4.1 Hz, 1H), 3.83–3.77 (m, 2H), 3.67 (dd, *J* = 11.9, 6.0 Hz, 1H), 3.29–3.26 (m, 1H), 1.96 (d, *J* = 1.4 Hz, 3H); ^13^C-NMR (101 MHz, CD_3_OD) *δ* 175.4, 161.8, 139.0, 134.0, 129.5, 129.1, 129.0, 119.8, 85.3, 80.1, 79.6, 76.3, 73.6, 67.9, 63.0, 14.0; HRMS (ESI) *m*/*z*: [M + Na]^+^ calcd for C_18_H_20_N_2_NaO_6_^+^ 383.1219; found 383.1202.

##### 2,2′-Anhydro-1-(6-acetylthio-3-*O*-benzyl-6-deoxy-α-l-glucopyranosyl)thymine 9

To a solution of triphenylphosphine (3.30 g, 12.6 mmol) in dry tetrahydrofuran (110 mL), 1.9 M diisopropyl azodicarboxylate in toluene (6.6 mL, 12.5 mmol) was added dropwise over 15 min at 0 °C under an argon atmosphere. After stirring at 0 °C for 30 min, 8 (3.24 g, 8.99 mmol) was added, followed by another 30 min of stirring before the slow addition of thioacetic acid (900 µL, 12.7 mmol) in dry tetrahydrofuran (2.5 mL). The reaction mixture was then stirred at room temperature for 4 h and concentrated under reduced pressure. The crude product was purified by flash silica gel column chromatography (CHCl_3_/MeOH = 30 : 1 to 20 : 1) to obtain 9 (3.00 g, 7.17 mmol, 80%) as a pale yellow solid. ^1^H-NMR (400 MHz, CDCl_3_) *δ* 7.38–7.29 (m, 5H), 7.16 (d, *J* = 0.9 Hz, 1H), 5.96 (d, *J* = 6.8 Hz, 1H), 4.85 (dd, *J* = 6.9, 4.1 Hz, 1H), 4.81 (d, *J* = 11.4 Hz, 1H), 4.77 (d, *J* = 11.4 Hz, 1H), 3.96 (dd, *J* = 6.0, 4.1 Hz, 1H), 3.74 (d, *J* = 4.6 Hz, 1H), 3.69–3.60 (m, 2H), 3.37–3.27 (m, 2H), 2.37 (s, 3H), 1.98 (d, *J* = 0.9 Hz, 3H); ^13^C-NMR (101 MHz, CDCl_3_) *δ* 196.5, 172.4, 159.4, 137.0, 129.8, 128.7, 128.3, 128.1, 119.8, 83.7, 79.6, 79.2, 73.2, 72.8, 69.9, 30.7, 30.6, 14.2; HRMS (ESI) *m*/*z*: [M + Na]^+^ calcd for C_20_H_22_N_2_NaO_6_S^+^ 441.1096; found 441.1084.

##### 1-(2,6-Anhydro-3-*O*-benzyl-2-deoxy-2-thio-α-l-mannopyranosyl)thymine 10

To a solution of 9 (2.86 g, 6.83 mmol) in dry ethanol (137 mL) was added potassium carbonate (1.89 g, 13.7 mmol) at room temperature under an argon atmosphere, and the reaction mixture was refluxed for 40 min. After the addition of H_2_O, the resulting mixture was extracted with AcOEt. The combined organic layer was washed with H_2_O twice and brine, dried over Na_2_SO_4_, and concentrated under reduced pressure. The crude product was purified by flash silica gel column chromatography (CHCl_3_, then CHCl_3_/MeOH = 30 : 1) to obtain 10 (2.42 g, 6.43 mmol, 94%) as a white solid. ^1^H-NMR (400 MHz, CDCl_3_) *δ* 9.15 (brs, 1H), 7.65 (s, 1H), 7.33–7.30 (m, 5H), 6.17 (d, *J* = 1.4 Hz, 1H), 4.69 (d, *J* = 11.4 Hz, 1H), 4.48 (d, *J* = 11.9 Hz, 1H), 4.38 (brs, 1H), 3.90 (brs, 1H), 3.67 (dd, *J* = 2.8, 2.7 Hz, 1H), 3.32 (brs, 1H), 3.23 (dd, *J* = 11.4, 3.2 Hz, 1H), 2.85 (dd, *J* = 11.4, 1.8 Hz, 1H), 2.69 (d, *J* = 3.2 Hz, 1H), 1.86 (s, 3H); ^13^C-NMR (101 MHz, CDCl_3_) *δ* 164.0, 150.2, 136.8, 136.0, 128.7, 128.3, 109.4, 86.4, 77.8, 75.5, 74.4, 71.1, 37.0, 29.3, 12.6; HRMS (ESI) *m*/*z*: [M + Na]^+^ calcd for C_18_H_20_N_2_NaO_5_S^+^ 399.0991; found 399.0998.

##### 1-(2,6-Anhydro-3-*O*-benzyl-2-deoxy-4-*O*-levulinyl-2-thio-α-l-mannopyranosyl)thymine 11

To a solution of 10 (565 mg, 1.50 mmol) in dry dichloromethane (7.5 mL) were added levulinic acid (308 µL, 3.00 mmol), 1-(3-dimethylaminopropyl)-3-ethylcarbodiimide hydrochloride (865.1 mg, 4.51 mmol) and 4-dimethylaminopyridine (92.5 mg, 0.76 mmol) at room temperature under an Ar atmosphere, and the reaction mixture was stirred at room temperature for 3.5 h. After the addition of 1M HCl, the resulting mixture was extracted with AcOEt. The combined organic layer was washed with saturated aqueous NaHCO_3_ and brine, dried over Na_2_SO_4_, and concentrated under reduced pressure. The crude product was purified by flash silica gel column chromatography (*n*-hexane/AcOEt = 1 : 2) to obtain 11 (658.1 mg, 1.39 mmol, 92%) as a pale yellow solid. ^1^H-NMR (400 MHz, CDCl_3_) *δ* 8.52 (brs, 1H), 7.51 (s, 1H), 7.33–7.25 (m, 5H + CHCl_3_), 6.14 (d, *J* = 1.8 Hz, 1H), 4.92 (d, *J* = 3.7 Hz, 1H), 4.59 (d, *J* = 11.9 Hz, 1H), 4.50–4.45 (m, 2H), 3.77 (t, *J* = 3.2 Hz, 1H), 3.34 (t, *J* = 2.3 Hz, 1H), 3.24 (dd, *J* = 11.9, 3.7 Hz, 1H), 2.97 (dd, *J* = 11.9, 1.8 Hz, 1H), 2.92–2.75 (m, 2H), 2.64–2.49 (m, 2H), 2.21 (s, 3H), 1.87 (s, 3H); ^13^C-NMR (101 MHz, CDCl_3_) *δ* 206.4, 171.8, 163.7, 150.0, 136.5, 135.1, 128.5, 128.2, 109.5, 86.3, 77.6, 74.0, 71.6, 71.1, 38.0, 37.0, 29.7, 28.8, 28.0, 12.8; HRMS (ESI) *m*/*z*: [M + Na]^+^ calcd for C_23_H_26_N_2_NaO_7_S^+^ 497.1358; found 497.1356.

##### 1-(2,6-Anhydro-2-deoxy-4-*O*-levulinyl-2-thio-α-l-mannopyranosyl)thymine 12

To a mixture of 20% Pd(OH)_2_/C in 50% H_2_O (260.2 mg) in dry tetrahydrofuran (11.0 mL) was added compound 11 (261.4 mg, 0.55 mmol) at room temperature. The reaction mixture was stirred under a H_2_ atmosphere at room temperature for 18 h, filtered and concentrated under reduced pressure. The crude product was purified by flash silica gel column chromatography (*n*-hexane/AcOEt = 1 : 3) to obtain 12 (206.7 mg, 0.54 mmol, 98%) as a white solid. ^1^H-NMR (400 MHz, CDCl_3_) *δ* 8.40 (brs, 1H), 7.65 (d, *J* = 0.9 Hz, 1H), 6.24 (d, *J* = 1.8 Hz, 1H), 4.62 (brd, *J* = 2.7 Hz, 1H), 4.53 (m, 1H), 4.03 (dt, *J* = 7.8, 3.0 Hz, 1H), 3.37 (dd, *J* = 3.2, 1.8 Hz, 1H), 3.34 (d, *J* = 7.3 Hz, 1H), 3.29 (dd, *J* = 11.9, 3.7 Hz, 1H), 2.93–2.78 (m, 3H), 2.70–2.55 (m, 2H), 2.20 (s, 3H), 1.94 (d, *J* = 1.4 Hz, 3H); ^13^C-NMR (101 MHz, CDCl_3_) *δ* 206.4, 173.0, 163.6, 150.1, 135.2, 109.8, 85.9, 80.4, 70.9, 69.1, 40.1, 38.1, 29.7, 28.8, 28.0, 12.9; HRMS (ESI) *m*/*z*: [M + Na]^+^ calcd for C_16_H_20_N_2_NaO_7_S^+^ 407.0889; found 407.0896.

##### 1-{2,6-Anhydro-3-*O*-{2-cyanoethoxy(diisopropylamino)phosphanyl}-2-deoxy-4-*O*-levulinyl-2-thio-α-l-mannopyranosyl}thymine 13

To a solution of 12 (402 mg, 1.05 mmol) in dry dichloromethane (21.0 mL) were added *N*,*N*-diisopropylethylamine (712 µL, 4.19 mmol) and 2-cyanoethyl *N*,*N*-diisopropylchlorophosphoramidite (467 µL, 2.09 mmol) at room temperature under an Ar atmosphere, and the reaction mixture was stirred at room temperature for 3 h. After the addition of H_2_O, the resulting mixture was extracted with AcOEt. The combined organic layer was washed with saturated aqueous NaHCO_3_ and brine, dried over Na_2_SO_4_, and concentrated under reduced pressure. The crude product was purified by flash silica gel column chromatography (*n*-hexane/AcOEt = 1 : 2) to obtain 13 (514.3 mg, 0.88 mmol, 84%) as a white solid. ^31^P-NMR (162 MHz, CDCl_3_) *δ* 150.3, 147.7; HRMS (ESI) *m*/*z*: [M + Na]^+^ calcd for C_25_H_37_N_4_NaO_8_PS^+^ 607.1967; found 607.1940.

##### 1-(4-*O*-Acetyl-2,6-anhydro-2-deoxy-2-thio-3-*O*-triethysilyl-α-l-mannopyranosyl)thymine 14

To a solution of 10 (938.8 mg, 2.49 mmol) in dry pyridine (83 mL) were added 4-dimethylaminopyridine (143.7 mg, 1.18 mmol) and acetic anhydride (354 µL, 3.74 mmol) at room temperature under an argon atmosphere, and the reaction mixture was stirred at room temperature for 2.5 h. After the addition of H_2_O, the resulting mixture was extracted with AcOEt. The combined organic layer was washed with 1 M HCl, saturated aqueous NaHCO_3_ and brine, dried over Na_2_SO_4_, and concentrated under reduced pressure to obtain the corresponding acetate S25 (946.3 mg) as a white solid. ^1^H-NMR (400 MHz, CDCl_3_)) *δ* 8.89 (brs, 1H), 7.38 (d, *J* = 0.9 Hz, 1H), 7.33–7.22 (m, 5H + CHCl_3_), 6.16 (d, *J* = 1.4 Hz, 1H), 4.92 (d, *J* = 3.2 Hz, 1H), 4.60 (d, *J* = 11.9 Hz, 1H), 4.47–4.42 (m, 2H), 3.73 (t, *J* = 3.2 Hz, 1H), 3.34 (brq, 1H), 3.24 (dd, *J* = 11.9, 3.7 Hz, 1H), 3.00 (dd, *J* = 11.9, 2.3 Hz, 1H), 2.12 (s, 3H), 1.87 (d, *J* = 0.9 Hz, 3H); ^13^C-NMR (101 MHz, CDCl_3_)) *δ* 169.5, 163.5, 149.9, 136.5, 134.8, 128.6, 128.3, 128.2, 109.5, 86.3, 74.0, 72.0, 71.1, 37.0, 28.9, 21.1, 12.8; HRMS (ESI) *m*/*z*: [M + Na]^+^ calcd for C_20_H_22_N_2_NaO_6_S^+^ 441.1096; found 441.1093. To a mixture of 20% Pd(OH)_2_/C in 50% H_2_O (1.85 g) in dry tetrahydrofuran (44 mL) was added a fraction of the obtained acetate (928.5 mg, 2.22 mmol) at room temperature. The reaction mixture was stirred under a H_2_ atmosphere at room temperature for 2 h, filtered and concentrated under reduced pressure. The crude product was subjected to column chromatography, and the corresponding alcohol S26 (706 mg) was obtained as a white solid. ^1^H-NMR (400 MHz, CDCl_3_) *δ* 9.12 (brs, 1H), 7.54 (d, *J* = 0.9 Hz, 1H), 6.22 (d, *J* = 1.8 Hz, 1H), 4.66 (d, *J* = 2.3 Hz, 1H), 4.49 (brt, 1H), 4.01–3.98 (m, 1H), 3.43 (d, *J* = 7.8 Hz, 1H), 3.37 (q, *J* = 1.7 Hz, 1H), 3.27 (dd, *J* = 11.9, 3.7 Hz, 1H), 2.93 (dd, *J* = 12.4, 2.3 Hz, 1H), 2.20 (s, 3H), 1.95 (d, *J* = 0.9 Hz, 3H); ^13^C-NMR (101 MHz, CDCl_3_) *δ* 170.5, 163.7, 150.1, 135.0, 109.7, 85.9, 80.0, 71.1, 69.1, 40.2, 28.8, 21.0, 12.9; HRMS (ESI) *m*/*z*: [M + Na]^+^ calcd for C_13_H_16_N_2_NaO_6_S^+^ 351.0627; found 351.0642. To a solution of a fraction of the obtained alcohol (662 mg, 2.01 mmol) in dry DMF (10 mL) were added imidazole (275.8 mg, 4.05 mmol) and chlorotriethylsilane (406 µL, 2.42 mmol, 1.2 equiv.) at room temperature under an Ar atmosphere, and the reaction mixture was stirred at room temperature for 30 min. After the addition of saturated aqueous NaHCO_3_, the resulting mixture was extracted with AcOEt. The combined organic layer was washed with brine, dried over Na_2_SO_4_, and concentrated under reduced pressure. The crude product was purified by flash silica gel column chromatography (*n*-hexane/AcOEt = 3 : 2) to obtain 14 (710.1 mg, 1.60 mmol, 80%) as a white solid. ^1^H-NMR (400 MHz, CDCl_3_) *δ* 8.89 (brs, 1H), 7.55 (d, *J* = 1.4 Hz, 1H), 6.17 (d, *J* = 1.4 Hz, 1H), 4.79 (d, *J* = 3.2 Hz, 1H), 4.44 (t, *J* = 2.5 Hz, 1H), 4.10 (t, *J* = 3.2 Hz, 1H), 3.23 (dd, *J* = 11.9, 3.7 Hz, 1H), 3.11 (q, *J* = 1.5 Hz, 1H), 2.99 (dd, *J* = 12.1, 2.5 Hz, 1H), 2.18 (s, 3H), 1.98 (d, *J* = 1.4 Hz, 3H), 0.87 (t, *J* = 8.0 Hz, 9H), 0.52 (q, *J* = 7.9 Hz, 6H); ^13^C-NMR (101 MHz, CDCl_3_) *δ* 169.8, 163.6, 149.9, 135.0, 109.6, 86.5, 80.1, 72.3, 69.5, 41.3, 29.1, 21.0, 12.8, 6.5, 4.6; HRMS (ESI) *m*/*z*: [M + Na]^+^ calcd for C_19_H_30_N_2_NaO_6_SSi^+^ 465.1492; found 465.1480.

##### 1-(4-*O*-Acetyl-2,6-anhydro-2-deoxy-2-thio-3-*O*-triethysilyl-α-l-mannopyranosyl)-5-methylcytosine 15

To a mixture of 1,2,4-triazole (2.67 g, 38.7 mmol) in dry acetonitrile (12 mL), phosphoryl chloride (832 µL, 9.12 mmol) was added dropwise at 0 °C under an Ar atmosphere. After stirring at 0 °C for 5 min, to the reaction mixture was slowly added triethylamine (6.3 mL, 45.4 mmol), and stirred at 0 °C for 20 min. To the reaction mixture was added dropwise a solution of 14 (504 mg, 1.14 mmol) in dry acetonitrile (11 mL) over 5 min, and the reaction mixture was stirred at room temperature for 1 h. After the addition of saturated aqueous NaHCO_3_, the resulting mixture was extracted with AcOEt. The combined organic layer was washed with brine, dried over Na_2_SO_4_, and concentrated under reduced pressure to obtain the residue. The residue was dissolved in a solution of 28% aqueous ammonia (8 mL) and dioxane (15 mL), and the reaction mixture was stirred at room temperature for 40 min. After the addition of H_2_O, the resulting mixture was extracted with AcOEt. The combined organic layer was washed with brine, dried over Na_2_SO_4_, and concentrated under reduced pressure to obtain 15 (454 mg, 1.03 mmol, 90%) as a white solid. ^1^H-NMR (400 MHz, CDCl_3_) *δ* 7.54 (brs, 1H), 6.19 (d, *J* = 1.4 Hz, 1H), 4.77 (d, *J* = 3.7 Hz, 1H), 4.42 (t, *J* = 2.8 Hz, 1H), 4.06 (t, *J* = 3.2 Hz, 1H), 3.27 (q, *J* = 1.4 Hz, 1H), 3.23 (dd, *J* = 11.9, 3.2 Hz, 1H), 2.97 (dd, *J* = 11.9, 2.3 Hz, 1H), 2.17 (s, 3H), 1.98 (s, 3H), 0.84 (t, *J* = 7.8 Hz, 9H), 0.48 (q, *J* = 7.9 Hz, 6H); ^13^C-NMR (101 MHz, CDCl_3_) *δ* 169.9, 165.7, 155.6, 137.5, 100.8, 86.7, 80.3, 72.2, 69.5, 41.1, 29.1, 21.0, 13.5, 6.5, 4.6; HRMS (ESI) *m*/*z*: [M + Na]^+^ calcd for C_19_H_31_N_3_NaO_5_SSi^+^ 464.1651; found 464.1633.

##### 1-(2,6-Anhydro-2-deoxy-2-thio-3-*O*-triethysilyl-α-l-mannopyranosyl)-4-*N*-benzoyl-5-methylcytosine 16

A solution of 15 (433.1 mg, 0.98 mmol) in 4% ammonia in methanol (20.0 mL) was stirred at room temperature for 3 h and concentrated under reduced pressure to the residue. To the solution of the obtained residue in dry pyridine (11.4 mL) was added benzoic anhydride (268.1 mg, 1.19 mmol) at room temperature under an Ar atmosphere. After stirring at room temperature for 24 h, to the reaction mixture was added benzoic anhydride (103.1 mg, 0.46 mmol), and the reaction mixture was stirred at room temperature for 26 h. After the addition of H_2_O, the resulting mixture was extracted with AcOEt. The combined organic layer was washed with brine, dried over Na_2_SO_4_, and concentrated under reduced pressure. The crude product was purified by flash silica gel column chromatography (*n*-hexane/AcOEt = 3 : 1) to obtain 16 (205.5 mg, 0.41 mmol, 42%) as a white solid. ^1^H-NMR (400 MHz, CDCl_3_) *δ* 8.35–8.32 (m, 2H), 7.93 (d, *J* = 1.4 Hz, 1H), 7.56–7.51 (m, 1H), 7.47–7.43 (m, 2H), 6.23 (d, *J* = 1.8 Hz, 1H), 4.40 (t, *J* = 2.5 Hz, 1H), 3.96 (t, *J* = 3.0 Hz, 1H), 3.83 (brt, 1H), 3.26 (dd, *J* = 11.7, 3.4 Hz, 1H), 3.13 (q, *J* = 1.7 Hz, 1H), 2.87 (dd, *J* = 11.9, 2.3 Hz, 1H), 2.16 (d, *J* = 0.9 Hz, 3H), 0.90 (t, *J* = 8.0 Hz, 9H), 0.56 (q, *J* = 7.9 Hz, 6H); ^13^C-NMR (101 MHz, CDCl_3_) *δ* 179.6, 159.9, 147.9, 137.4, 137.1, 132.6, 130.2, 129.9, 128.5, 128.2, 110.7, 87.0, 78.0, 75.0, 72.8, 41.1, 29.4, 13.8, 6.7, 4.8; HRMS (ESI) *m*/*z*: [M + Na]^+^ calcd for C_24_H_33_N_3_NaO_5_SSi^+^ 526.1808; found 526.1819.

##### 1-(2,6-Anhydro-2-deoxy-4-*O*-levulinyl-2-thio-α-l-mannopyranosyl)-4-*N*-benzoyl-5-methylcytosine 17

To a solution of 16 (251.4 mg, 0.50 mmol) in dry dichloromethane (10 mL) were added 4-dimethylaminopyridine (61.4 mg, 0.50 mmol), 1-(3-dimethylaminopropyl)-3-ethylcarbodiimide (265 µL, 1.50 mmol) and levulinic acid (102 µL, 1.00 mmol) at room temperature under an Ar atmosphere, and the reaction mixture was stirred at room temperature for 1 h. After the addition of H_2_O, the resulting mixture was extracted with AcOEt. The combined organic layer was washed with 1M HCl, saturated aqueous NaHCO_3_ and brine, dried over Na_2_SO_4_, and concentrated under reduced pressure to obtain the corresponding ester S27 (298.8 mg, quant.) as a colourless, transparent oil. ^1^H-NMR (400 MHz, CDCl_3_) *δ* 8.35–8.32 (m, 2H), 7.85 (d, *J* = 0.9 Hz, 1H), 7.55–7.52 (m, 1H), 7.47–7.44 (m, 2H), 6.21 (d, *J* = 1.8 Hz, 1H), 4.78 (d, *J* = 3.2 Hz, 1H), 4.50 (t, *J* = 2.5 Hz, 1H), 4.12 (t, *J* = 3.2 Hz, 1H), 3.27–3.23 (m, 2H), 2.99–2.89 (m, 2H), 2.83–2.67 (m, 2H), 2.58 (td, *J* = 11.0, 5.5 Hz, 1H), 2.23 (s, 3H), 2.17 (d, *J* = 0.9 Hz, 3H), 0.87 (t, *J* = 8.0 Hz, 9H), 0.52 (q, *J* = 7.9 Hz, 6H); ^13^C-NMR (101 MHz, CDCl_3_) *δ* 206.3, 172.1, 159.7, 147.7, 137.1, 136.5, 132.6, 129.9, 128.2, 110.7, 86.9, 80.2, 71.9, 69.4, 40.8, 38.0, 29.8, 29.0, 28.0, 13.9, 6.5, 4.6; HRMS (ESI) *m*/*z*: [M + Na]^+^ calcd for C_29_H_39_N_3_NaO_7_SSi^+^ 624.2176; found 624.2164. To a solution of a fraction of the obtained ester (280.5 mg, 0.47 mmol) in dry tetrahydrofuran (4.70 mL) was added triethylamine trihydrofluoride (228 µL, 1.40 mmol) at room temperature under an Ar atmosphere, and the reaction mixture was stirred at room temperature for 1 h. After the addition of H_2_O, the resulting mixture was extracted with AcOEt. The combined organic layer was washed with saturated aqueous NaHCO_3_ and brine, dried over Na_2_SO_4_, and concentrated under reduced pressure. The crude product was purified by flash silica gel column chromatography (*n*-hexane/AcOEt = 1 : 1 to 1 : 2) to obtain 17 (226 mg, 0.46 mmol, quant.) as a white solid. ^1^H-NMR (400 MHz, CDCl_3_) *δ* 1H-NMR *δ* 8.32 (d, *J* = 7.3 Hz, 2H), 7.82 (d, *J* = 0.9 Hz, 1H), 7.55–7.51 (m, 1H), 7.45–7.42 (m, 2H), 6.30 (d, *J* = 1.8 Hz, 1H), 4.63 (d, *J* = 2.3 Hz, 1H), 4.56 (brs, 1H), 4.02 (dt, *J* = 7.3, 3.1 Hz, 1H), 3.45 (dd, *J* = 3.2, 1.8 Hz, 1H), 3.31 (dd, *J* = 12.4, 3.6 Hz, 1H), 3.27 (d, *J* = 7.4 Hz, 1H), 2.94–2.79 (m, 3H), 2.73–2.57 (m, 2H), 2.22 (s, 3H), 2.14 (d, *J* = 0.9 Hz, 3H); ^13^C-NMR (101 MHz, CDCl_3_) *δ* 206.4, 173.0, 159.7, 147.9, 137.1, 136.4, 132.5, 129.9, 128.2, 110.9, 86.3, 80.4, 70.9, 69.0, 39.9, 38.1, 29.7, 28.8, 28.0, 14.0; HRMS (ESI) *m*/*z*: [M + Na]^+^ calcd for C_23_H_25_N_3_NaO_7_S^+^ 510.1288; found 510.1311.

##### 1-{2,6-Anhydro-3-*O*-{2-cyanoethoxy(diisopropylamino)phosphanyl}-2-deoxy-4-*O*-levulinyl-2-thio-α-l-mannopyranosyl}-4-*N*-benzoyl-5-methylcytosine 18

To a solution of 17 (146.6 mg, 0.30 mmol) in dry dichloromethane (6.0 mL) were added *N*,*N*-diisopropylethylamine (205 µL, 1.21 mmol) and 2-cyanoethyl *N*,*N*-diisopropylchlorophosphoramidite (134 µL, 0.60 mmol) at room temperature under an Ar atmosphere, and the reaction mixture was stirred at room temperature for 1.5 h. After the addition of H_2_O, the resulting mixture was extracted with AcOEt. The combined organic layer was washed with saturated aqueous NaHCO_3_ and brine, dried over Na_2_SO_4_, and concentrated under reduced pressure. The crude product was purified by flash silica gel column chromatography (*n*-hexane/AcOEt = 3 : 2) to obtain 19 (163.8 mg, 0.24 mmol, 79%) as a colorless, transparent oil. ^31^P-NMR (162 MHz, CDCl_3_) *δ* 150.5, 147.9; HRMS (ESI) *m*/*z*: [M + Na]^+^ calcd for C_32_H_42_N_5_NaO_8_PS^+^ 710.2389; found 710.2403.

#### Solid-phase synthesis, purification and characterization of l-*lyxo*-thioBsNA-modified oligonucleotides

Synthesis of oligonucleotides were performed using a phosphoramidite coupling protocol with a syringe only for coupling with l-*lyxo*-thioBsNA, and the others were performed on a 1.0 µmol scale using an automated DNA synthesizer. 0.25 M 5-Benzylthio-1*H*-tetrazole acetonitrile solution was used as the activator. 3% (w/v) Trichloroacetic acid dichloromethane solution was used as the detritylating agent. 0.5 M Hydrazine monohydrate in pyridine/AcOH [3 : 2(v/v)] solution was used as the delevulinoylating agent. 0.02 M Iodine THF/water/pyridine [78 : 20 : 2(v/v/v)] solution was used as the oxidant. The oxidation was performed for a total of 18 s (delivered in two steps of 10 s and 8 s). 10 vol% Acetic anhydride THF solution and 1-methylimidazole/pyridine/THF [1 : 1 : 8(v/v/v)] solution were used as capping reagents. The concentration of each phosphoramidite was 0.075 M, and the coupling times were 10 s (2 cycles) for natural deoxynucleic acids and 12 min for l-*lyxo*-thioBsNA. Cleavage from the solid support and removal of all protecting groups were performed using 28% aqueous ammonia at 55 °C for 16 h. The resulting DMTr-on oligonucleotides were briefly purified using a Sep-Pak Plus C18 cartridge, and the DMTr group was removed using 1% aqueous trifluoroacetic acid in the cartridge. Oligonucleotides were further purified using reversed-phase HPLC (Waters XBridge Oligonucleotide BEH C18 OBD column 2.5 µm, 10 × 50 mm). The purity of the purified oligonucleotides was analyzed by reversed-phase HPLC (Waters XBridge Oligonucleotide BEH C18 column 2.5 µm, 4.6 ×50 mm). For HPLC analysis, Shimadzu DGU-20A3R/20A5R, LC-20AT, SIL-20AC HT, CBM-20A, CTO-20AC, SPD-20A, and FRC-10A instruments were used. The compositions were confirmed by MALDI-TOF mass spectrometry (Bruker ultrafleXtreme) using 3-hydroxypicolinic acid as the matrix. Isolated yields were determined by the UV absorbance at 260 nm using a DeNovix NanoPad DS-11+ spectrophotometer.

#### UV melting experiments

Melting temperatures (*T*_m_) were determined using a Shimadzu UV-1800 spectrophotometer equipped with a TMSPC-8 *T*_m_ analysis accessory. For the UV melting experiments of the duplexes with target single-stranded RNA or DNA, oligonucleotides were dissolved in 10 mM sodium phosphate buffer (pH 7.2) containing 100 mM NaCl to achieve a final concentration of 4.0 µM for each strand. The samples were annealed by heating at 100 °C, followed by slow cooling to room temperature. The absorbance at 260 nm was recorded at a scan rate of 0.5 °C min^−1^ from 5 to 90 °C. *T*_m_ values were determined from the temperatures at which half of the duplexes dissociated according to the sigmoidal melting curves.

#### Nuclease resistance study

Oligonucleotides (400 pmol) were dissolved in 50 mM Tris-HCl buffer (pH 8.0, 90 µL) containing 10 mM MgCl_2_. To each sample, 25 mU of phosphodiesterase I from *Crotalus adamanteus* venom in 10 µL of water was added, followed by incubation at 37 °C. Aliquots (10 µL) were taken at 0, 2.5, 5.0, 10, 20, and 40 min, heated at 90 °C for 2 min to deactivate the enzyme, and analyzed using reversed-phase HPLC (Waters XBridge Oligonucleotide BEH C18 column 2.5 µm, 4.6 × 50 mm) to assess the intact oligonucleotide proportion.

## Author contributions

MI, SM and NY performed the chemical synthesis supervised by TK. MI and SM performed the evaluation of oligonucleotides supervised by HH and TK. The manuscript was prepared by MI and TK.

## Conflicts of interest

There are no conflicts to declare.

## Supplementary Material

CB-OLF-D6CB00034G-s001

## Data Availability

Supplementary information (SI): synthetic procedures, ^1^H-, ^13^C- and ^31^P-NMR spectra of new compounds, yields and MALDI-TOF-MS data for modified oligonucleotides, and UV-melting curves. See DOI: https://doi.org/10.1039/d6cb00034g.
