# Supplementary material for: Synthesis and properties of l-lyxo-thioBsNA-pyrimidine nucleoside: a sulfur-containing analogue of boat-shaped pyranosyl nucleic acid
Source: RSC Chem Biol. 2026 Mar 25;7(5):830–40. doi: 10.1039/d6cb00034g (PMC13014300; doi:10.1039/d6cb00034g)

Supplementary Information (SI) for RSC Chemical Biology.
This journal is © The Royal Society of Chemistry 2026

Supplementary Information

Table of Contents

1. Supplementary data
2. ^{1}H NMR, ^{13}C NMR and ^{31}P NMR spectra of compounds
3. Characterization data (HPLC and mass data) of synthesized oligonucleotides

1. Supplementary data

Table S1. Isolated yields of oligonucleotides

	Sequences (5’-3’)[a]	Yield[b]
**ON1**	GCG TTT TTT GCT	24%
**ON2**	GCG TT**T** TTT GCT	39%
**ON3**	GCG TT**T** T**T**T GCT	33%
**ON4**	GCG TT**T** TT**T** GCT	34%
**ON5**	GCG **T**T**T** T**T**T GCT	21%
**ON6**	GCG TT**T** **TT**T GCT	24%
**ON7**	GCG TTC TTT GCT	44%
**ON8**	GCG TT**C** TTT GCT	16%
**ON9**	TTT TTT TTT T	23%
**ON10**	TTT TTT TTT^T	32%
**ON11**	TTT TTT TTT **T**	23%
**ON12**	TTT TTT TT**T** T	14%

[a] Capital letters and Bold letters indicate DNA and L-lyxo-thioBsNA, respectively. Mark “^” indicate phosphorothioate. [b] The isolatedyield for **ON1**–**ON12** were calculated from the UV absorbance at 260 nm.

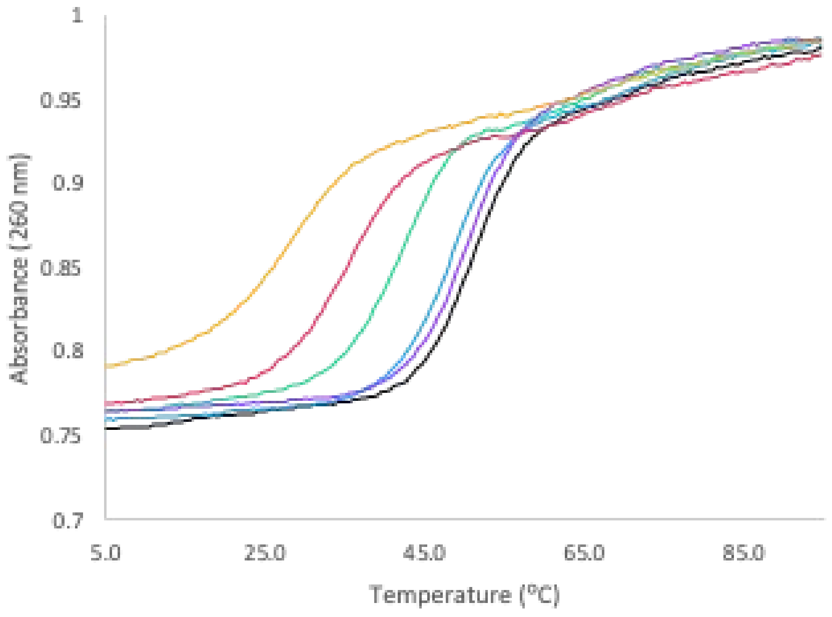

Figure S1. UV melting studies for ONs against complementary DNA. **ON1** black, **ON2** blue, **ON3** green, **ON4** violet, **ON5** pink, and **ON6** orange. Conditions: 10 mM sodium phosphate (pH 7.2), 100 mM NaCl, 4.0 µM of each oligonucleotide, and 4.0 µM of complementary RNA. The curves shown are representative of three independent repeats, each consisting of at least three technical repeats.

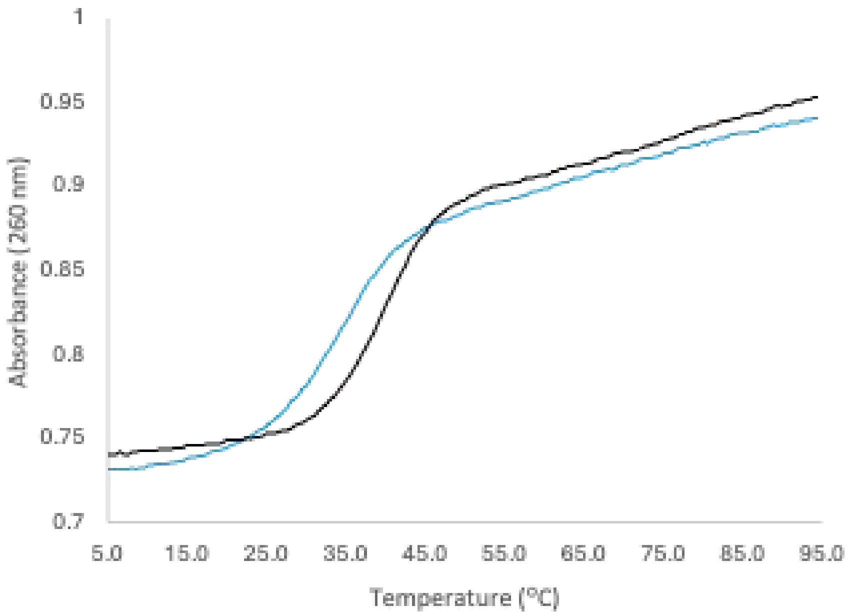

Figure S2. UV melting studies for ONs against G mismatch DNA. **ON1** black, and **ON2** blue. Conditions: 10 mM sodium phosphate (pH 7.2), 100 mM NaCl, 4.0 µM of each oligonucleotide, and 4.0 µM of complementary RNA. The curves shown are representative of three independent repeats, each consisting of at least three technical repeats.

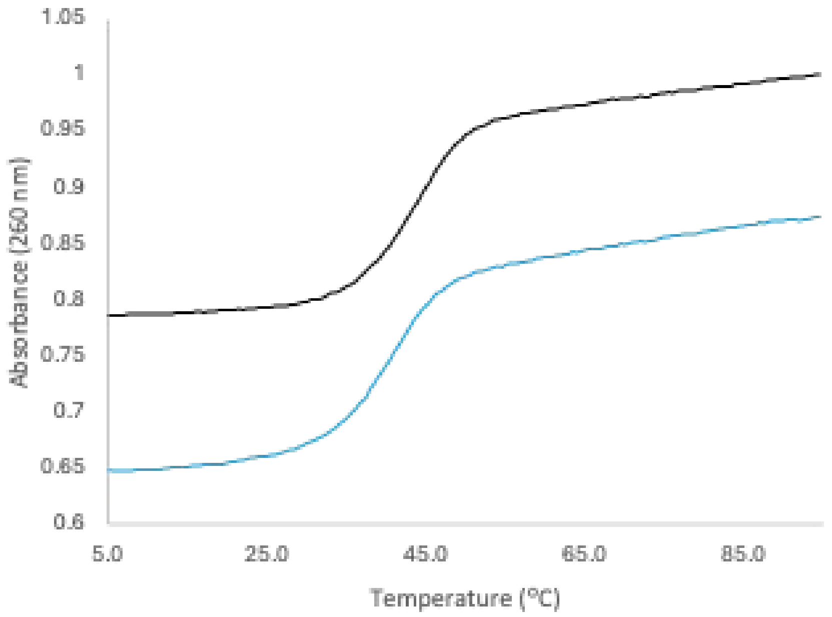

Figure S3. UV melting studies for ONs against G mismatch RNA. **ON1** black, and **ON2** blue. Conditions: 10 mM sodium phosphate (pH 7.2), 100 mM NaCl, 4.0 µM of each oligonucleotide, and 4.0 µM of complementary RNA. The curves shown are representative of three independent repeats, each consisting of at least three technical repeats.

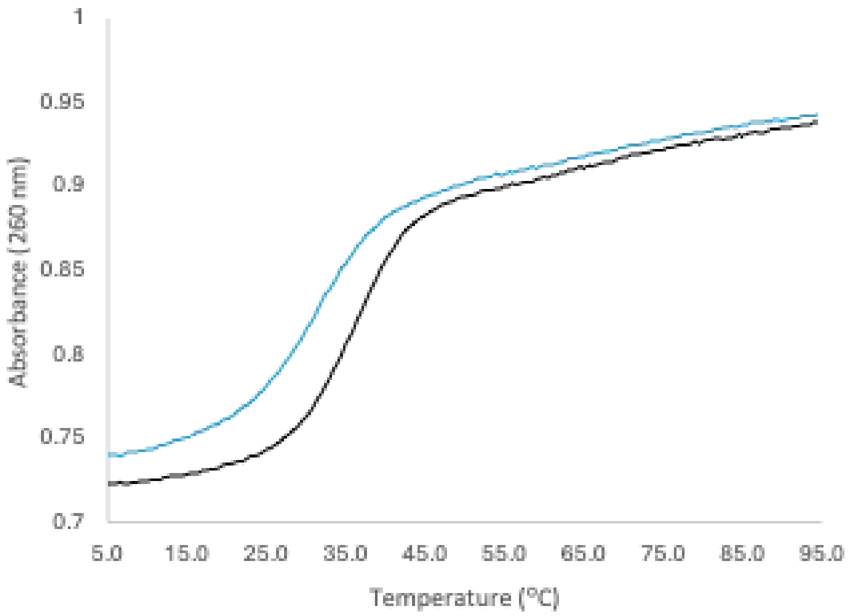

Figure S4. UV melting studies for ONs against C mismatch DNA. **ON1** black, and **ON2** blue. Conditions: 10 mM sodium phosphate (pH 7.2), 100 mM NaCl, 4.0 µM of each oligonucleotide, and 4.0 µM of complementary RNA. The curves shown are representative of three independent repeats, each consisting of at least three technical repeats.

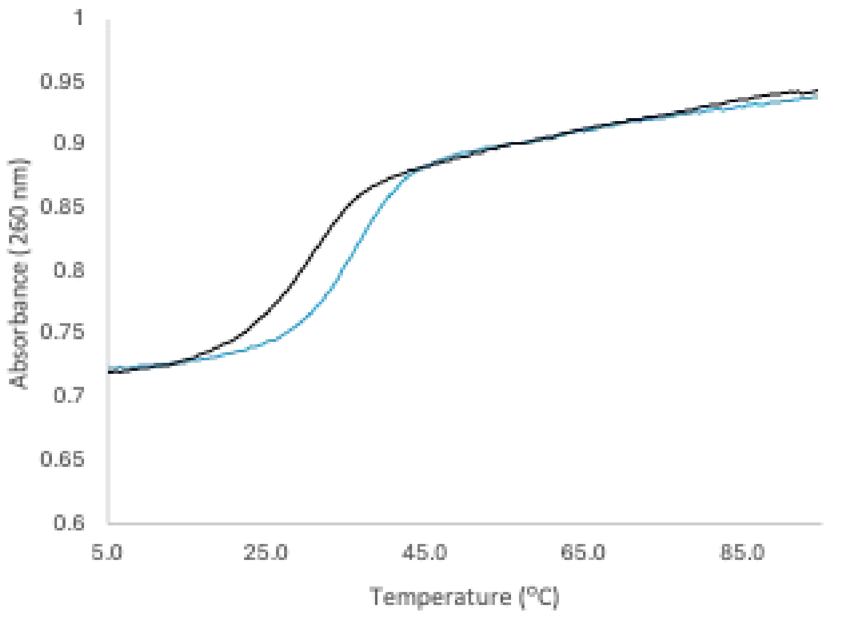

Figure S5. UV melting studies for ONs against C mismatch RNA. **ON1** black, and **ON2** blue. Conditions: 10 mM sodium phosphate (pH 7.2), 100 mM NaCl, 4.0 µM of each oligonucleotide, and 4.0 µM of complementary RNA. The curves shown are representative of three independent repeats, each consisting of at least three technical repeats.

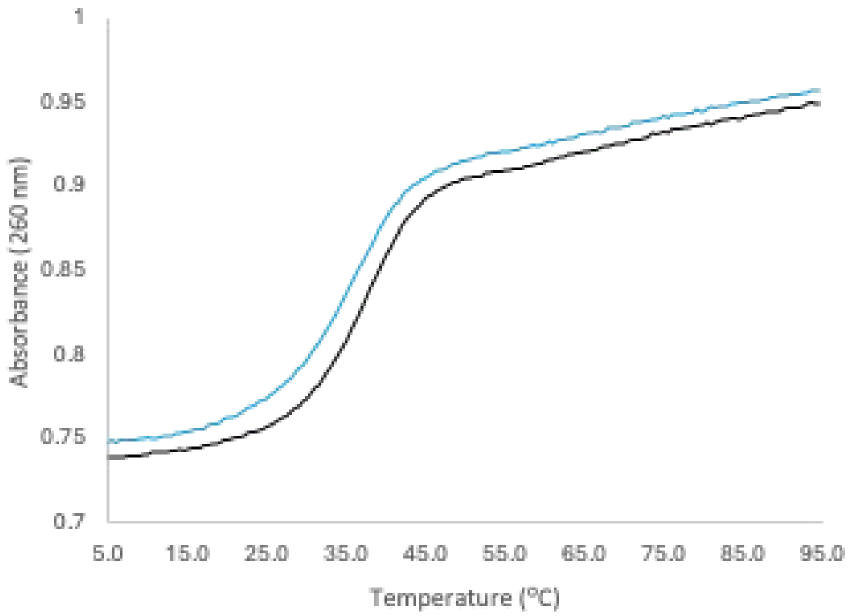

Figure S6. UV melting studies for ONs against T mismatch DNA. **ON1** black, and **ON2** blue. Conditions: 10 mM sodium phosphate (pH 7.2), 100 mM NaCl, 4.0 µM of each oligonucleotide, and 4.0 µM of complementary RNA. The curves shown are representative of three independent repeats, each consisting of at least three technical repeats.

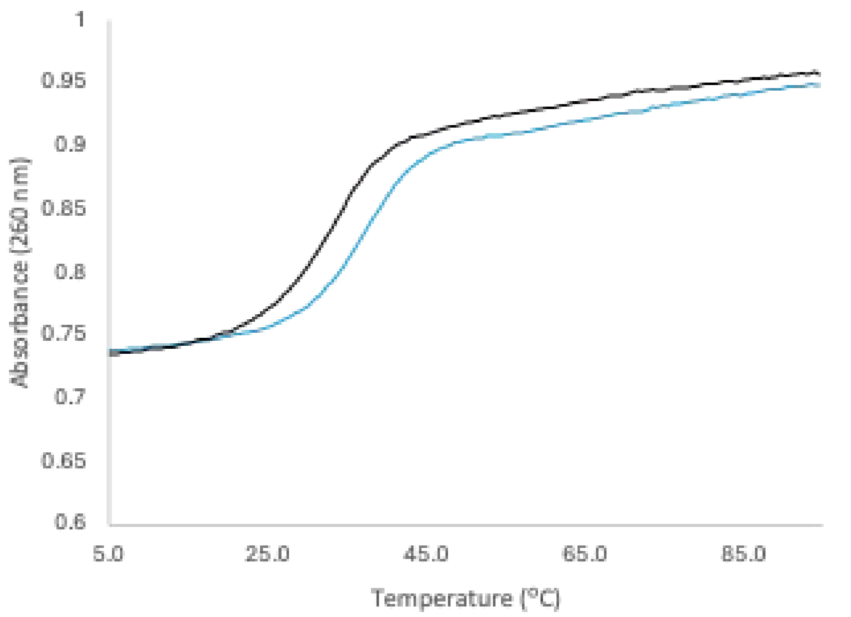

Figure S7. UV melting studies for ONs against U mismatch RNA. **ON1** black, and **ON2** blue. Conditions: 10 mM sodium phosphate (pH 7.2), 100 mM NaCl, 4.0 µM of each oligonucleotide, and 4.0 µM of complementary RNA. The curves shown are representative of three independent repeats, each consisting of at least three technical repeats.

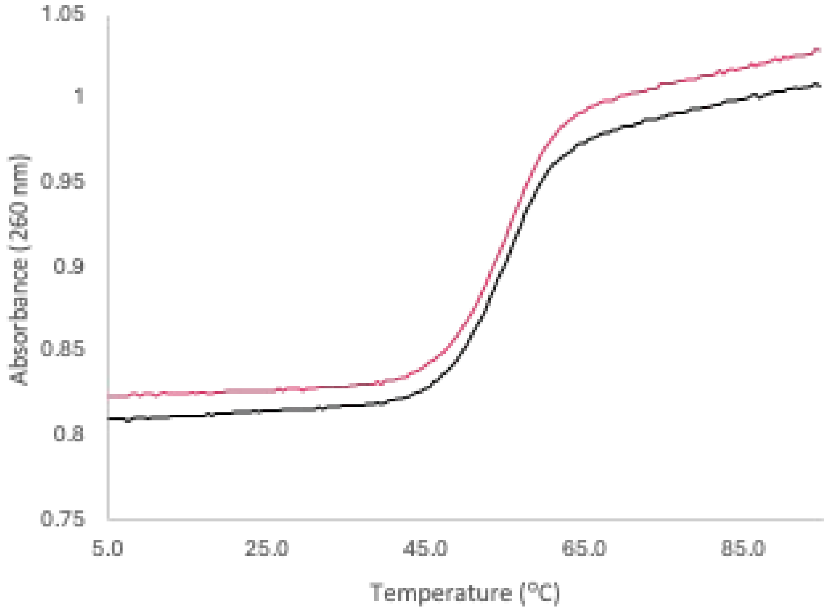

Figure S8. UV melting studies for ONs against complementary DNA. **ON7** black, and **ON8** pink. Conditions: 10 mM sodium phosphate (pH 7.2), 100 mM NaCl, 4.0 µM of each oligonucleotide, and 4.0 µM of complementary RNA. The curves shown are representative of three independent repeats, each consisting of at least three technical repeats.

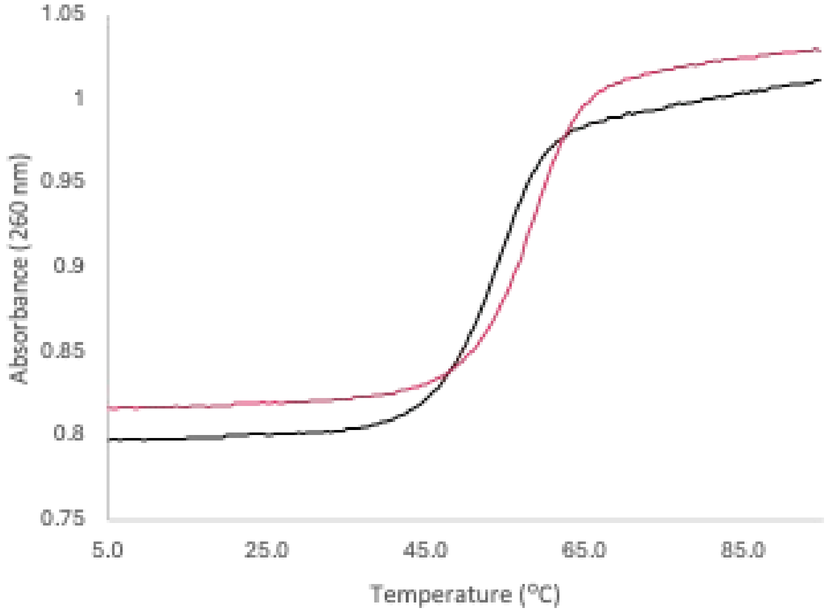

Figure S9. UV melting studies for ONs against complementary RNA. **ON7** black, and **ON8** pink. Conditions: 10 mM sodium phosphate (pH 7.2), 100 mM NaCl, 4.0 µM of each oligonucleotide, and 4.0 µM of complementary RNA. The curves shown are representative of three independent repeats, each consisting of at least three technical repeats.

ON10

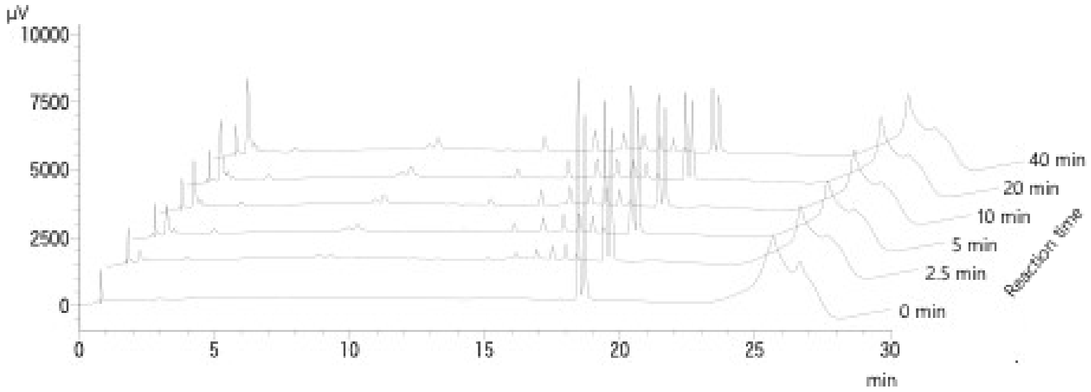

ON11

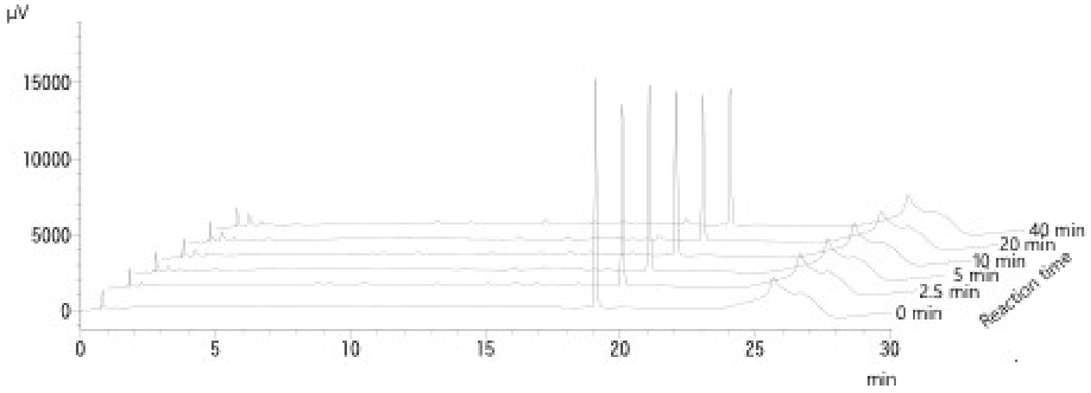

ON12

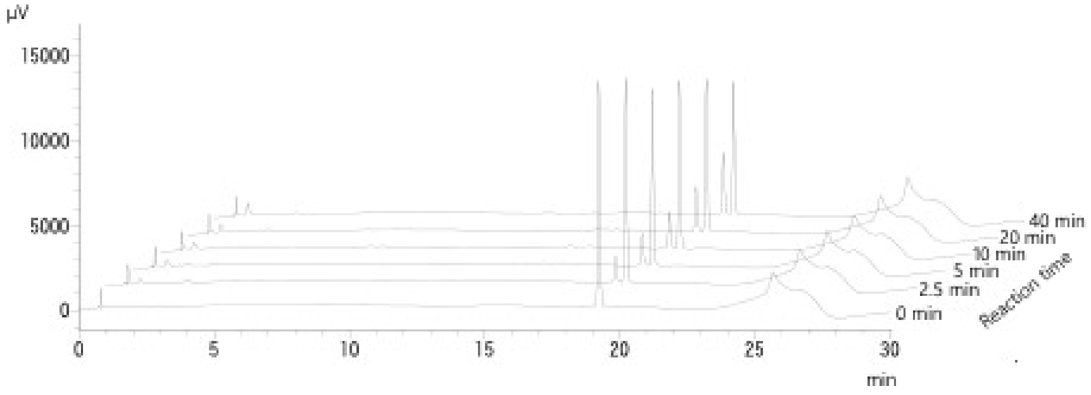

Figure S10. Reverse-phase HPLC analysis of the nuclease stability of **ON10**, **ON11**, and **ON12** against svPDE.. Conditions: 50 mM Tris–HCl (pH 8.0), 10 mM $MgCl_2$, 4.0 µM of each oligonucleotide, and 25 mU svPDE at 37 °C. The samples were analyzed using a reverse-phase HPLC column (Waters XBridge Oligonucleotide BEH C18 column 2.5 µm, 4.6 x 50 mm; eluent A (0.1 M TEAA buffer) and eluent B (MeOH), eluted with a linear gradient (5%–25% of eluent B in 20 min) and UV detection at 260 nm.

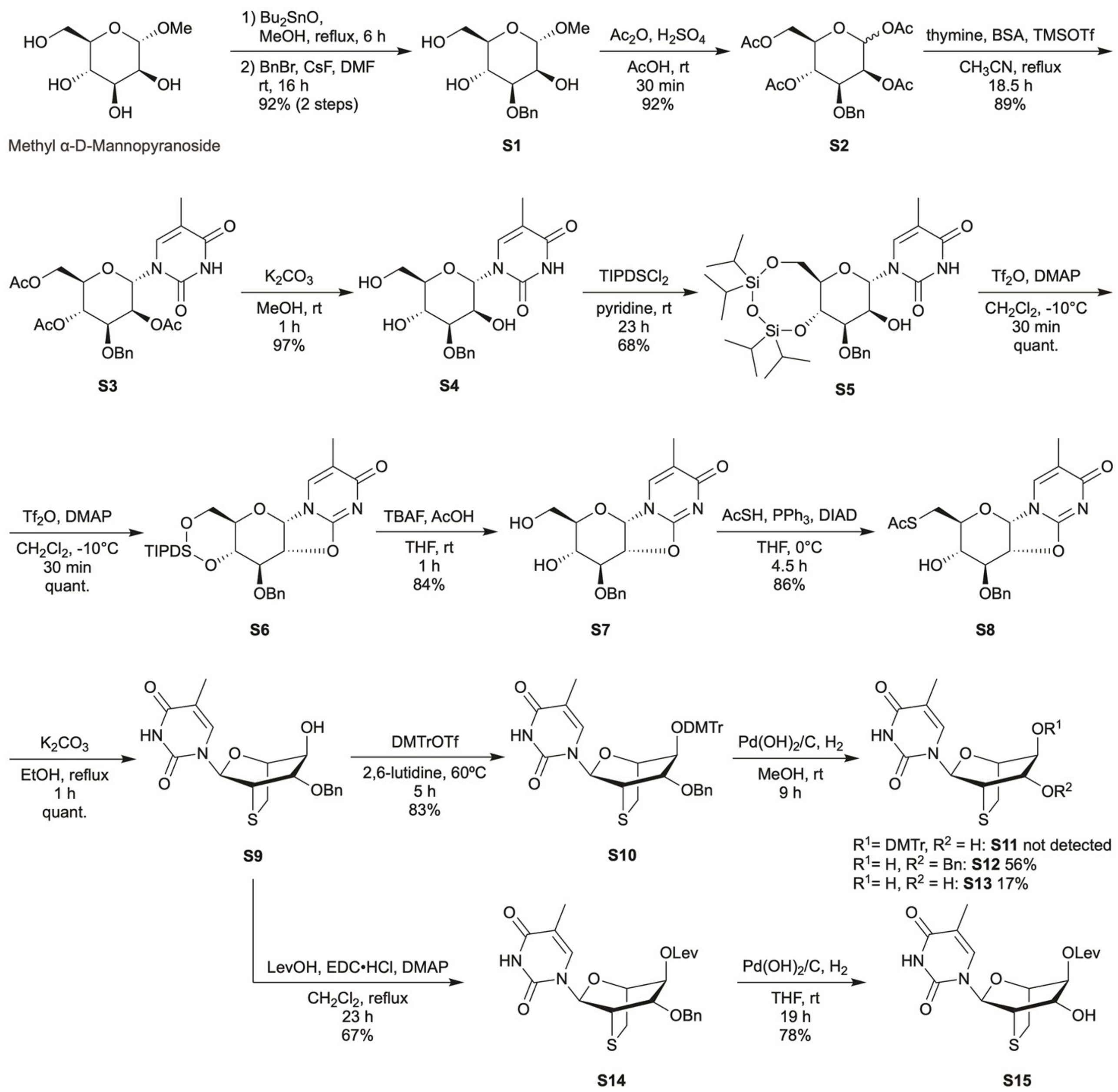

Scheme S1. Synthesis of D-*lyxo*-thioBsNA-T phosphoroamidite precursor

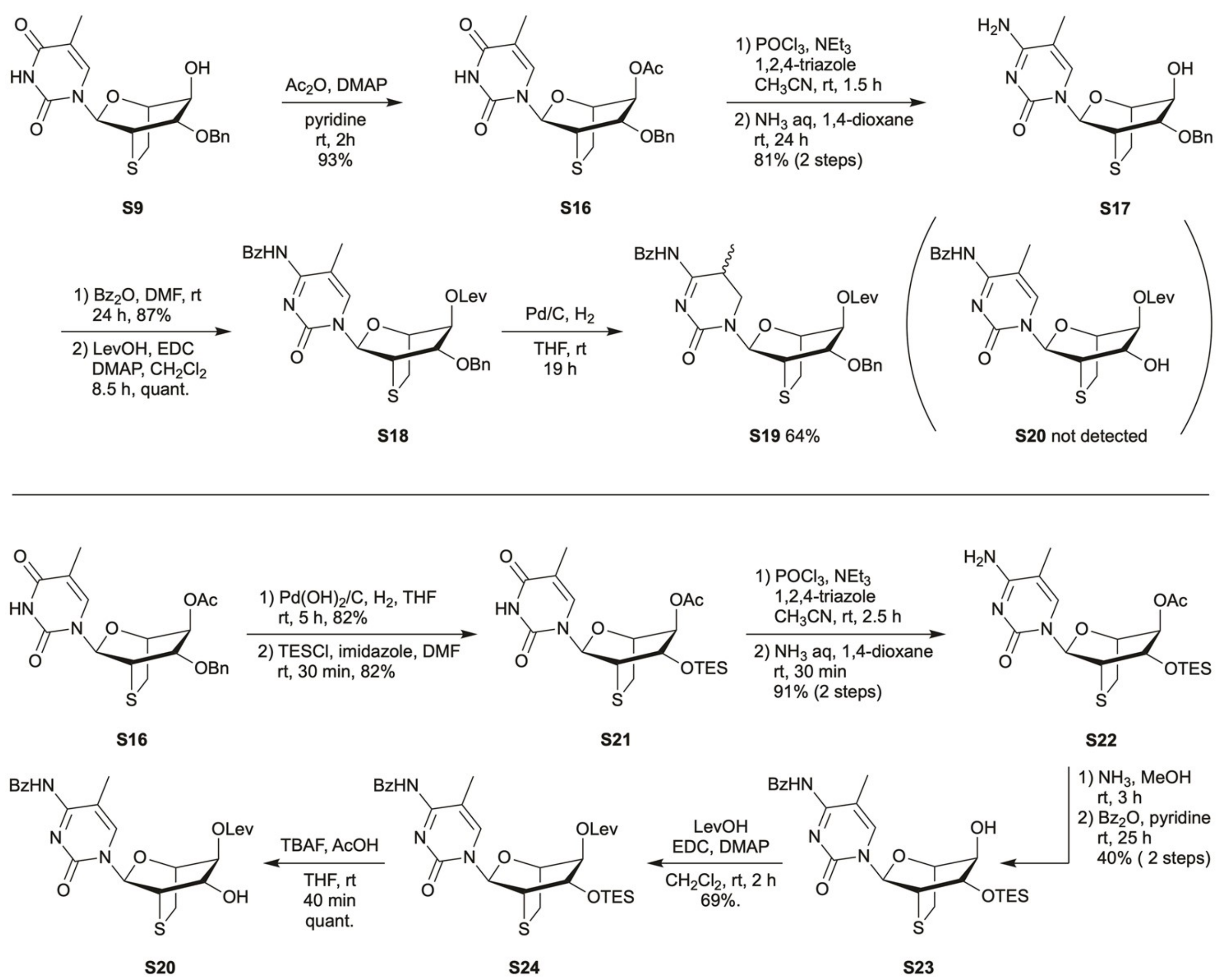

Scheme S2. Synthesis of D-*lyxo*-thioBsNA-meC phosphoroamidite precursor

2. ^{1}H NMR, ^{13}C NMR and ^{31}P NMR spectra of compounds

Compound **1** (^{1}H-NMR, DMSO-D6, 400 MHz)

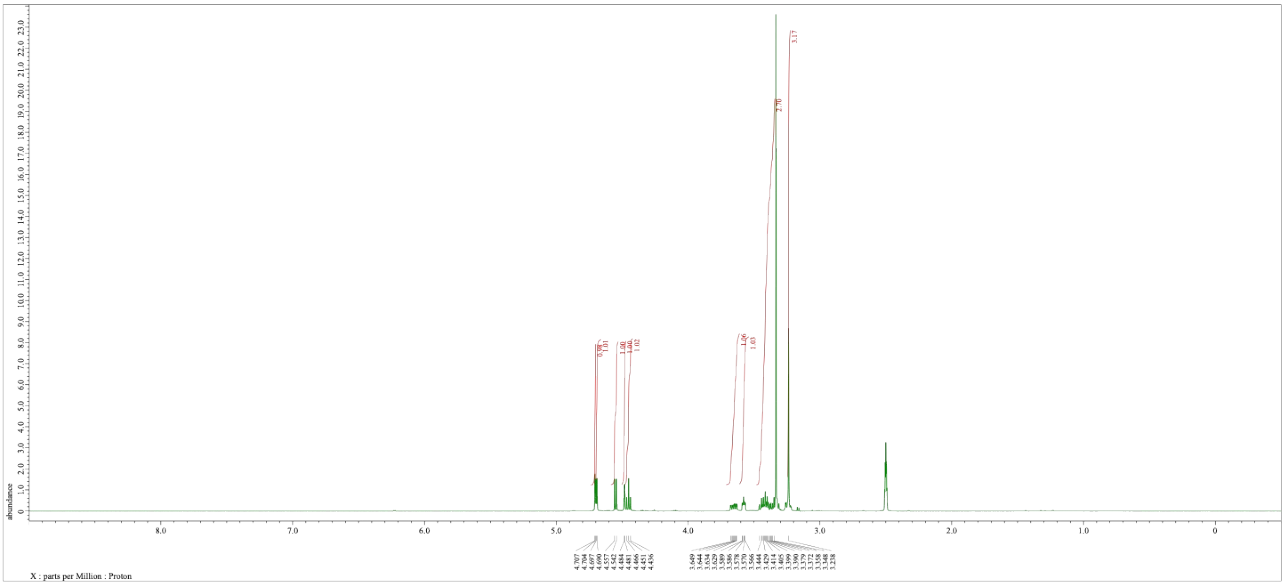

Compound **1** (^{13}C-NMR, DMSO-D6, 101 MHz)

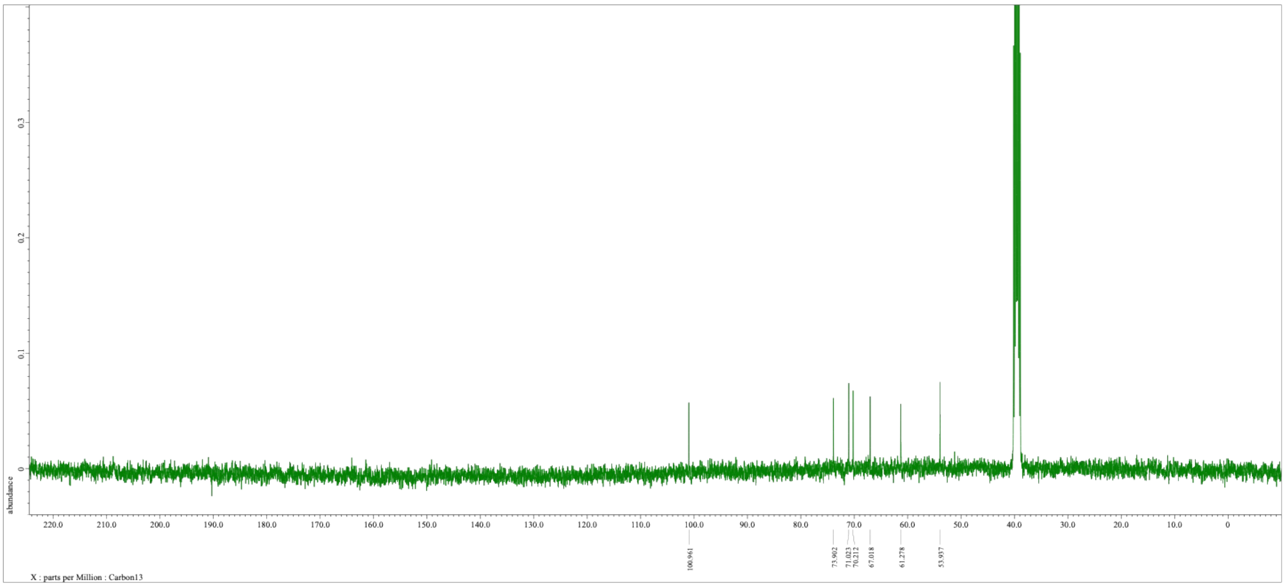

Compound **2** (^{1}H-NMR, $CDCl_3$, 400 MHz)

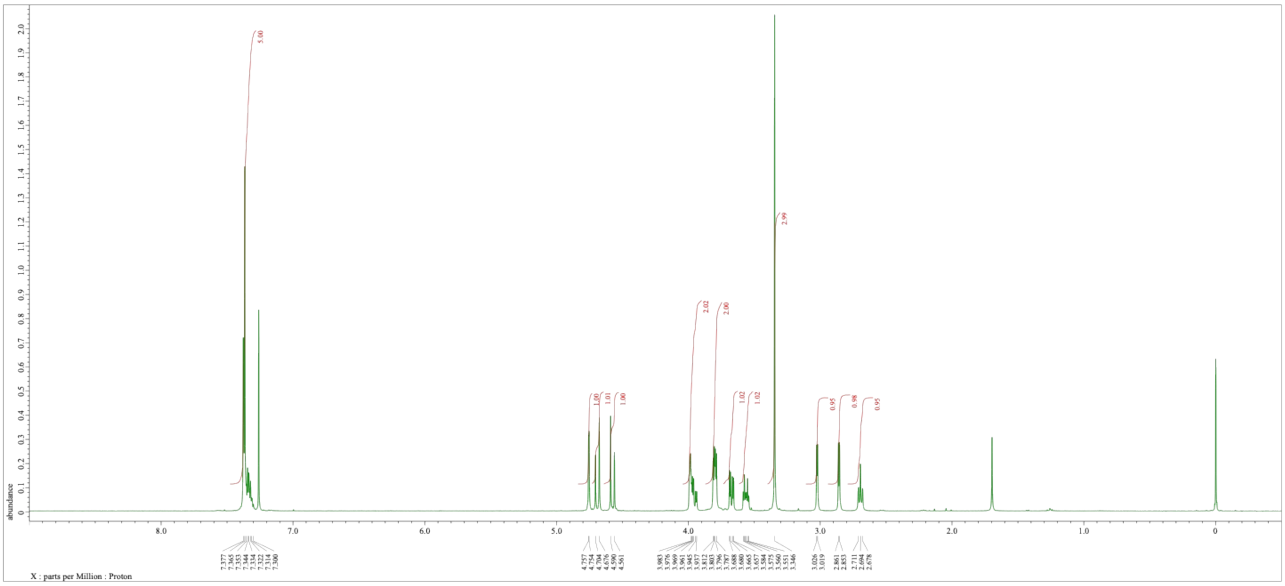

Compound **2** (^{13}C-NMR, $CDCl_3$, 101 MHz)

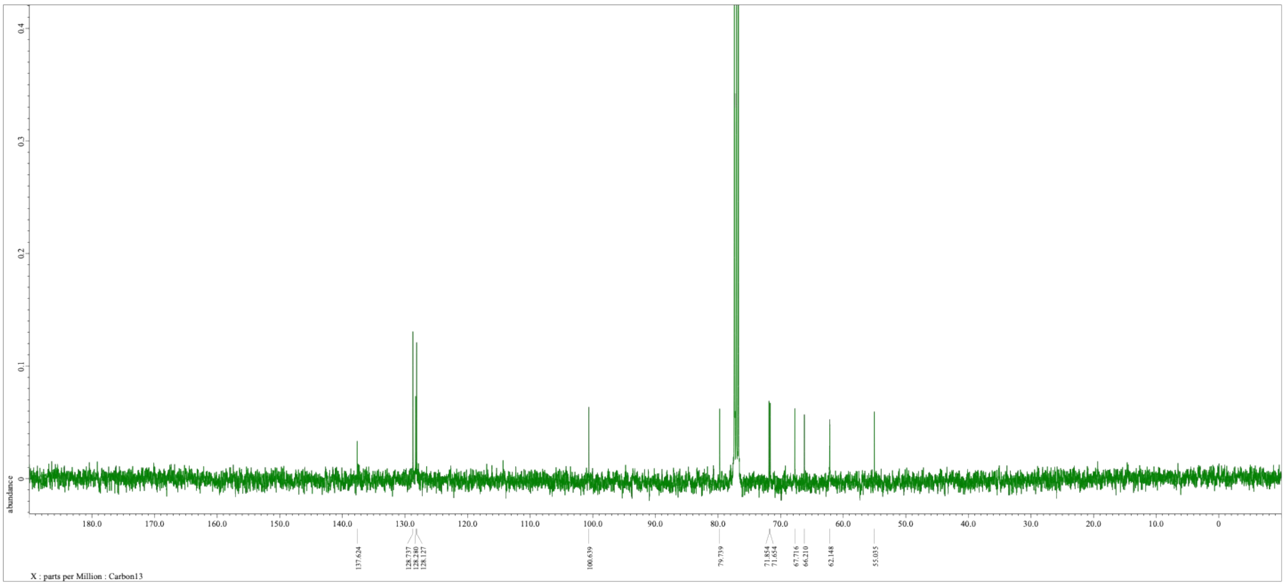

Compound **3** (^{1}H-NMR, $CDCl_3$, 400 MHz)

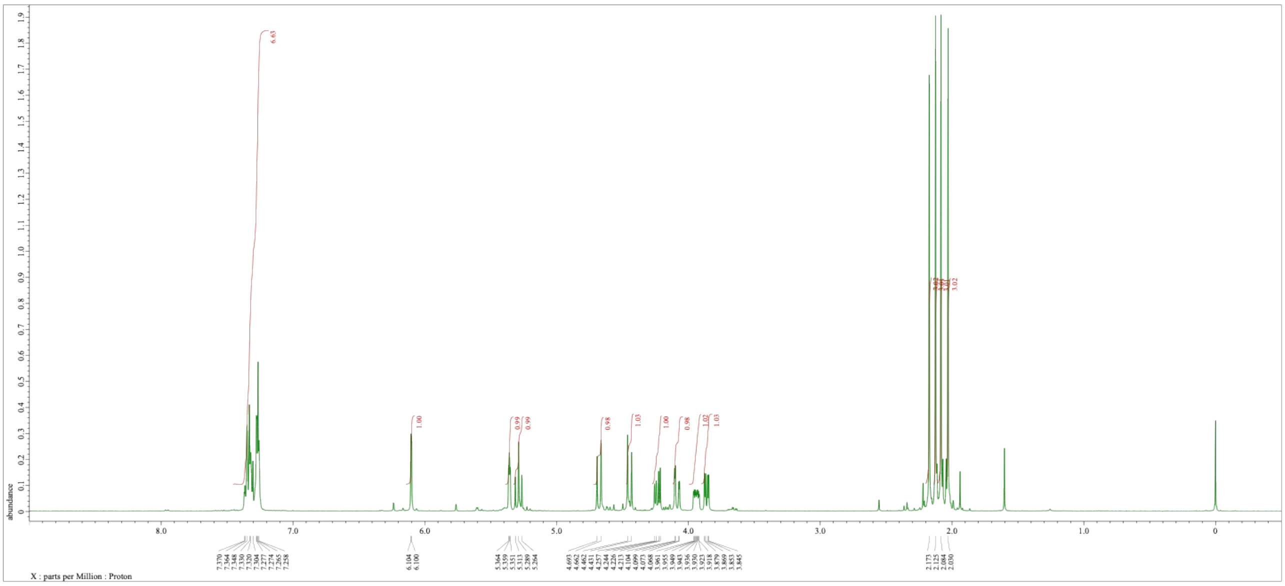

Compound **3** (^{13}C-NMR, $CDCl_3$, 101 MHz)

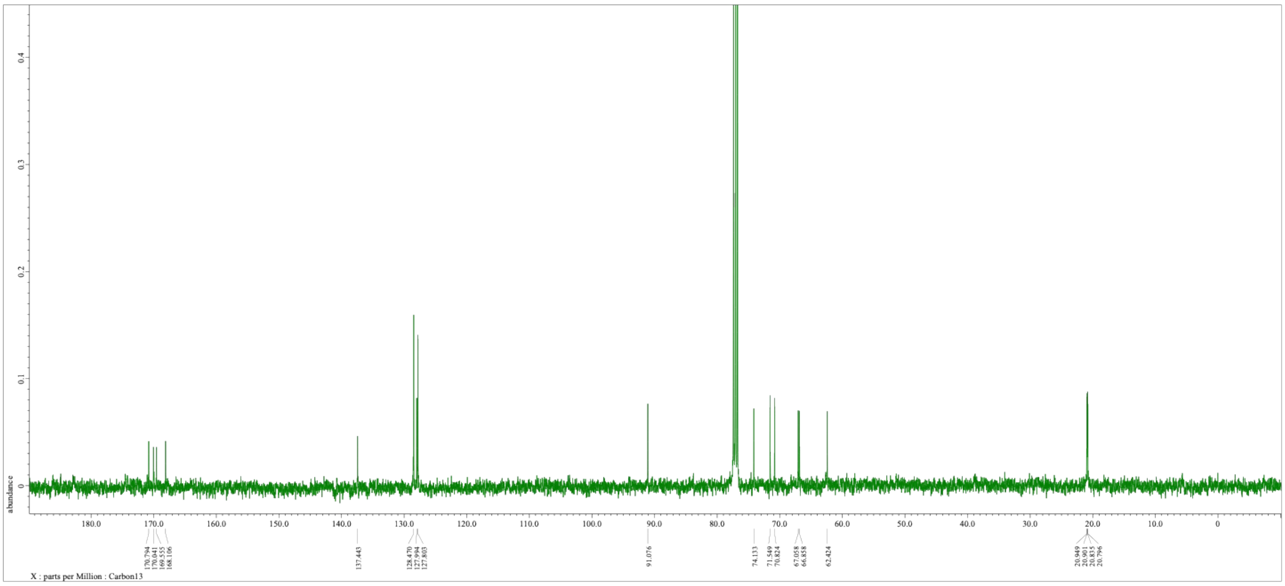

Compound **4** (^{1}H-NMR, $CDCl_3$, 400 MHz)

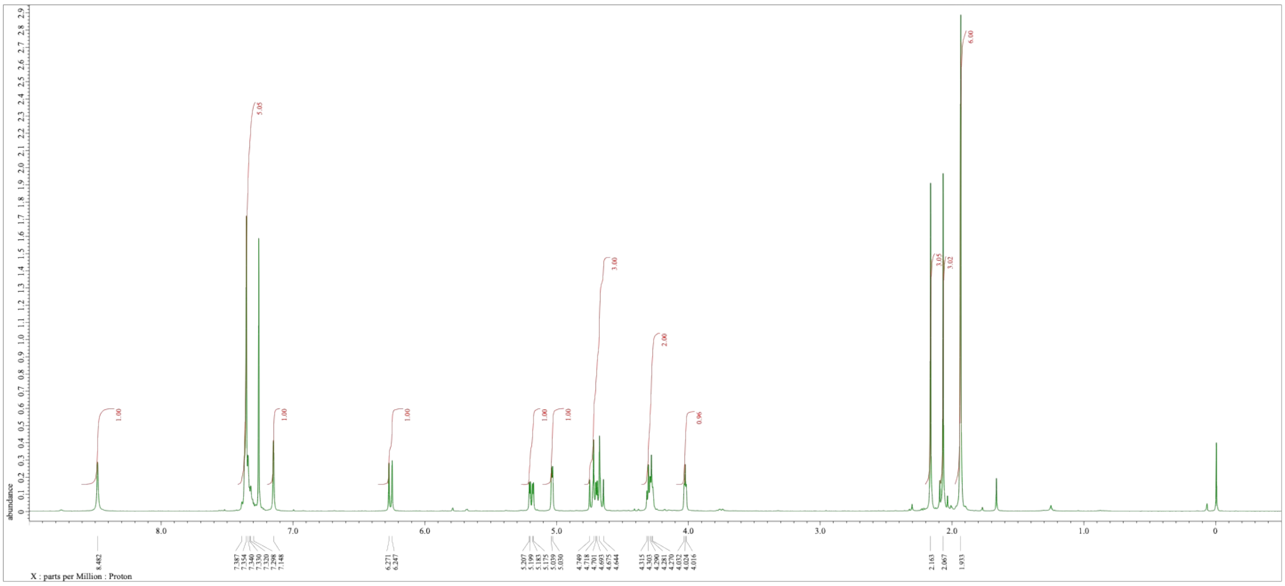

Compound **4** (^{13}C-NMR, $CDCl_3$, 101 MHz)

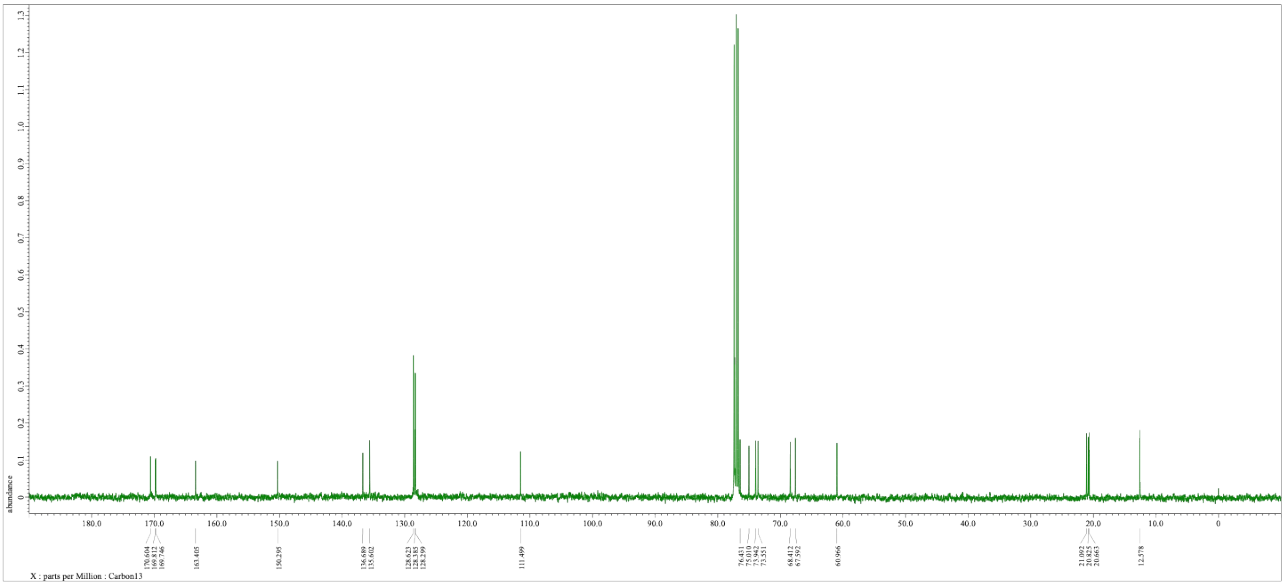

Compound **5** (^{1}H-NMR, DMSO-D6, 400 MHz)

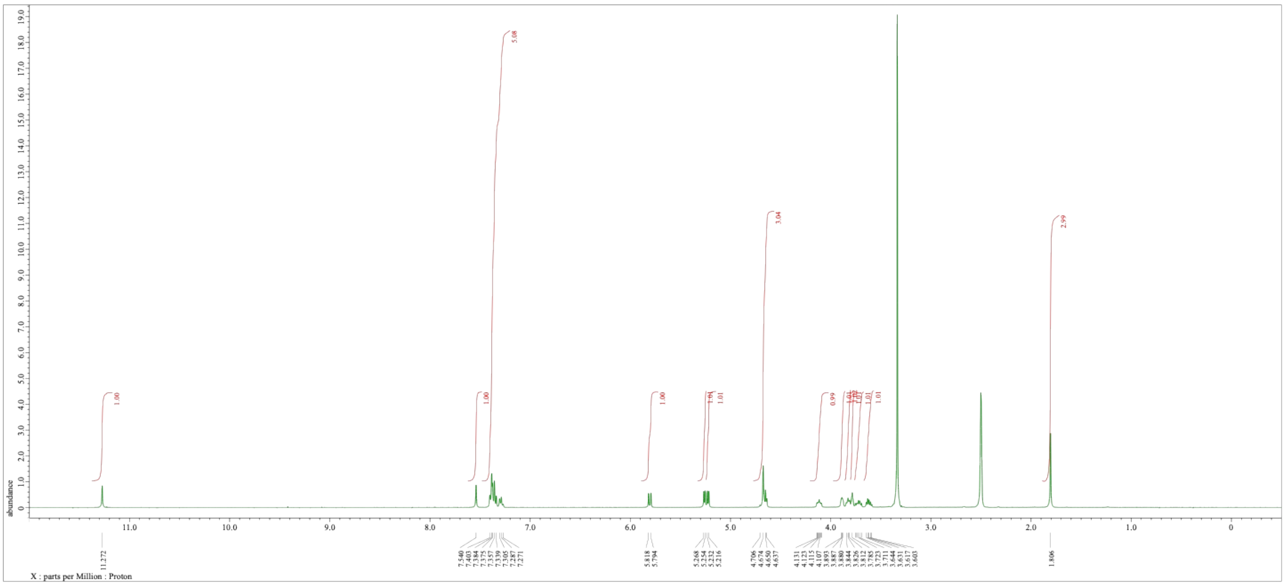

Compound **5** (^{13}C-NMR, DMSO-D6, 101 MHz)

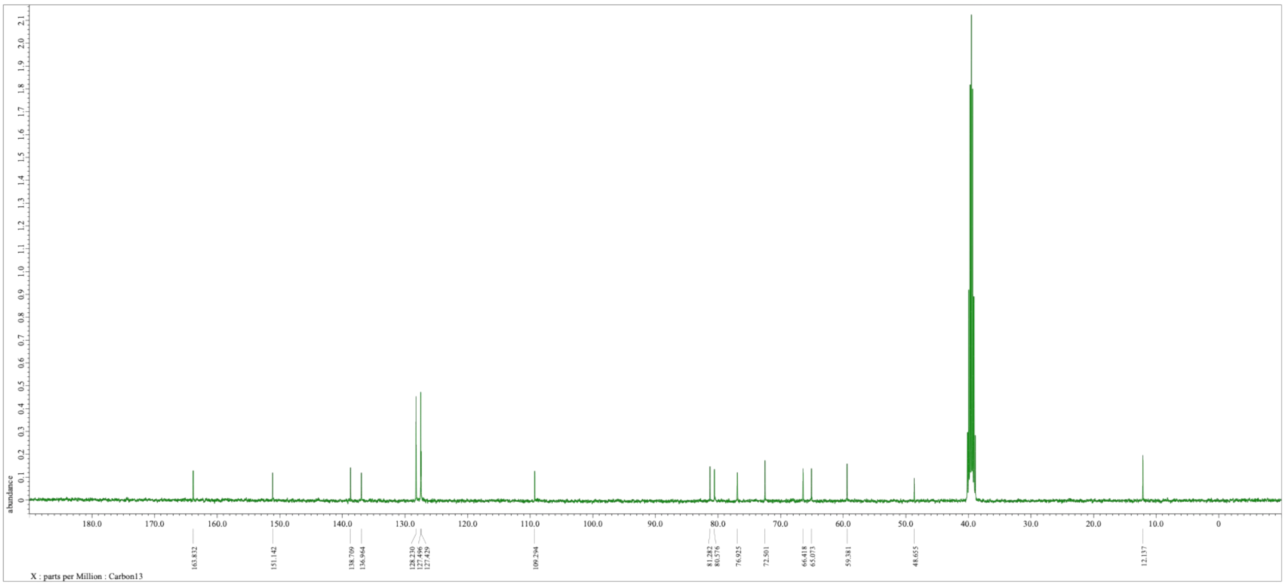

Compound **6** (^{1}H-NMR, $CDCl_3$, 400 MHz)

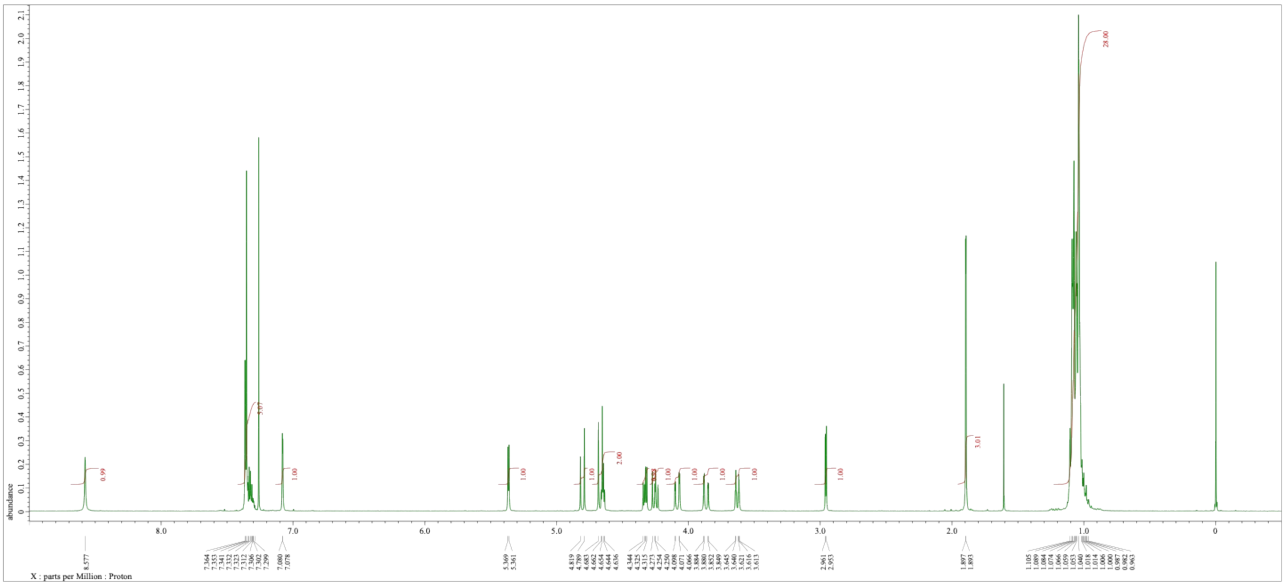

Compound **6** (^{13}C-NMR, $CDCl_3$, 101 MHz)

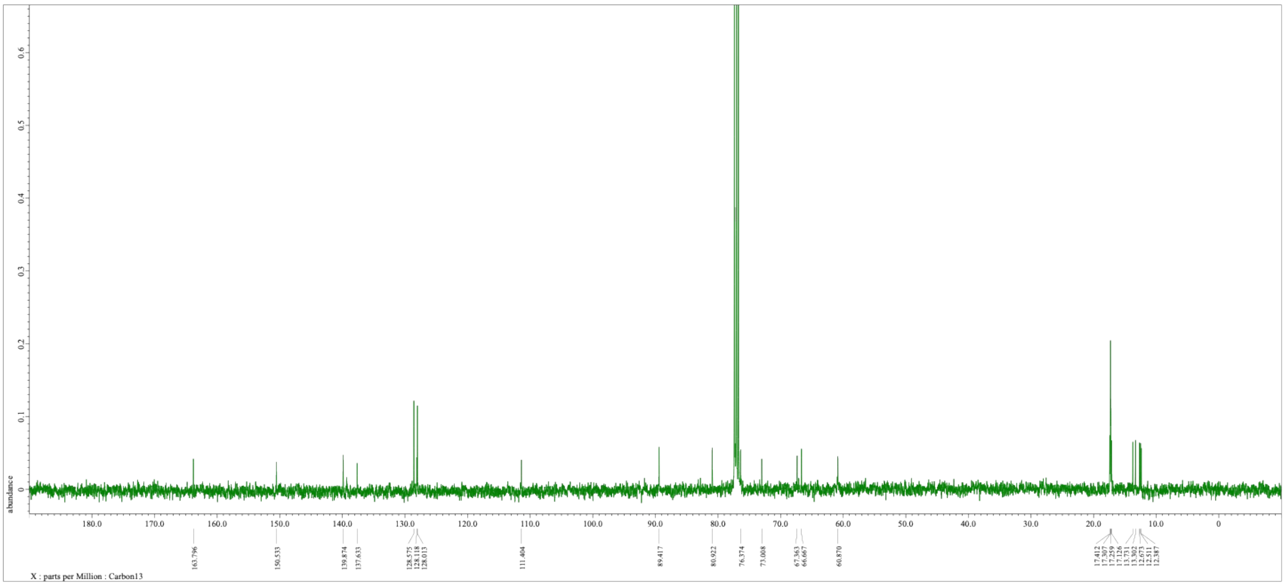

Compound **7** (^{1}H-NMR, $CDCl_3$, 400 MHz)

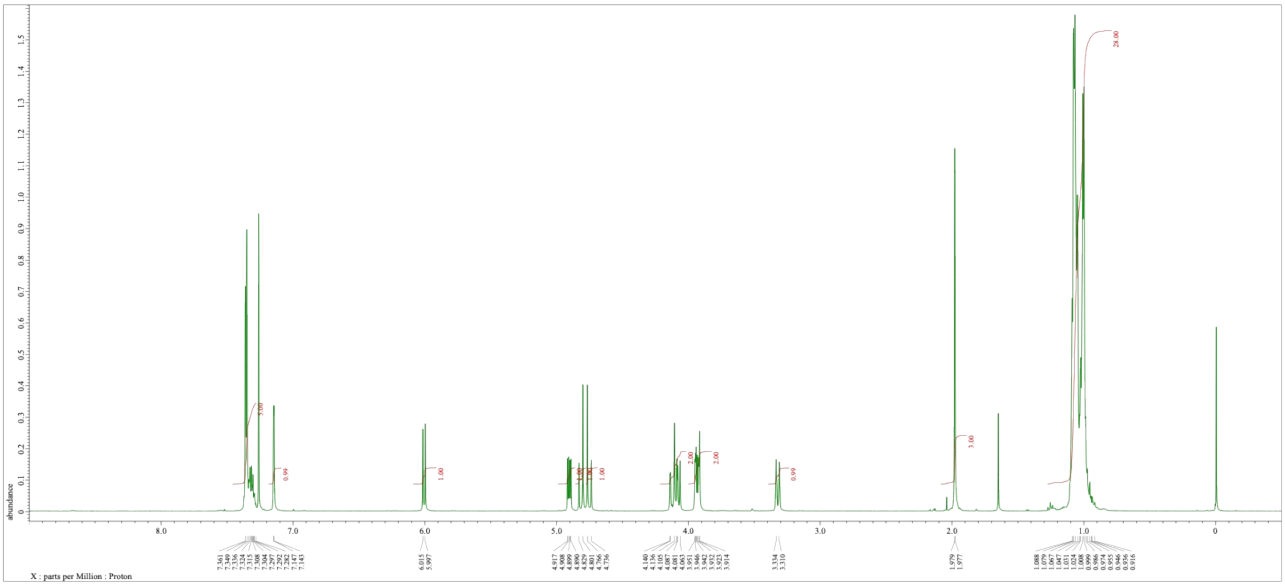

Compound **7** (^{13}C-NMR, $CDCl_3$, 101 MHz)

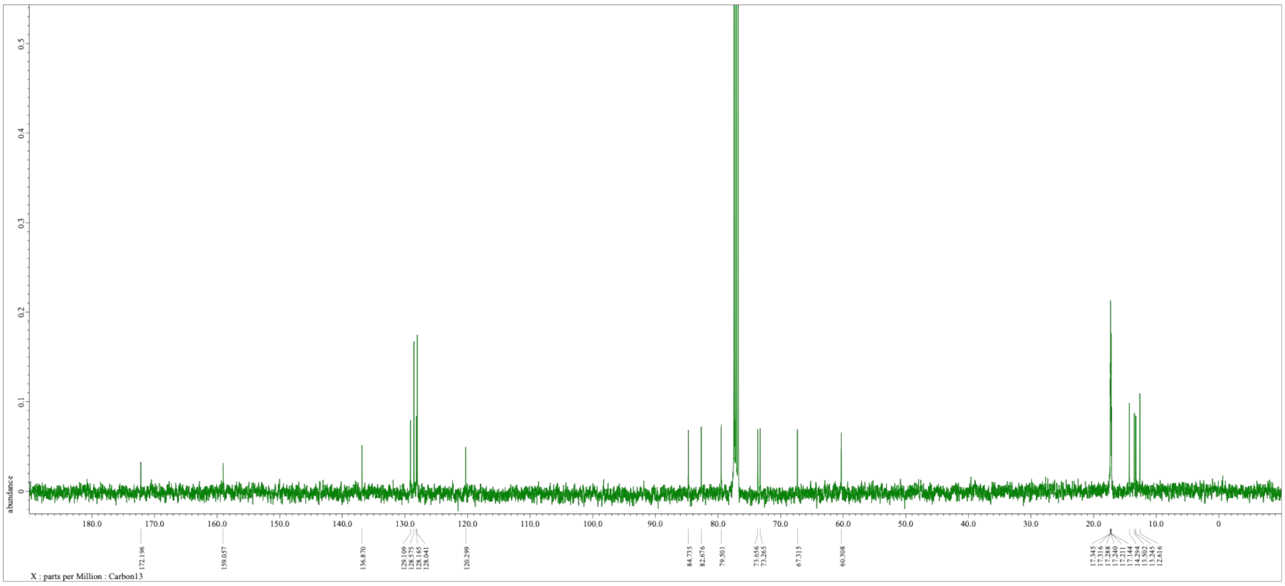

Compound **8** (^{1}H-NMR, MeOD-D4, 400 MHz)

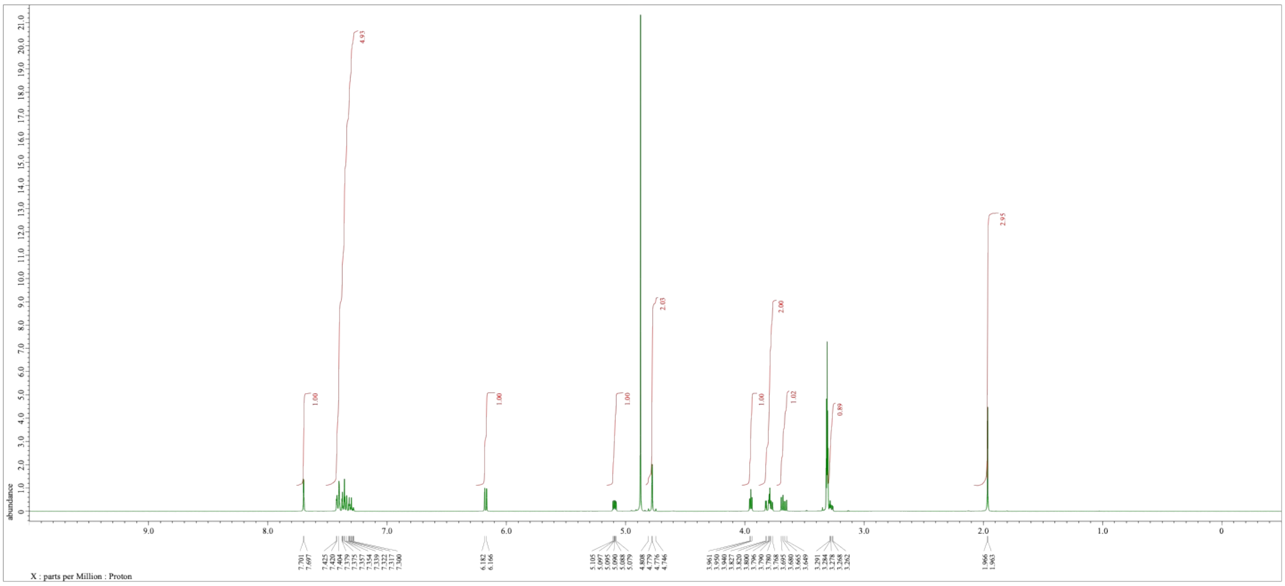

Compound **8** (^{13}C-NMR, MeOD-D4, 101 MHz)

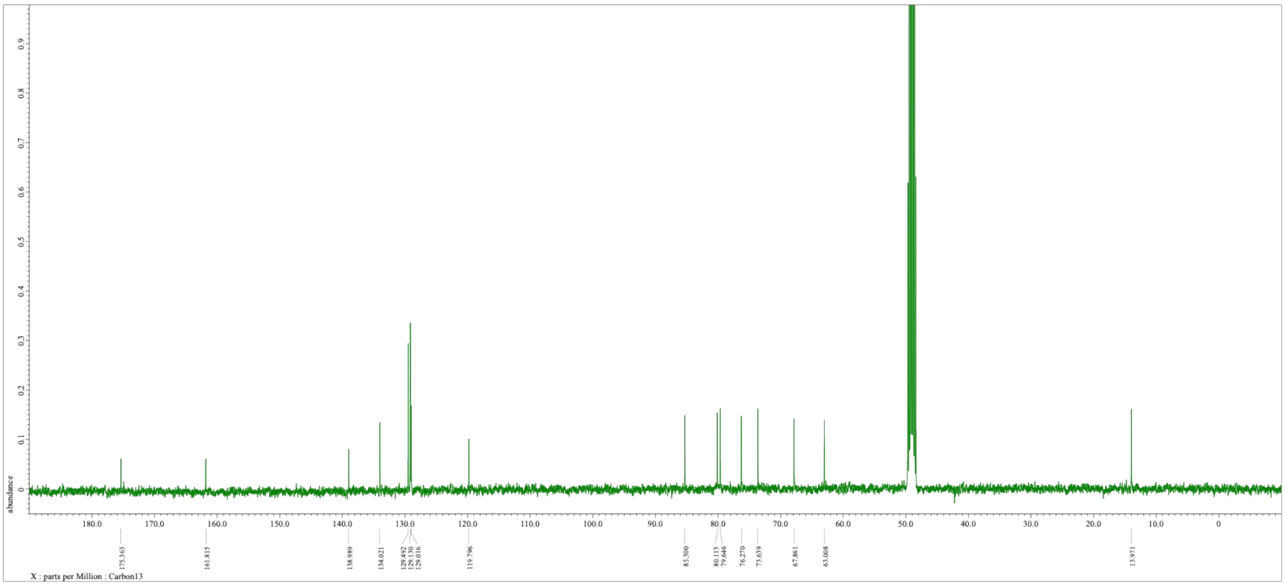

Compound **9** (^{1}H-NMR, $CDCl_3$, 400 MHz)

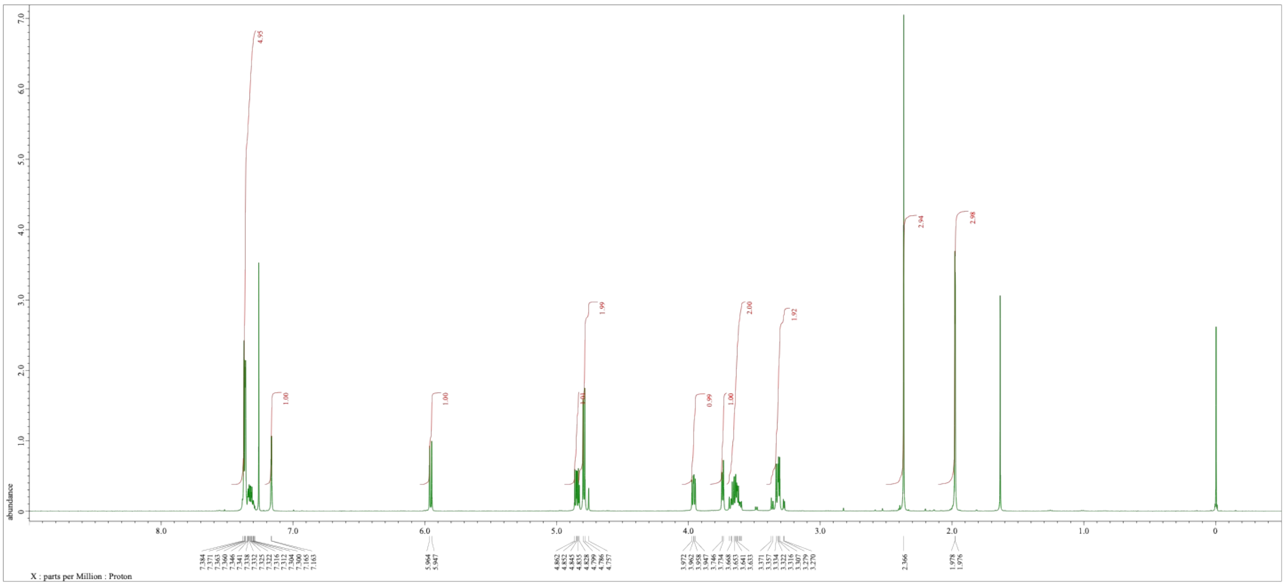

Compound **9** (^{13}C-NMR, $CDCl_3$, 101 MHz)

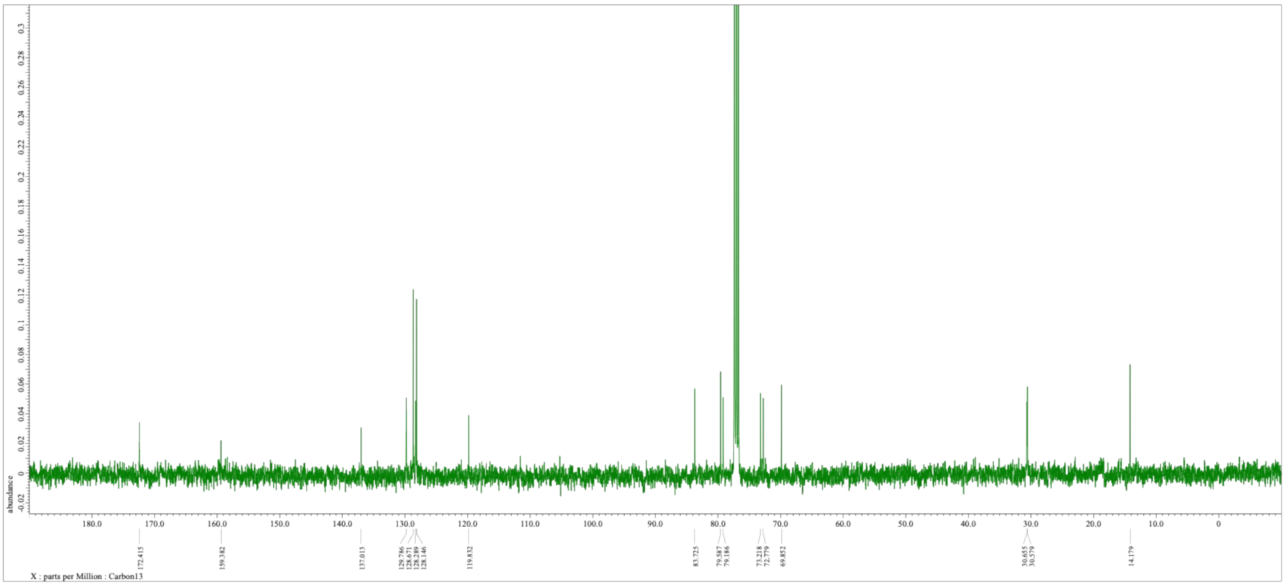

Compound **10** (^{1}H-NMR, $CDCl_3$, 400 MHz)

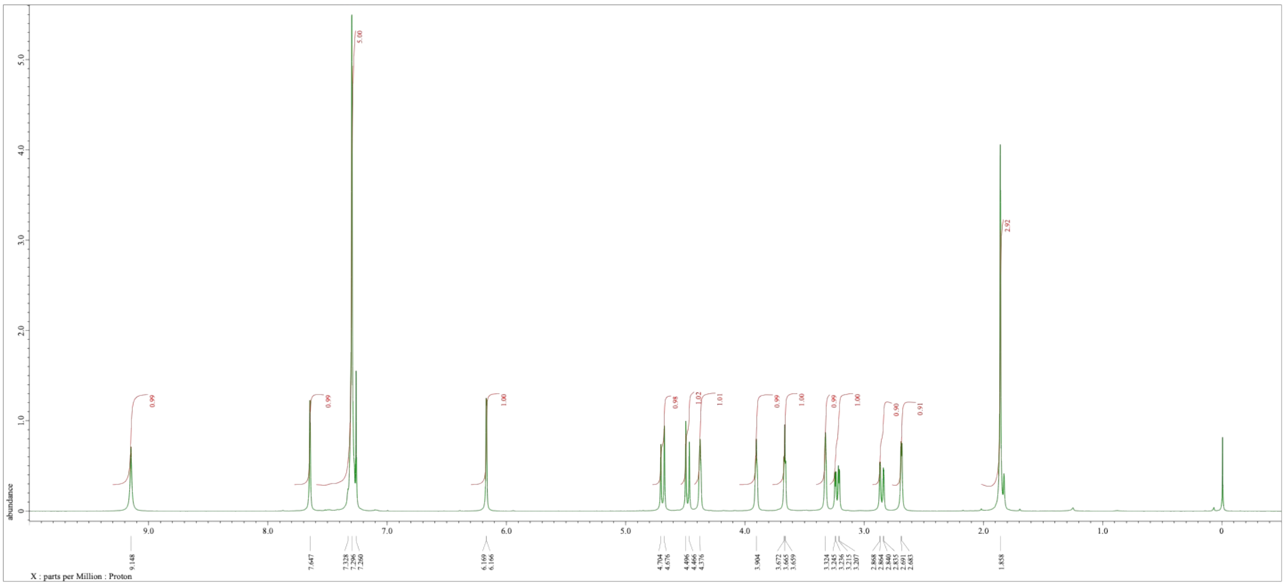

Compound **10** (^{13}C-NMR, $CDCl_3$, 101 MHz)

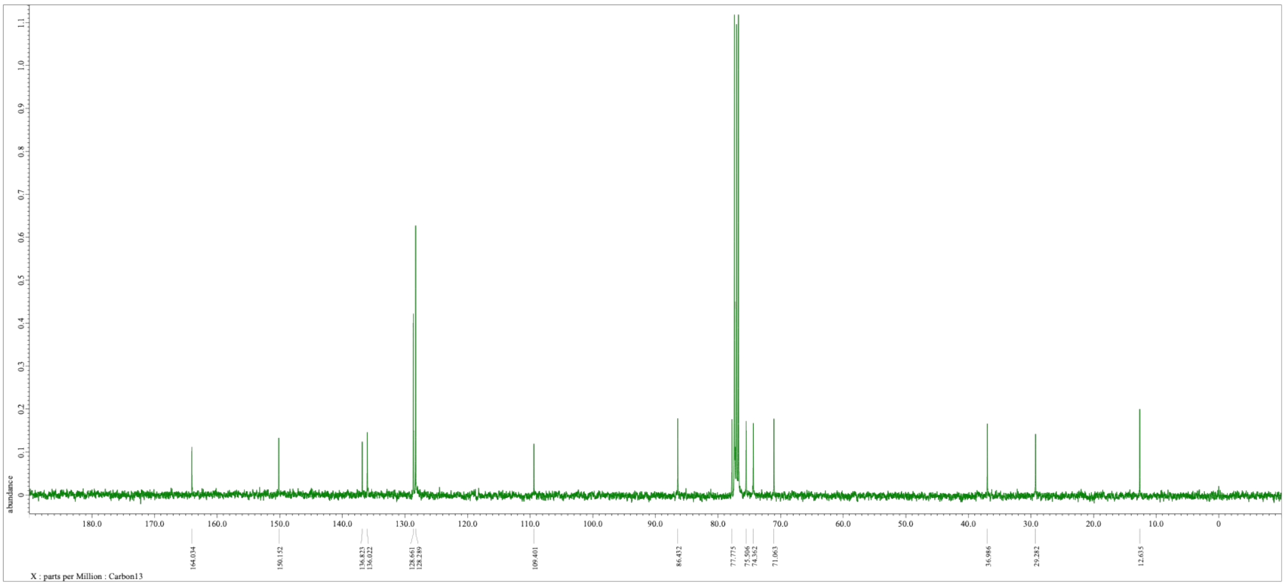

Compound **11** (^{1}H-NMR, $CDCl_3$, 400 MHz)

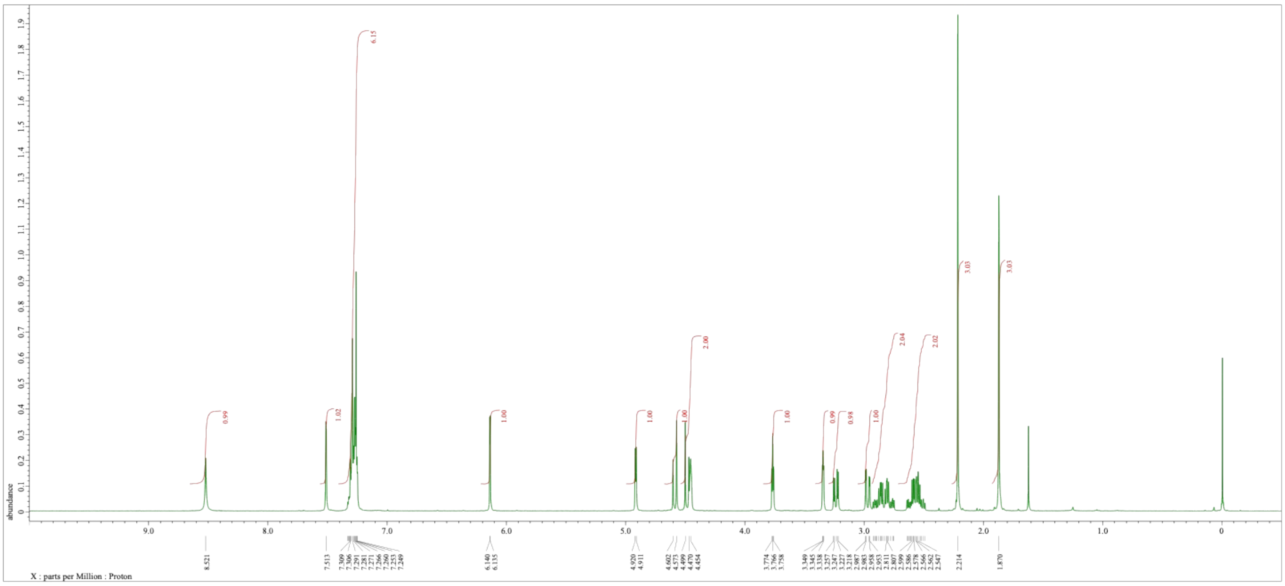

Compound **11** (^{13}C-NMR, $CDCl_3$, 101 MHz)

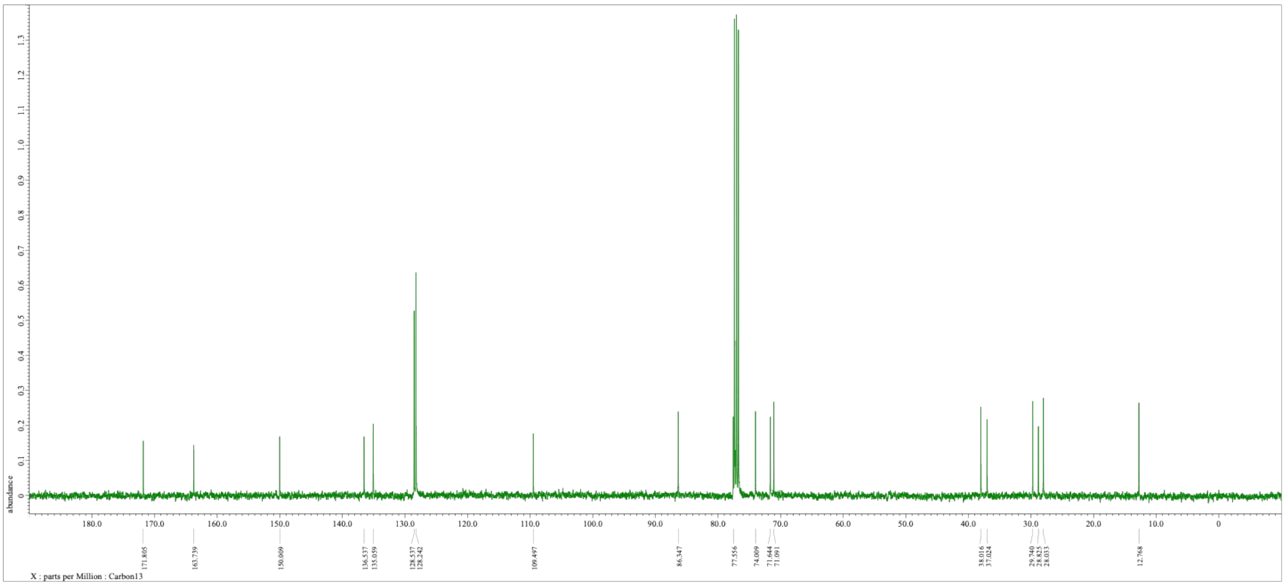

Compound **12** (^{1}H-NMR, $CDCl_3$, 400 MHz)

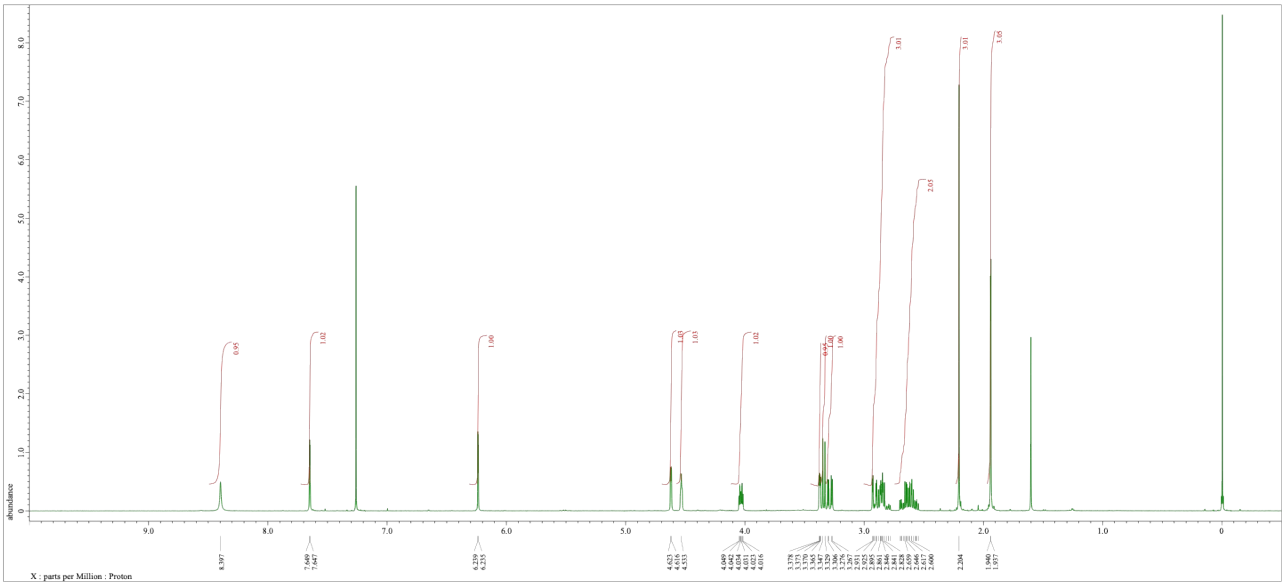

Compound **12** (^{13}C-NMR, $CDCl_3$, 101 MHz)

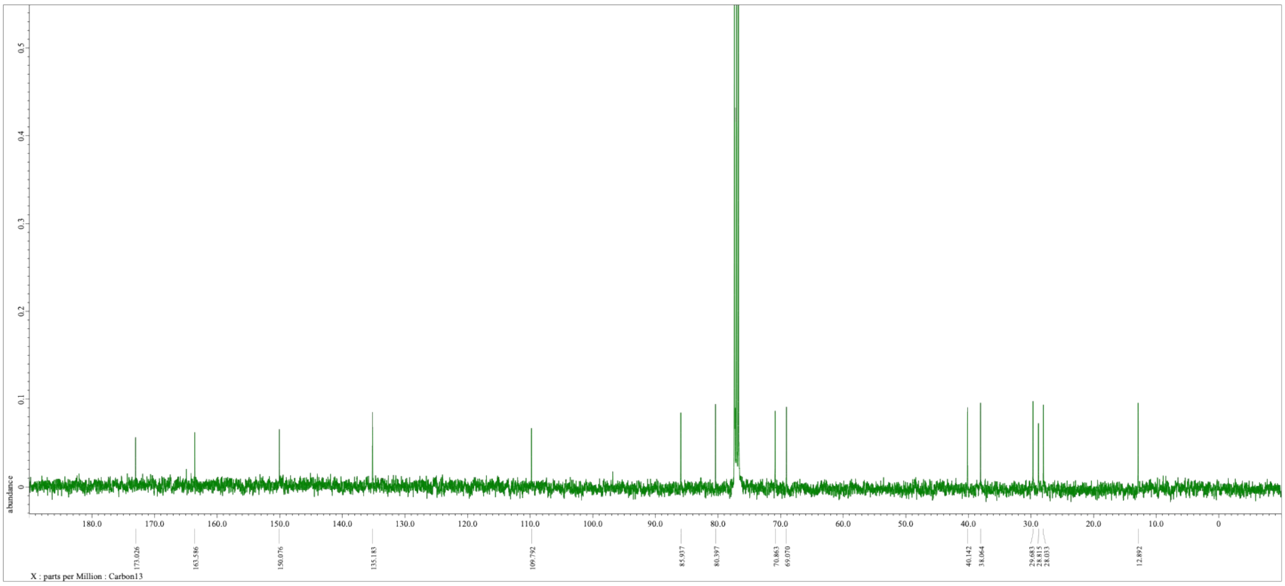

Compound **13** (^{31}P-NMR, $CDCl_3$, 162 MHz)

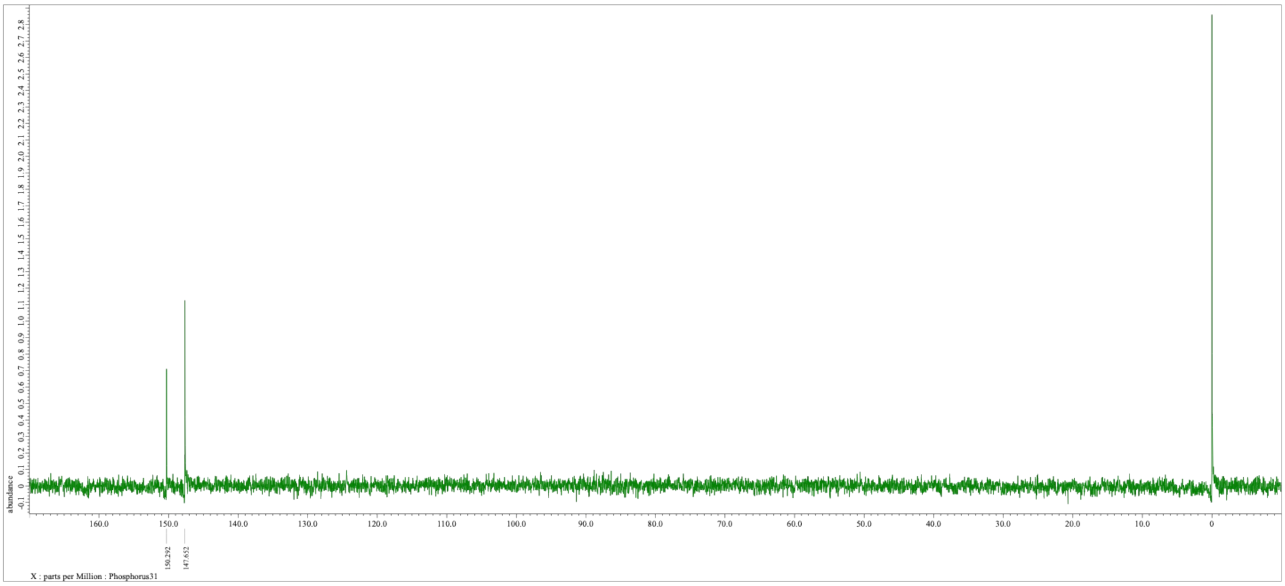

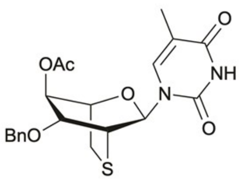

S25

Compound **S25** (^{1}H-NMR, $CDCl_3$, 400 MHz)

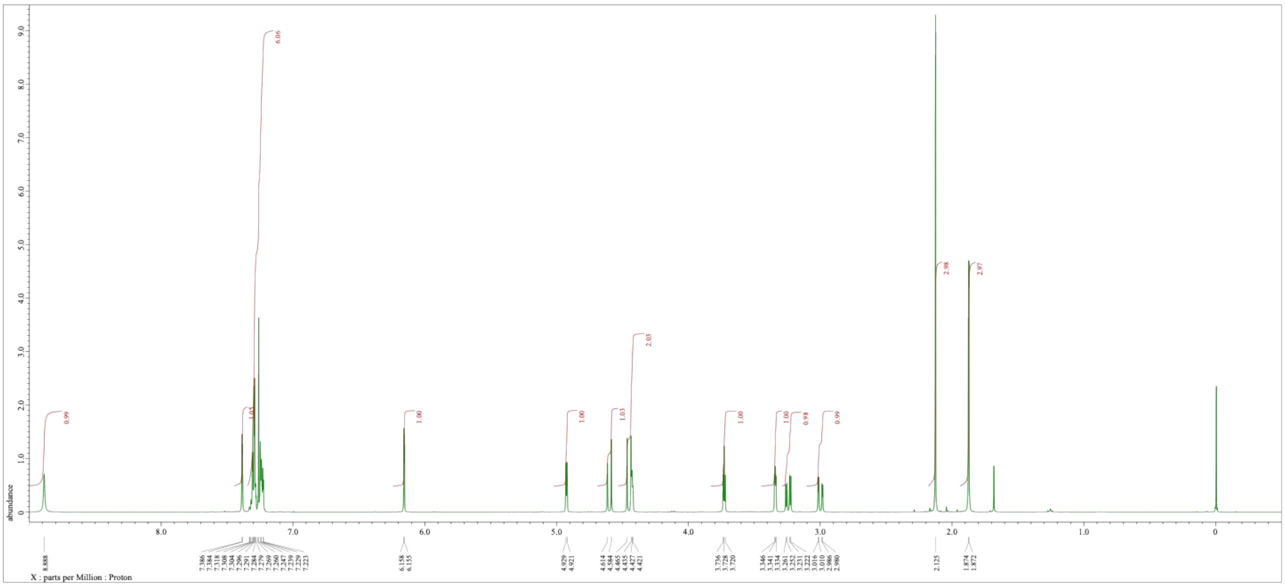

Compound **S25** (^{13}C-NMR, $CDCl_3$, 101 MHz)

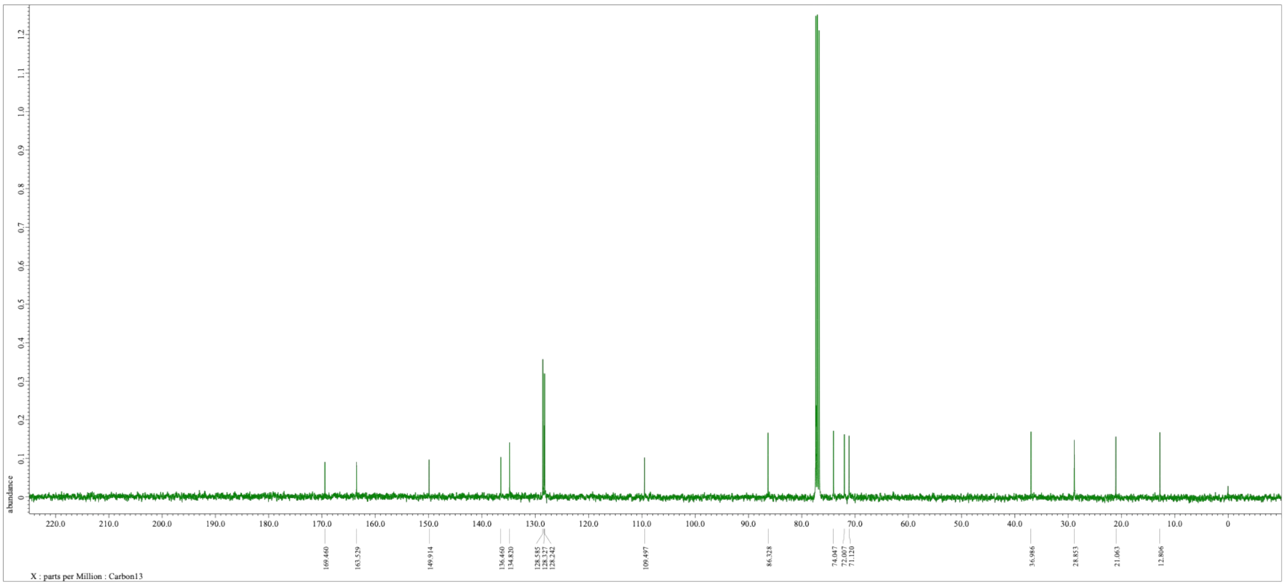

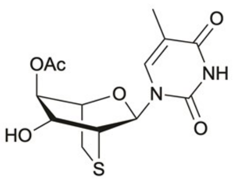

S26

Compound **S26** (^{1}H-NMR, $CDCl_3$, 400 MHz)

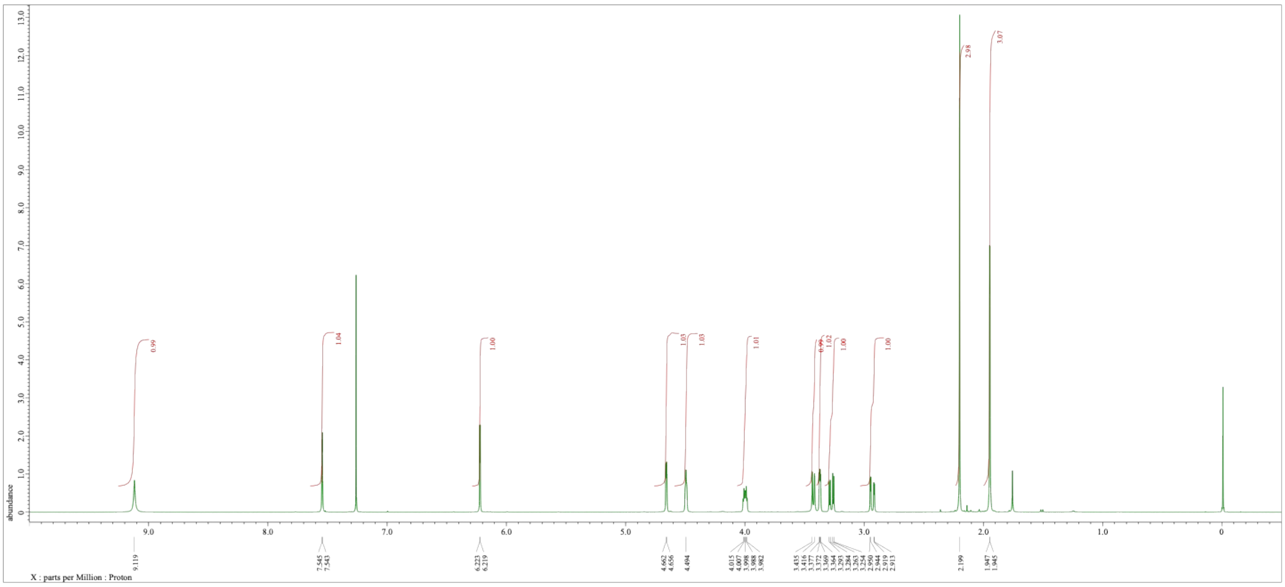

Compound **S26** (^{13}C-NMR, $CDCl_3$, 101 MHz)

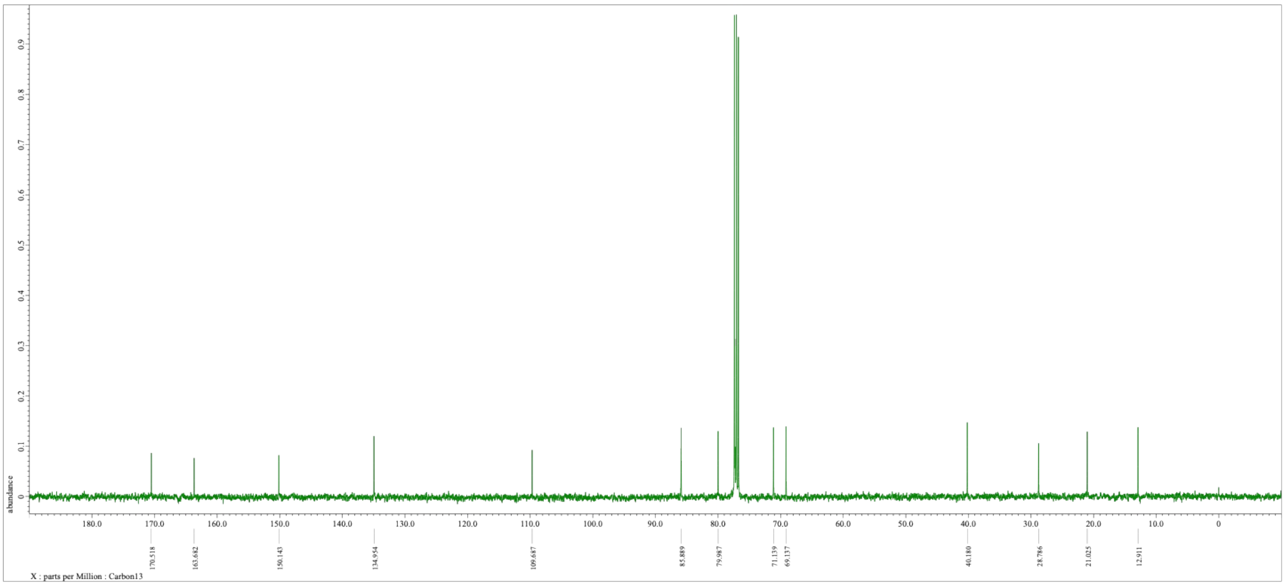

Compound **14** (^{1}H-NMR, $CDCl_3$, 400 MHz)

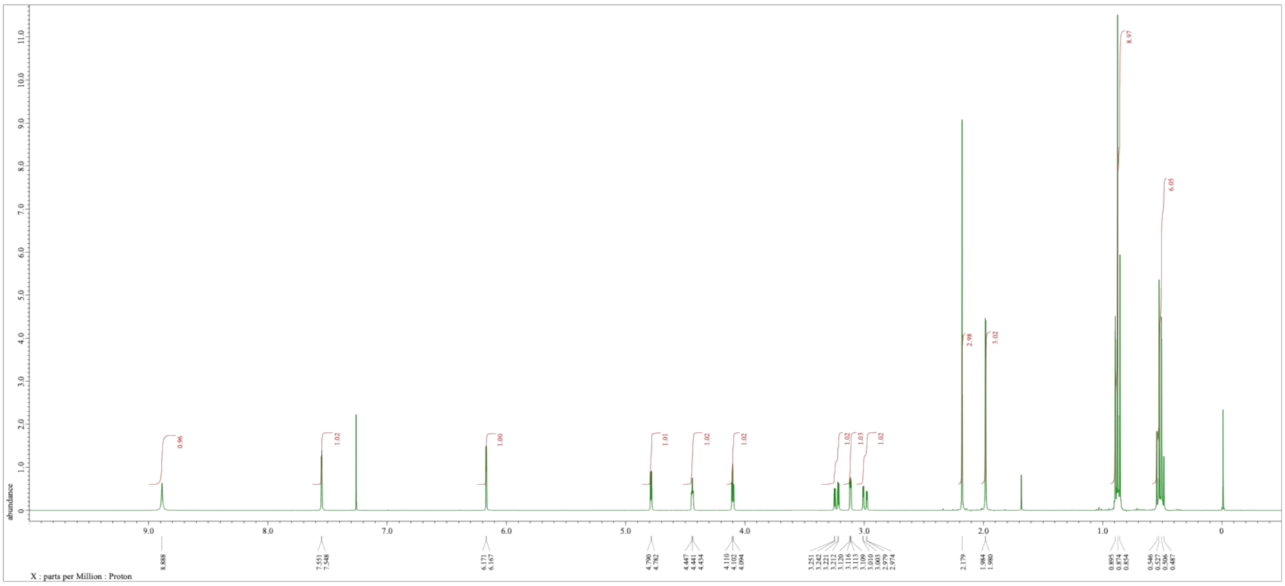

Compound **14** (^{13}C-NMR, $CDCl_3$, 101 MHz)

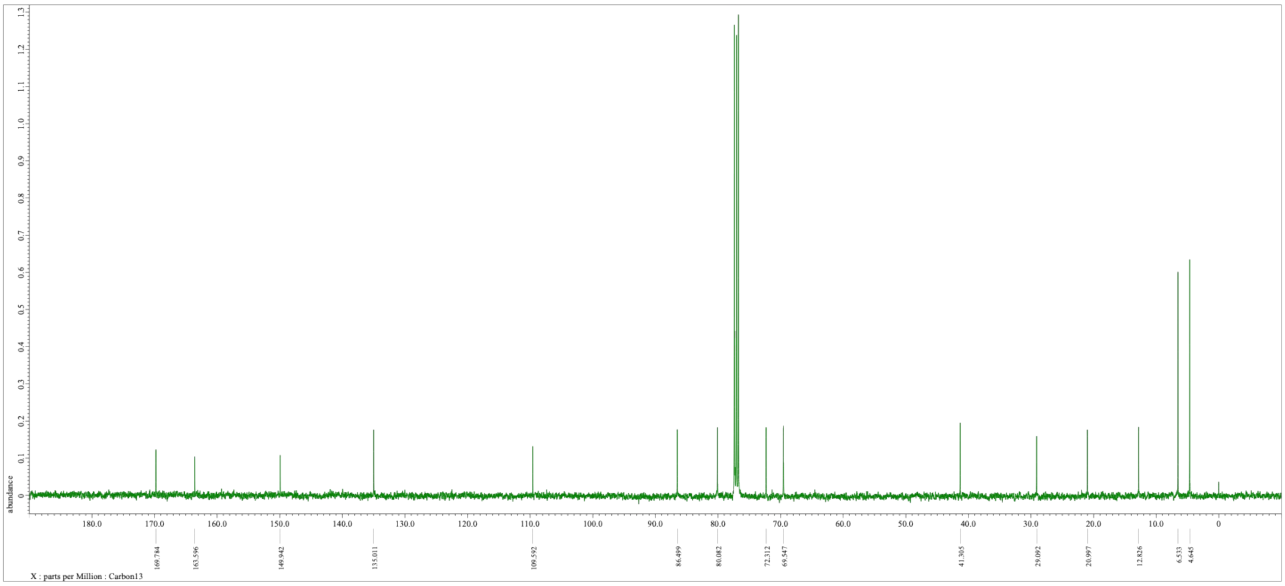

Compound **15** (^{1}H-NMR, $CDCl_3$, 400 MHz)

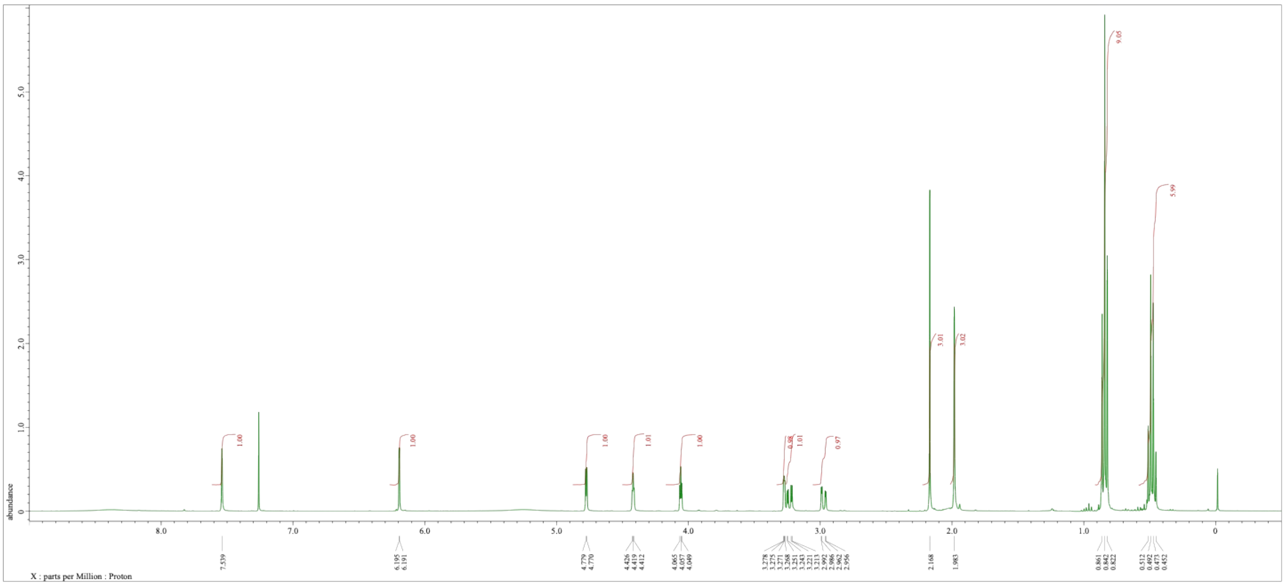

Compound **15** (^{13}C-NMR, $CDCl_3$ 101 MHz)

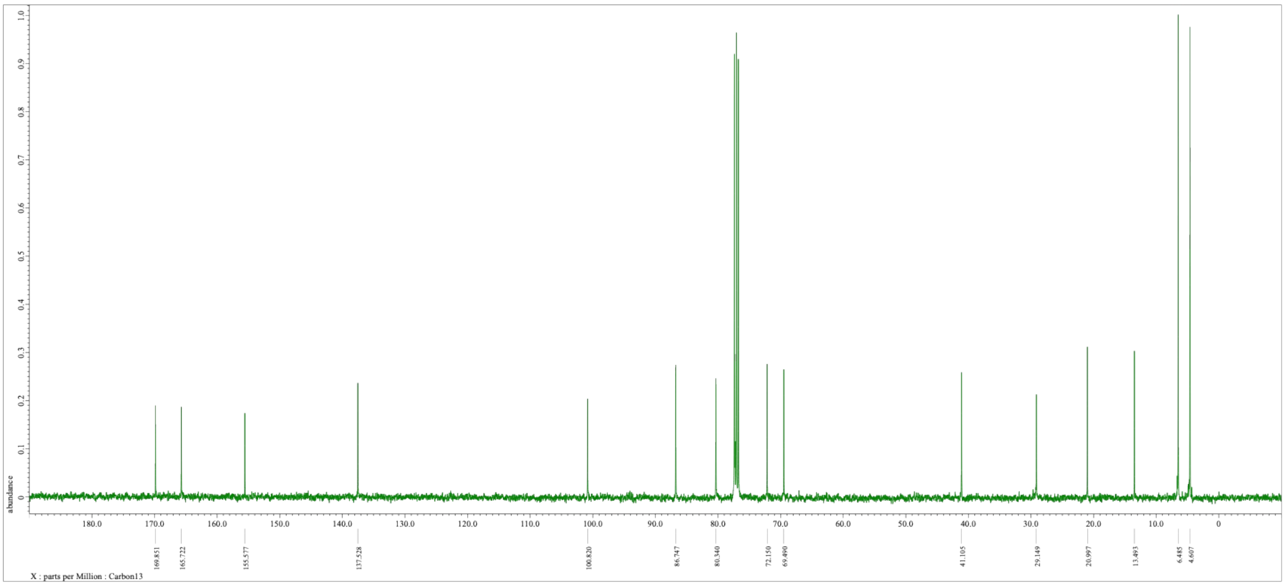

Compound **16** (^{1}H-NMR, $CDCl_3$, 400 MHz)

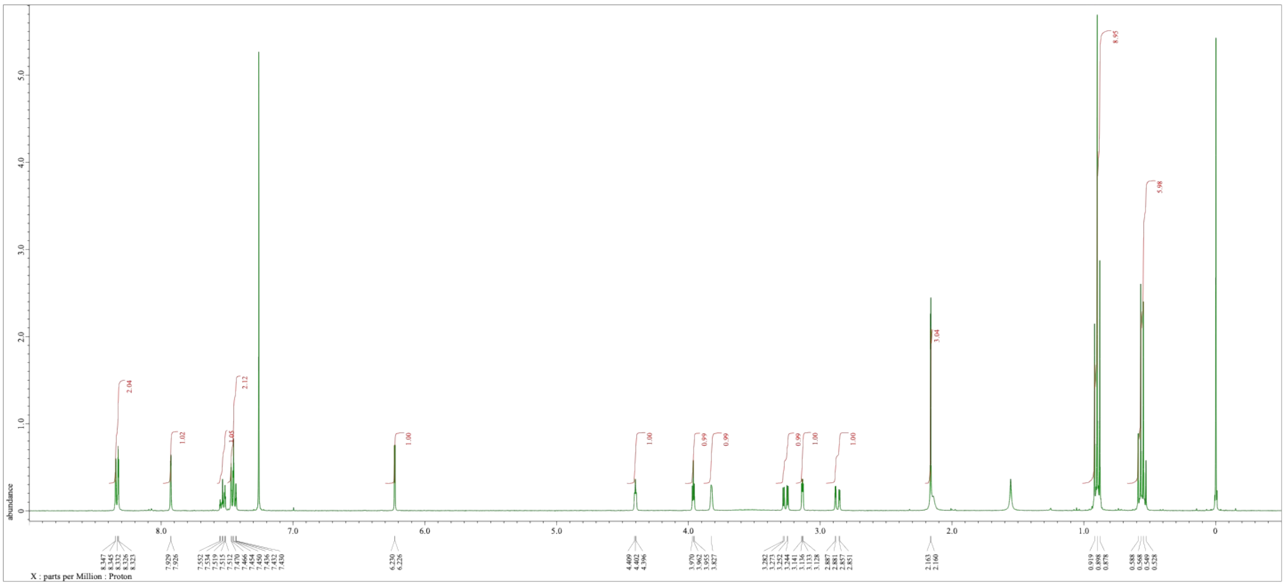

Compound **16** (^{13}C-NMR, $CDCl_3$, 101 MHz)

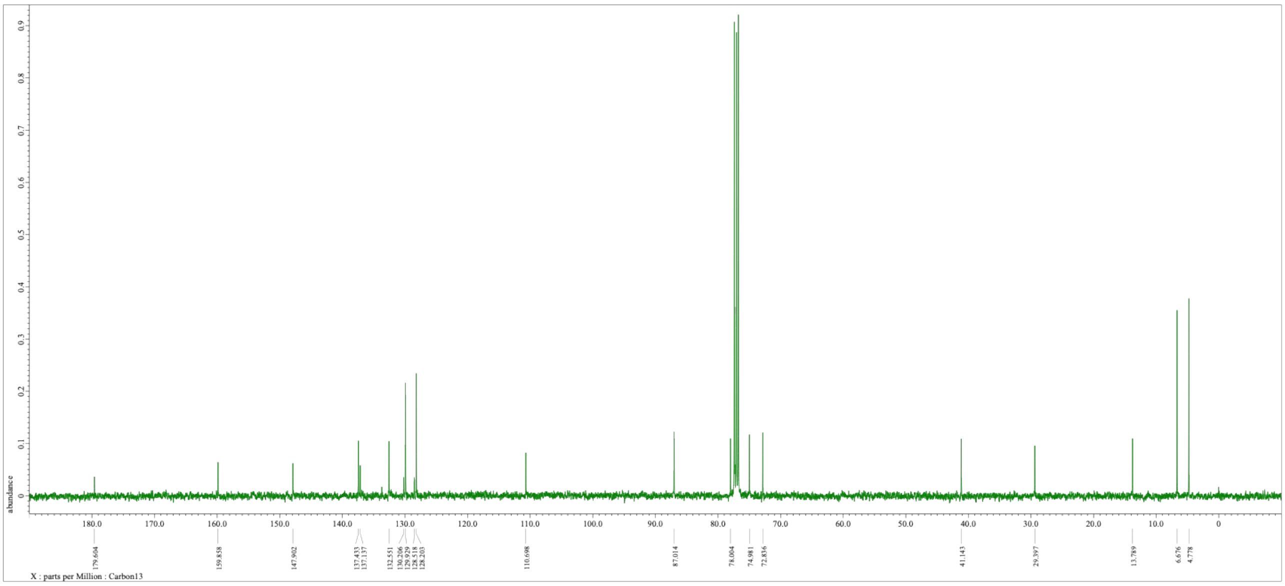

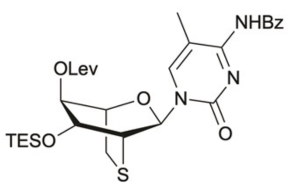

S27

Compound **S27** (^{1}H-NMR, $CDCl_3$, 400 MHz)

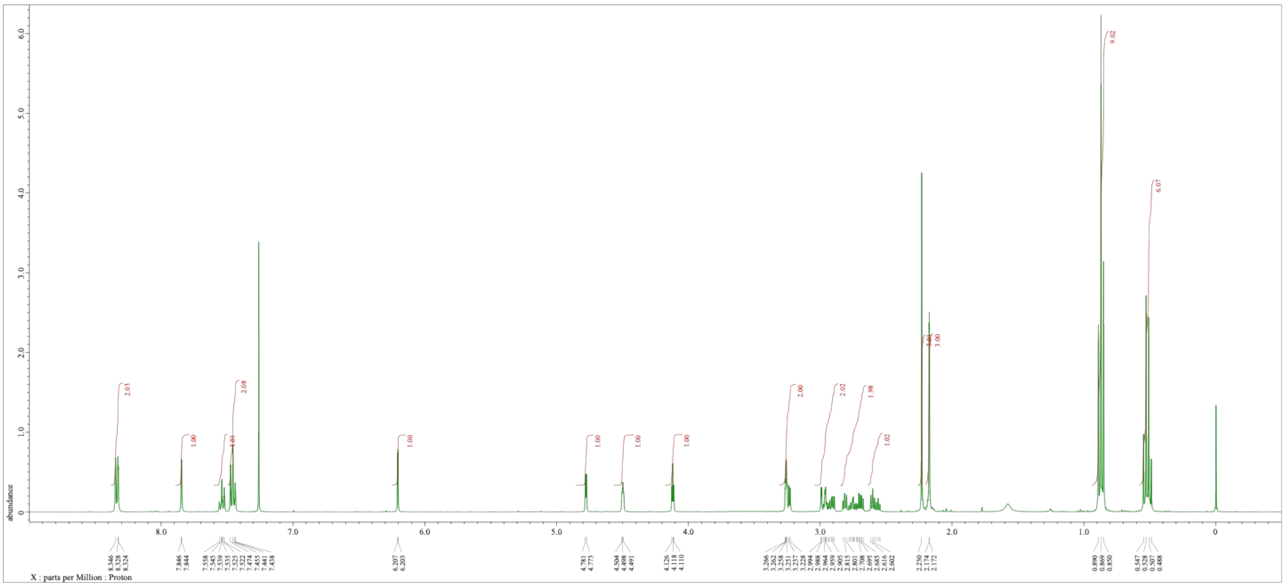

Compound **S27** (^{13}C-NMR, $CDCl_3$, 101 MHz)

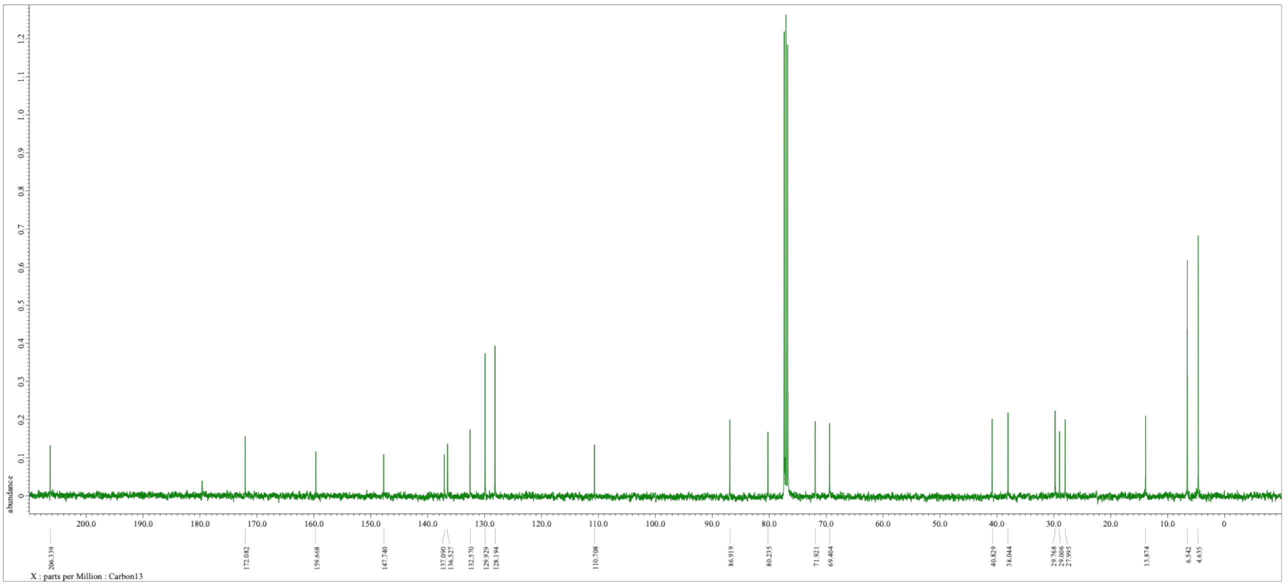

Compound **17** (^{1}H-NMR, $CDCl_3$, 400 MHz)

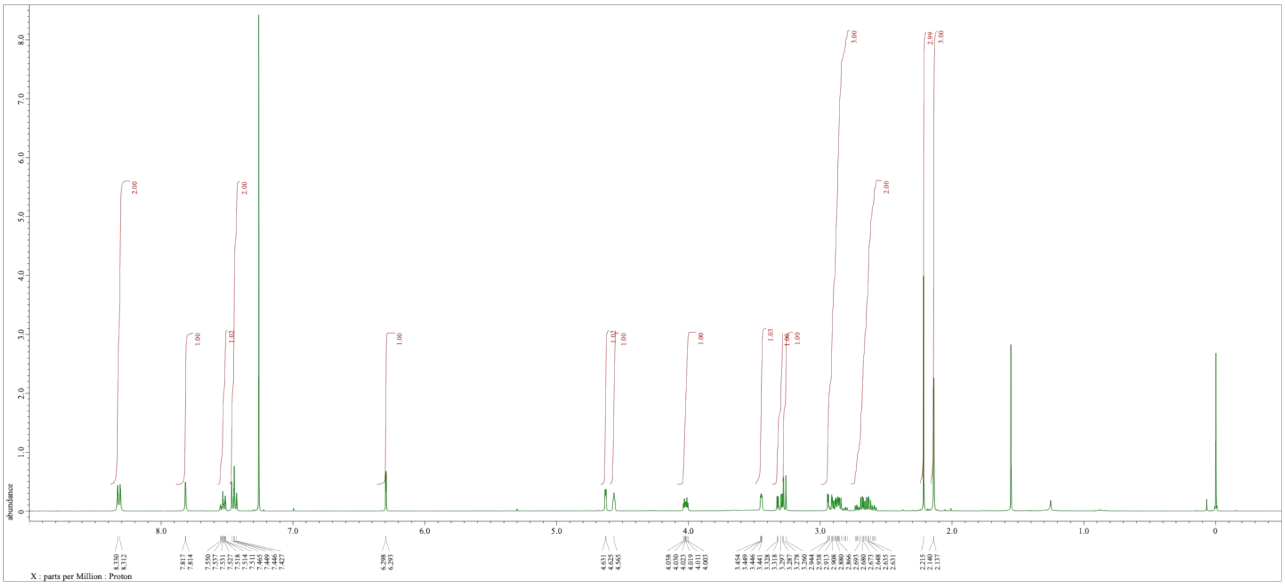

Compound **17** (^{13}C-NMR, $CDCl_3$, 101 MHz)

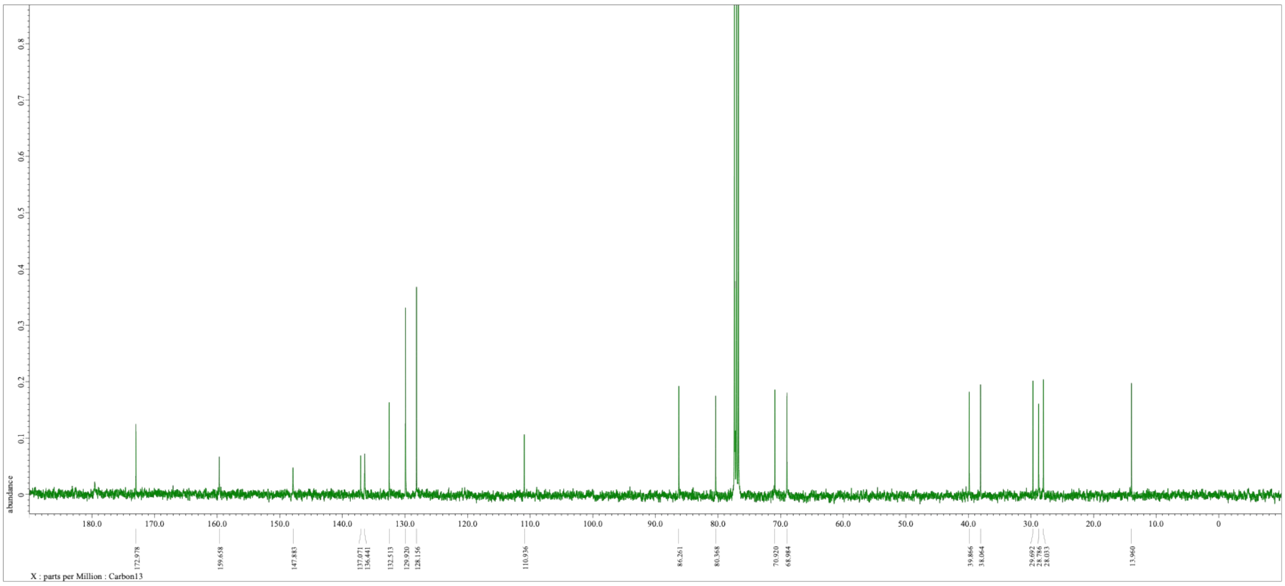

Compound **18** (^{31}P-NMR, $CDCl_3$, 162 MHz)

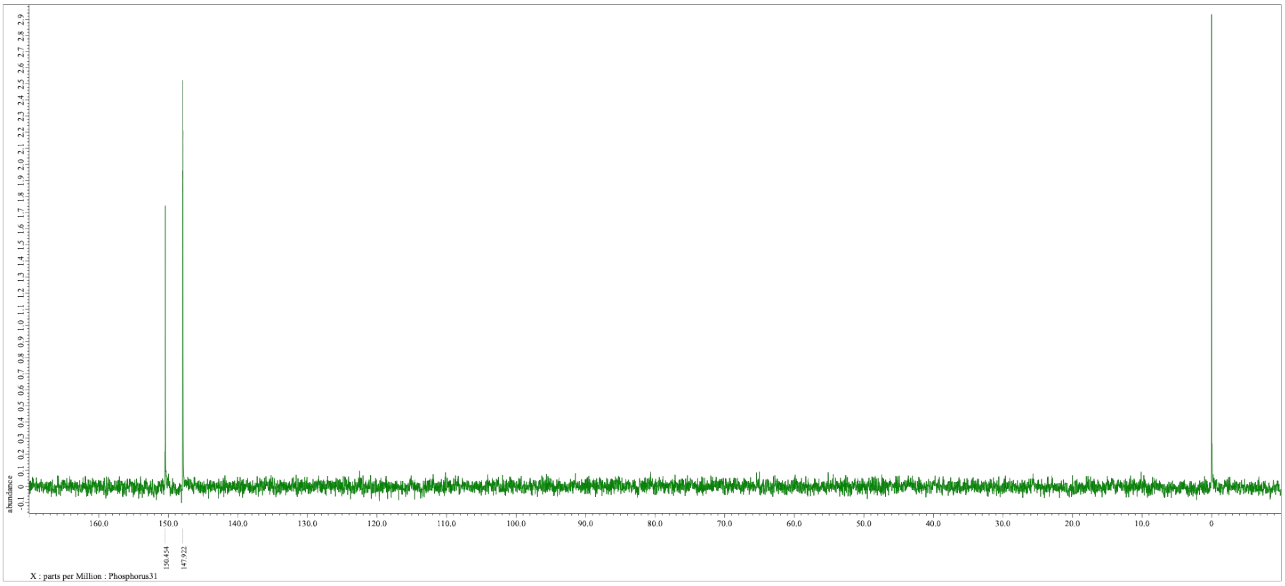

3. Characterization data (HPLC and mass data) of synthesized oligonucleotide

HPLC (**ON1**)

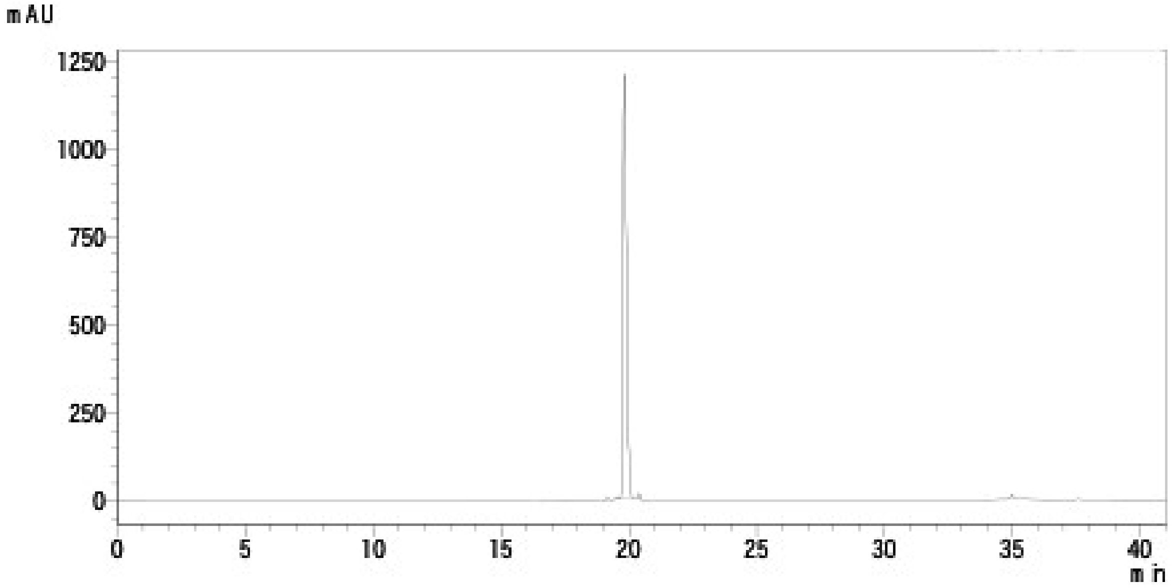

MALDI-TOF MS (**ON1**)

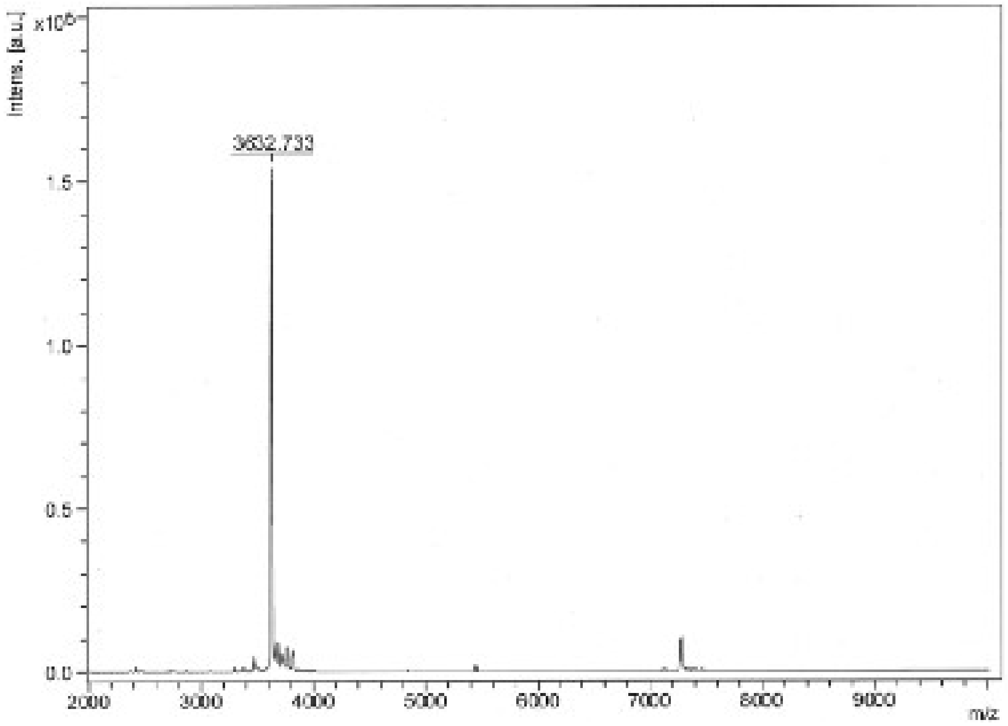

HPLC (**ON2**)

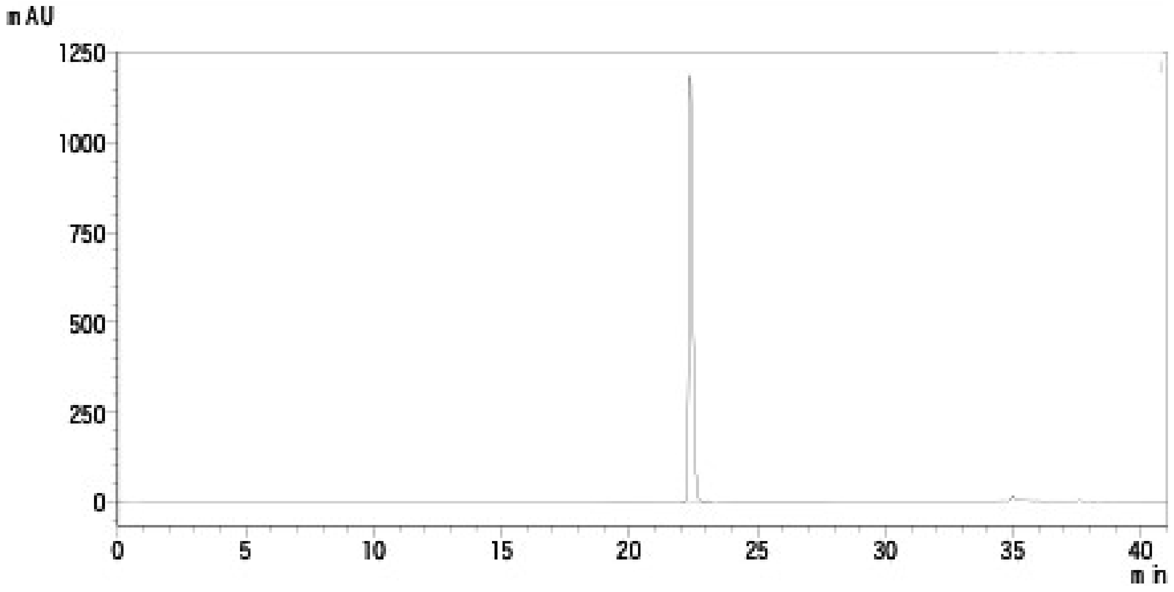

MALDI-TOF MS (**ON2**)

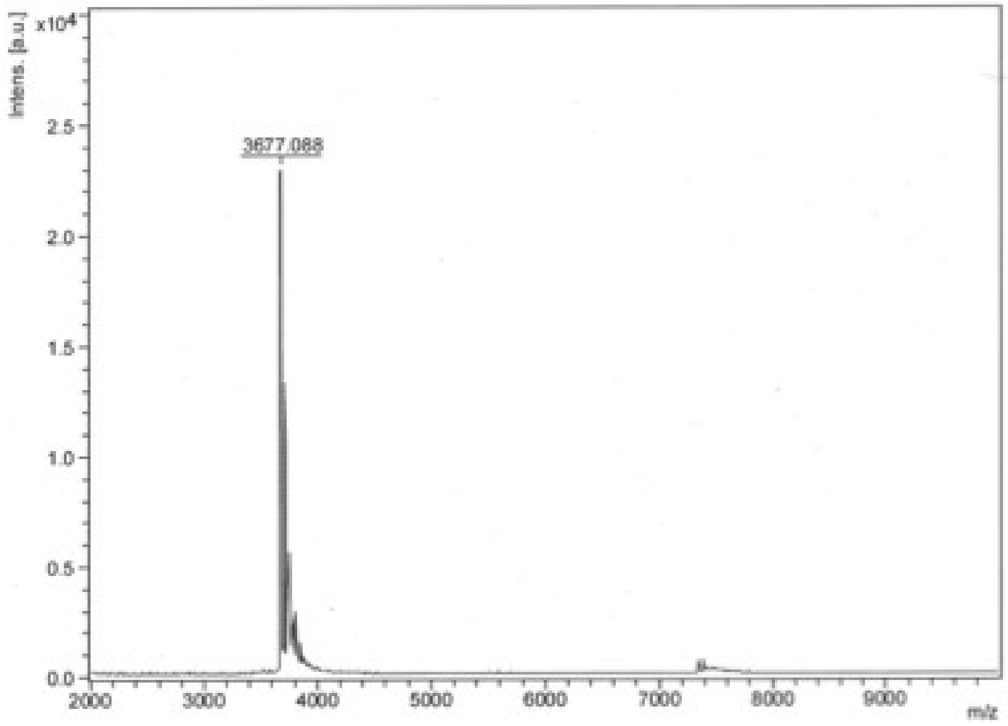

HPLC (**ON3**)

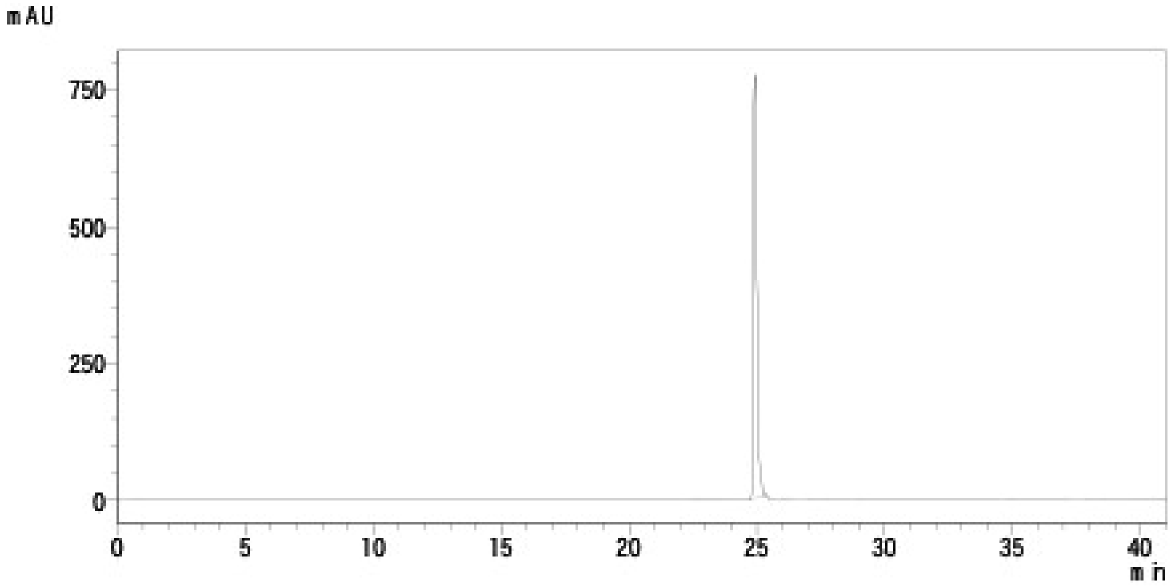

MALDI-TOF MS (**ON3**)

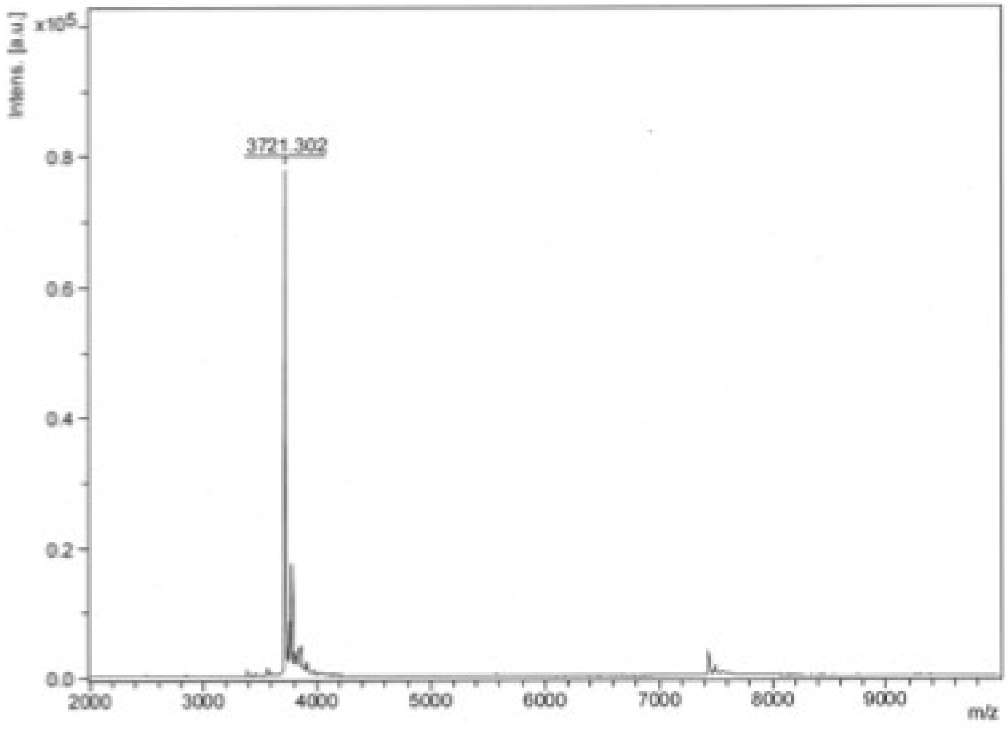

HPLC (**ON4**)

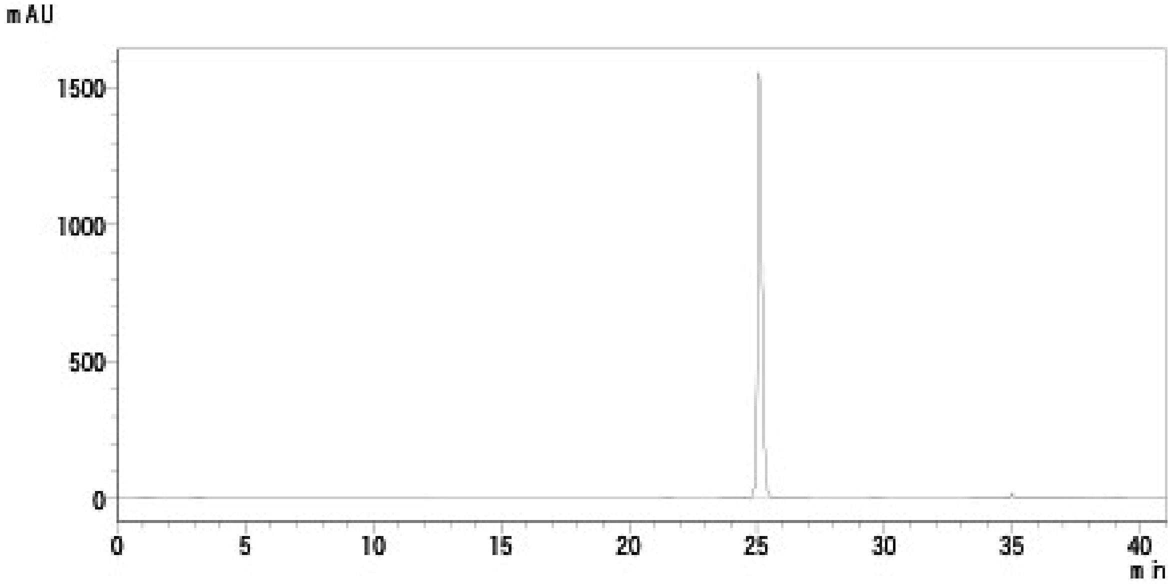

MALDI-TOF MS (**ON4**)

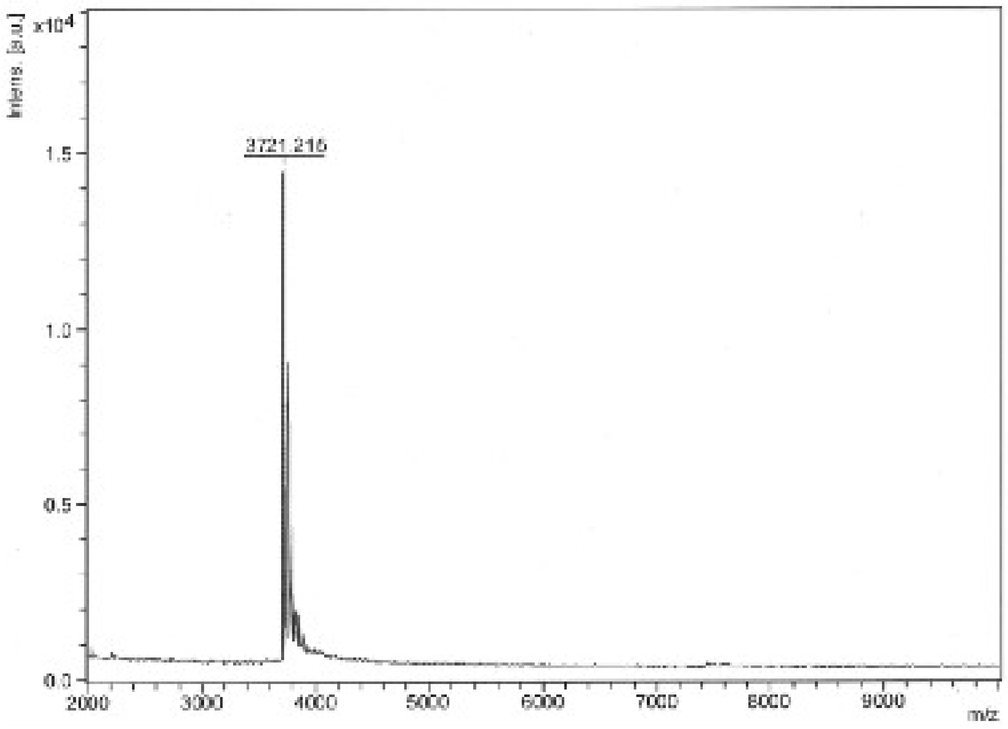

HPLC (**ON5**)

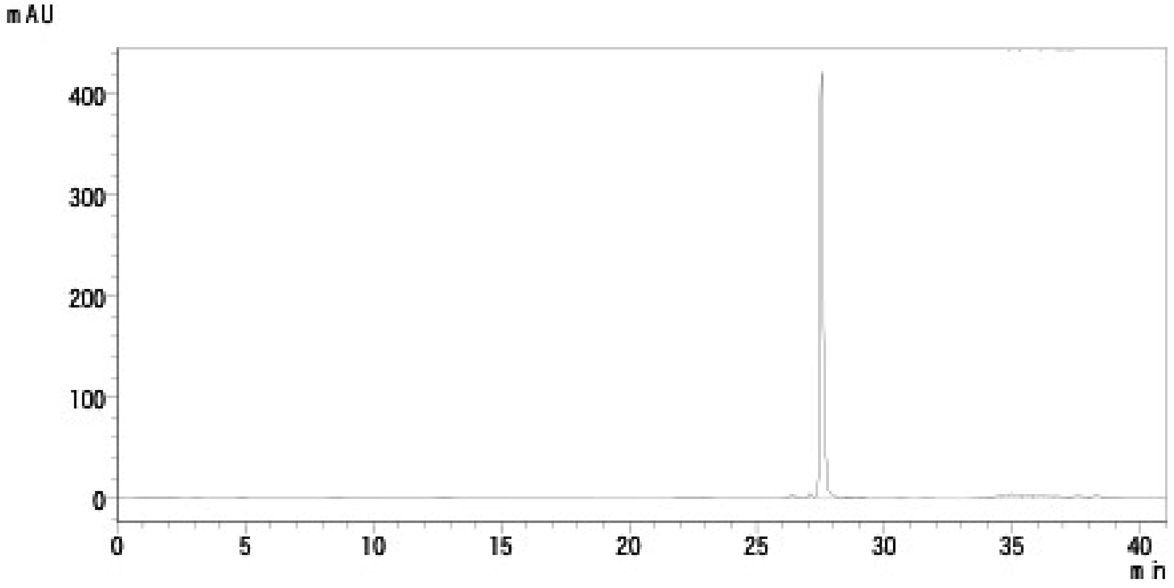

MALDI-TOF MS (**ON5**)

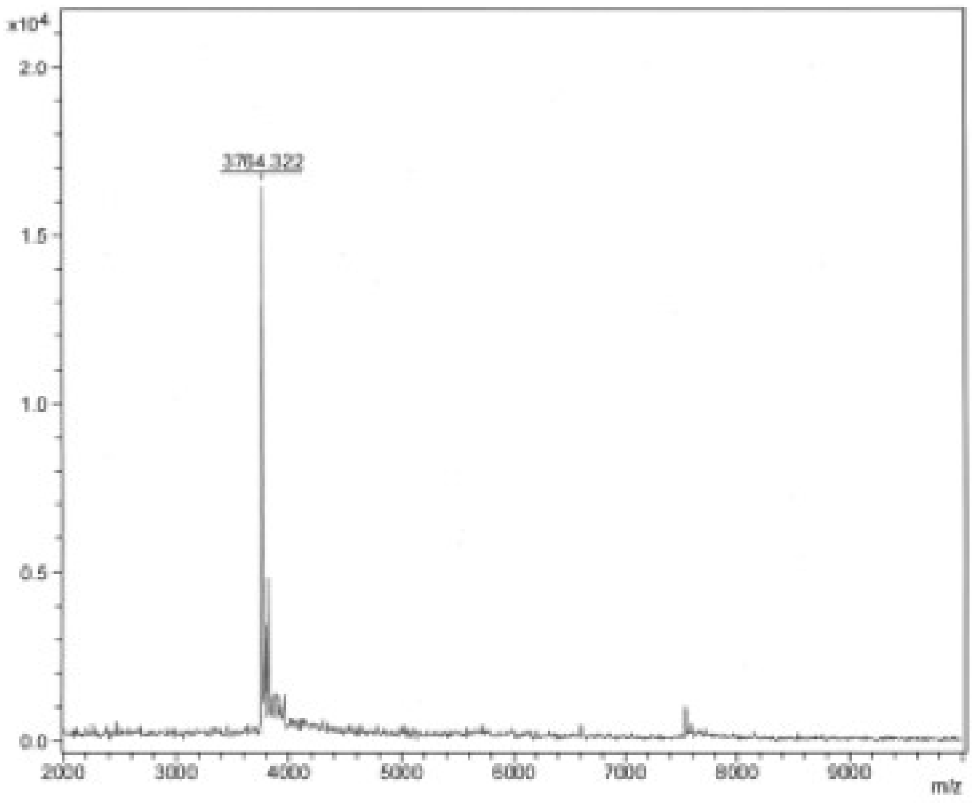

HPLC (**ON6**)

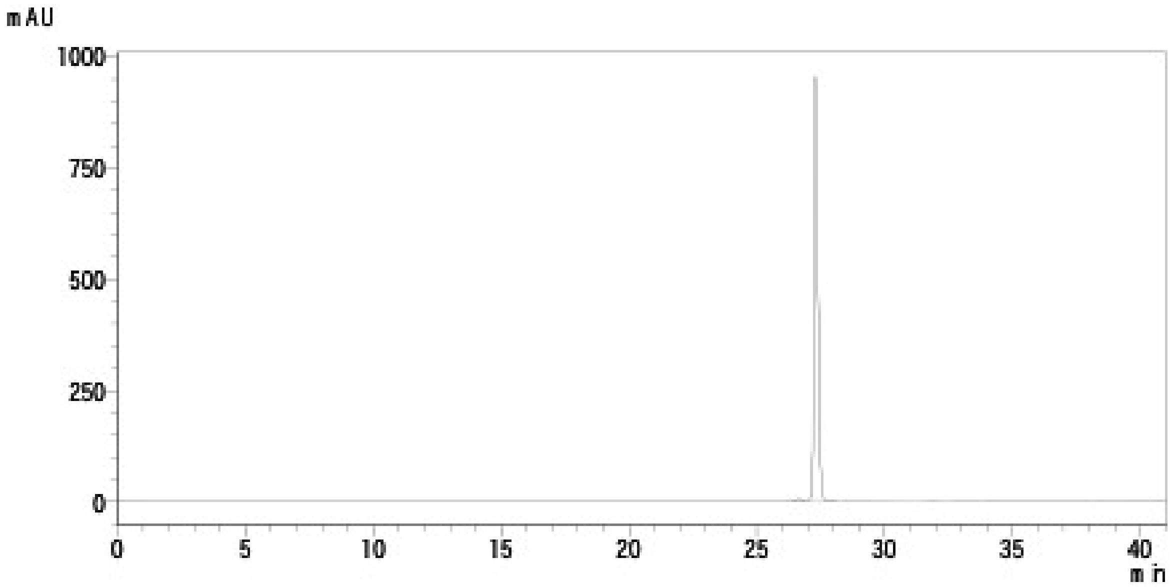

MALDI-TOF MS (**ON6**)

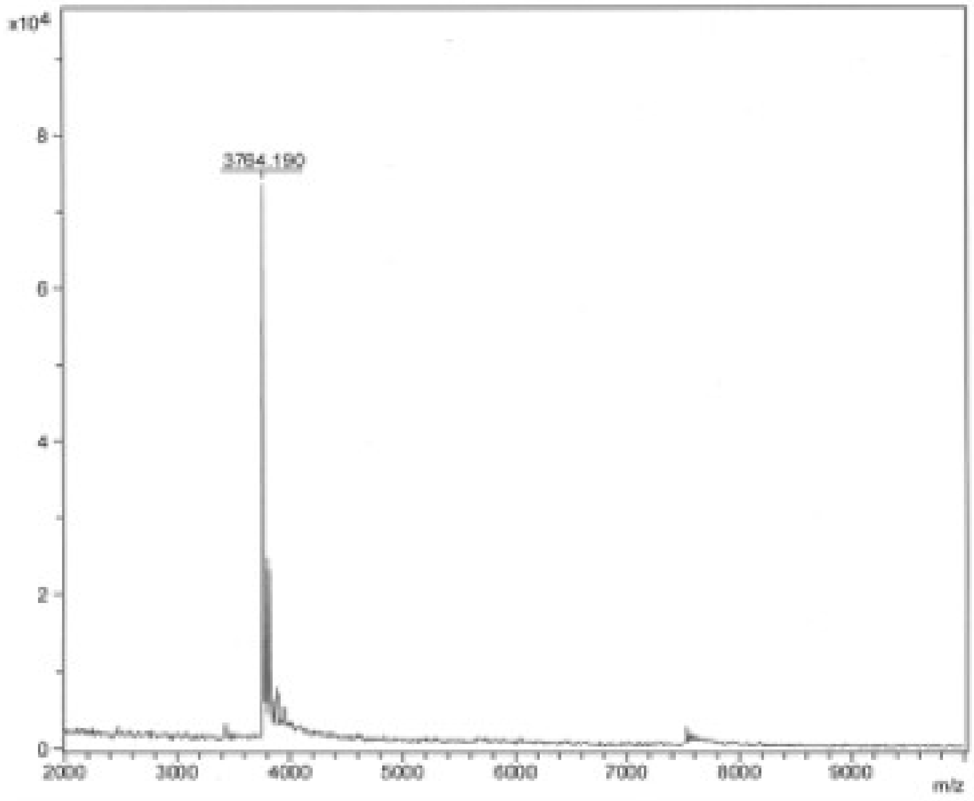

HPLC (**ON7**)

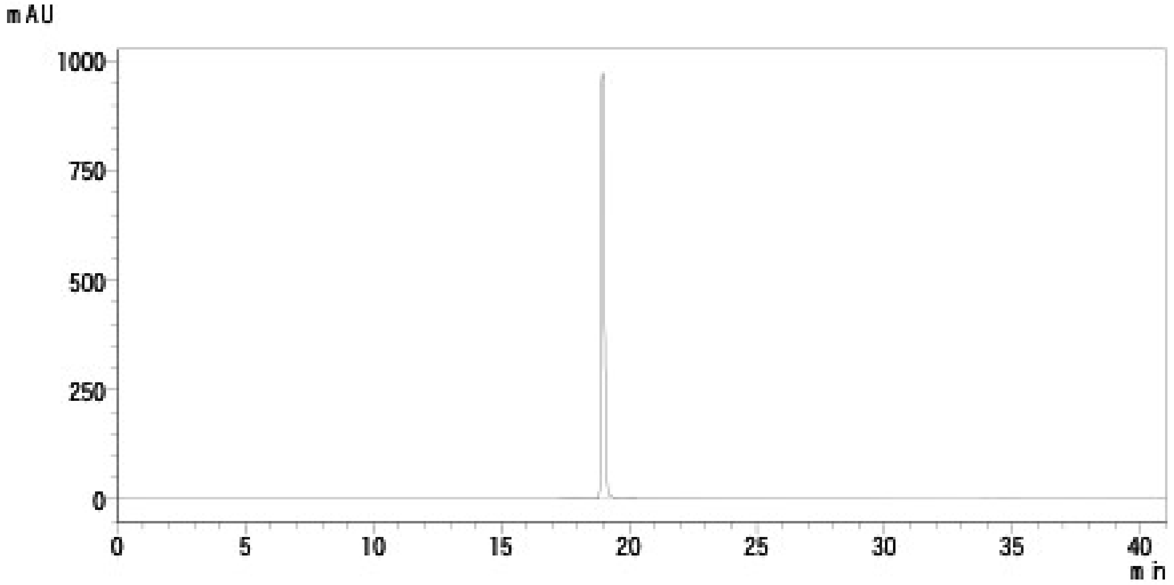

MALDI-TOF MS (**ON7**)

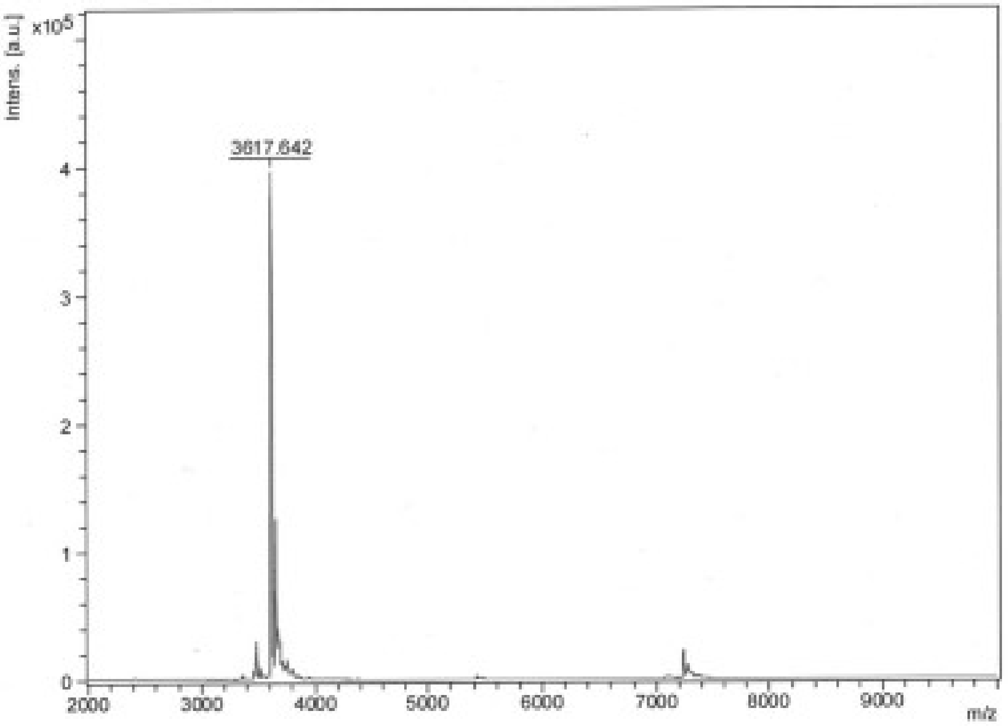

HPLC (**ON8**)

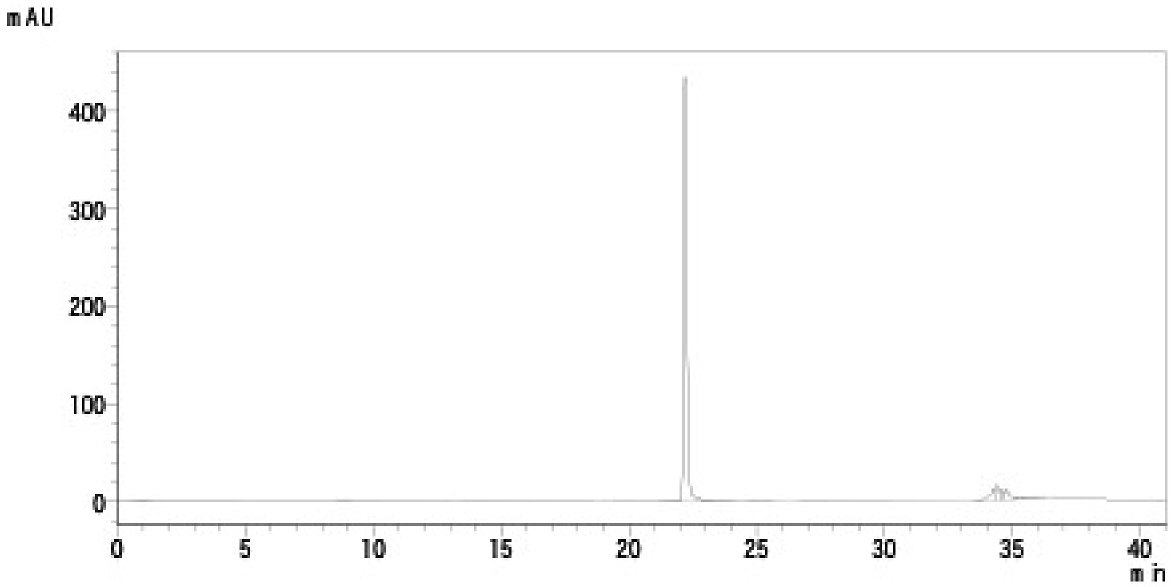

MALDI-TOF MS (**ON8**)

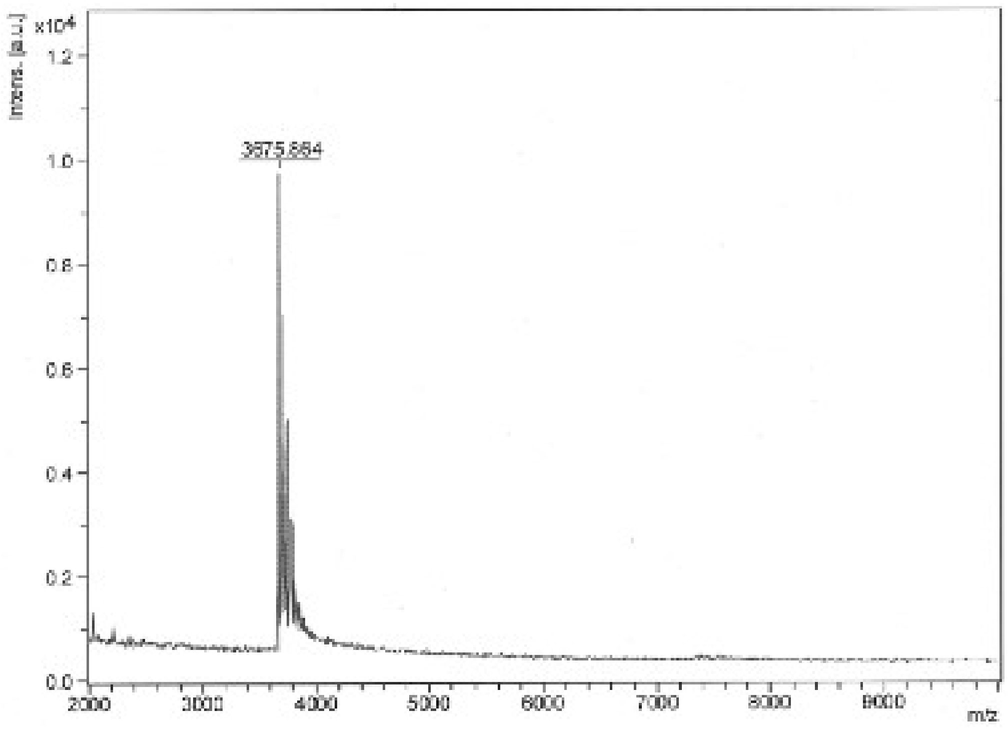

HPLC (**ON9**)

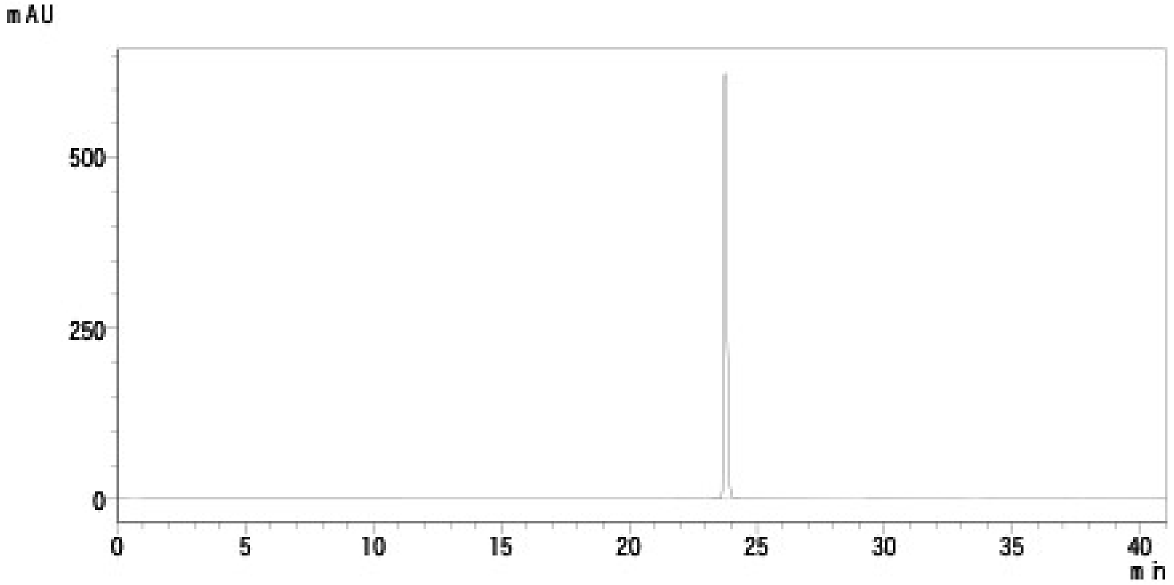

MALDI-TOF MS (**ON9**)

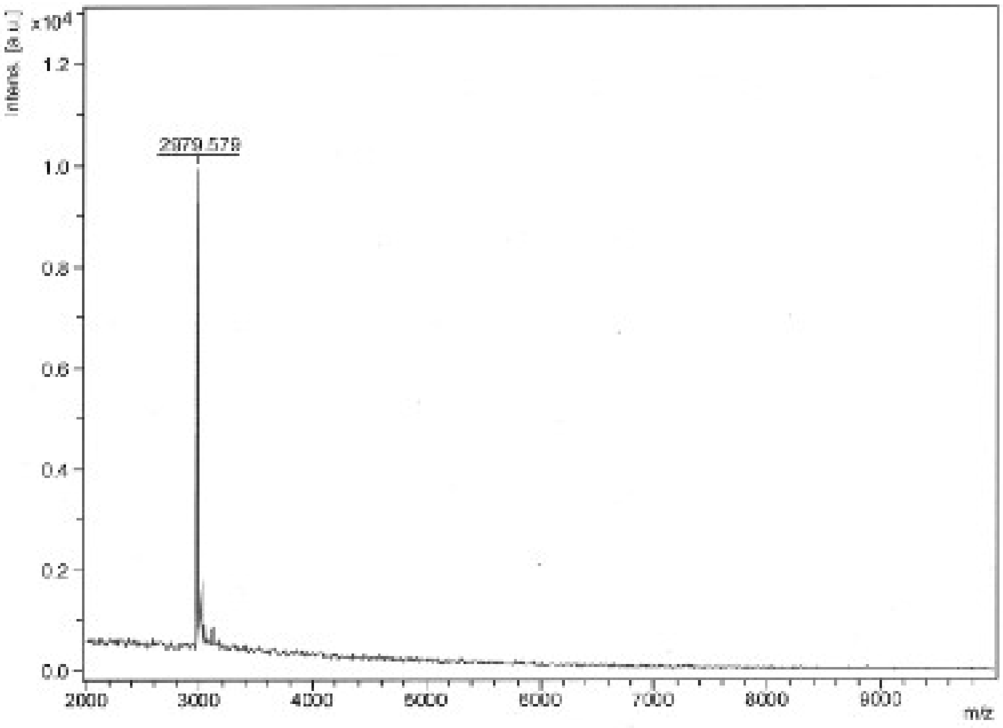

HPLC (**ON10**)

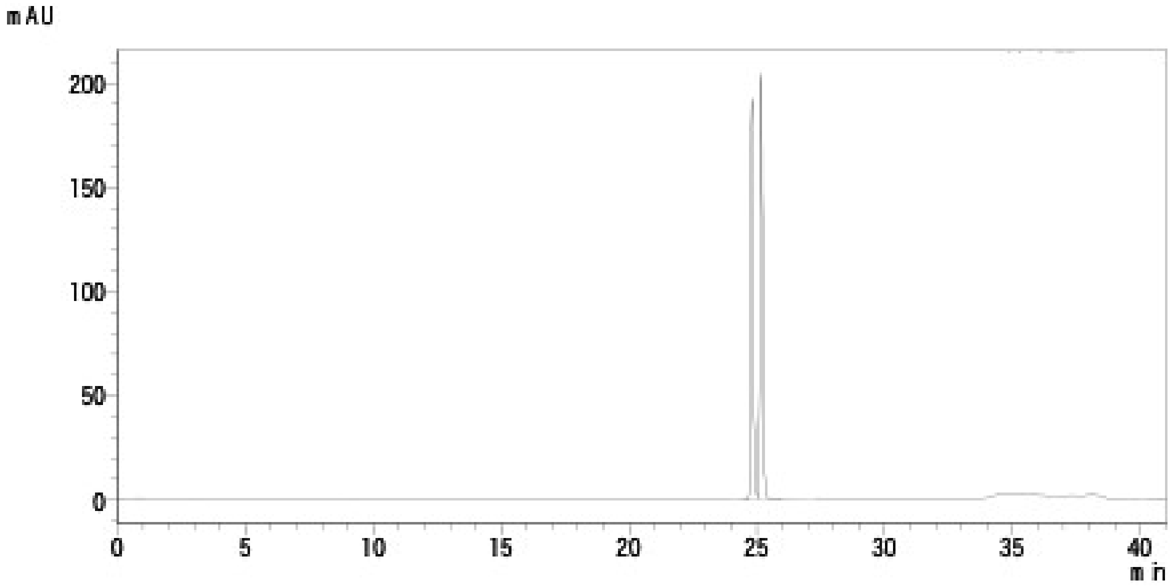

MALDI-TOF MS (**ON10**)

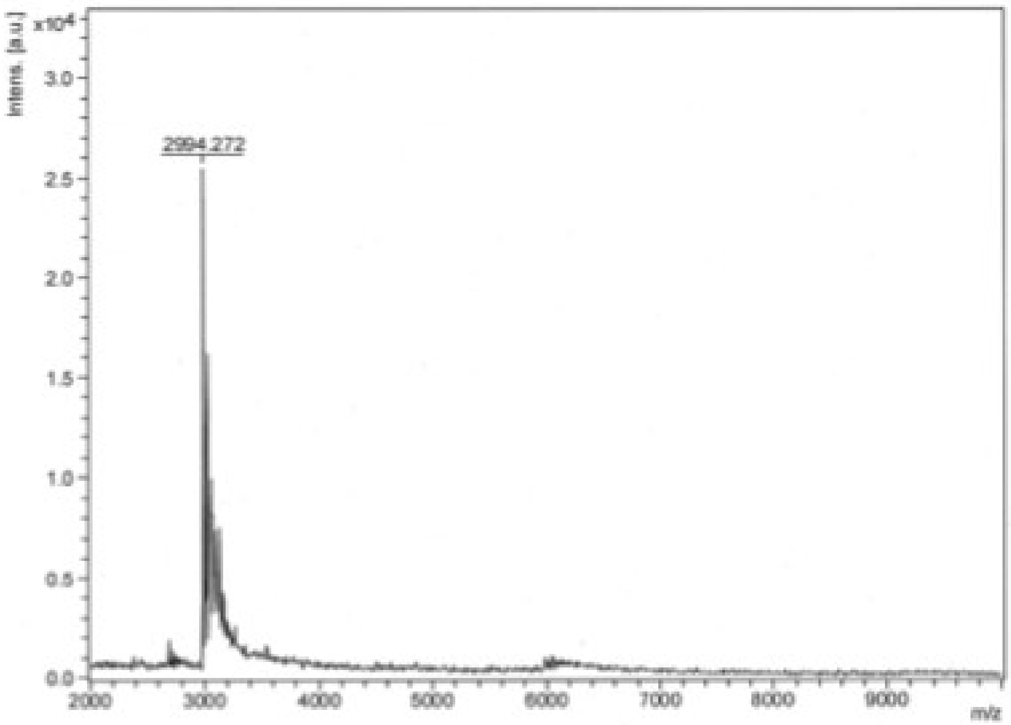

HPLC (**ON11**)

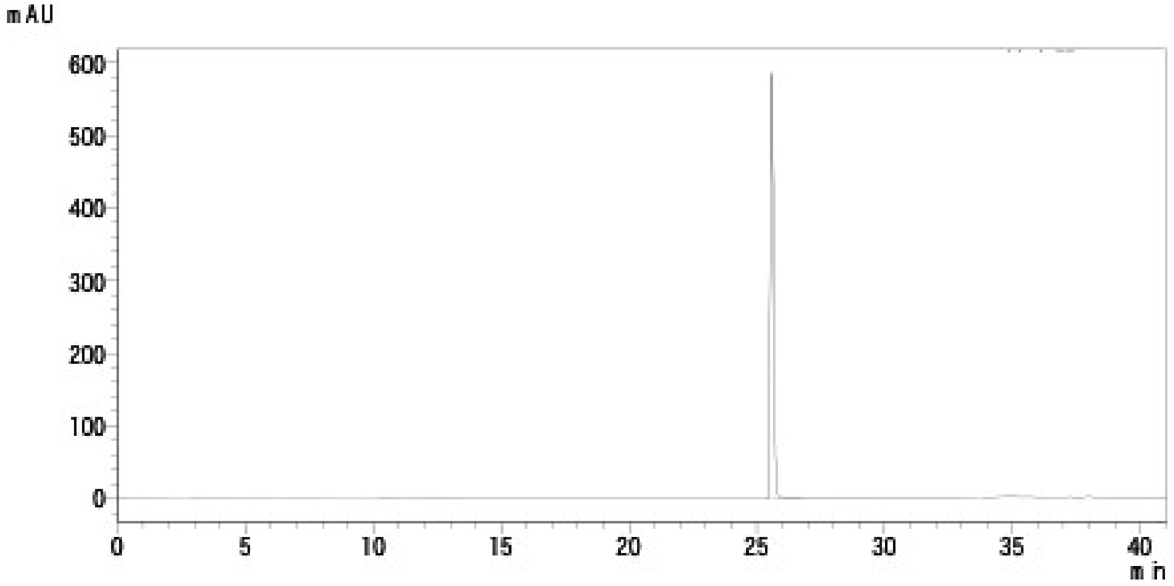

MALDI-TOF MS (**ON11**)

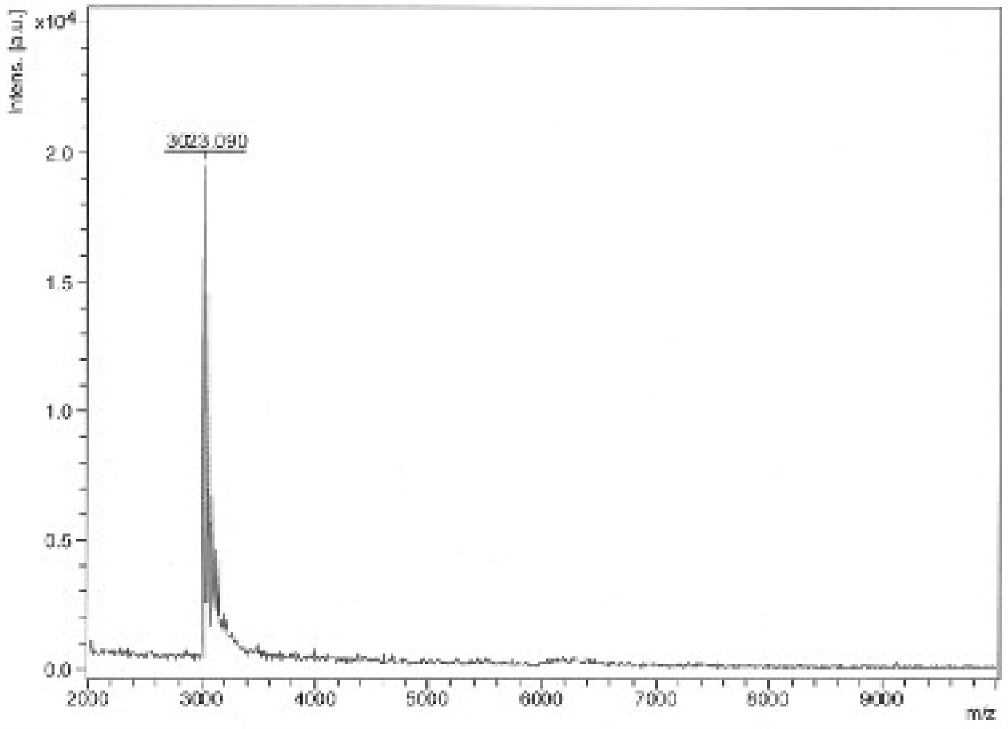

HPLC (**ON12**)

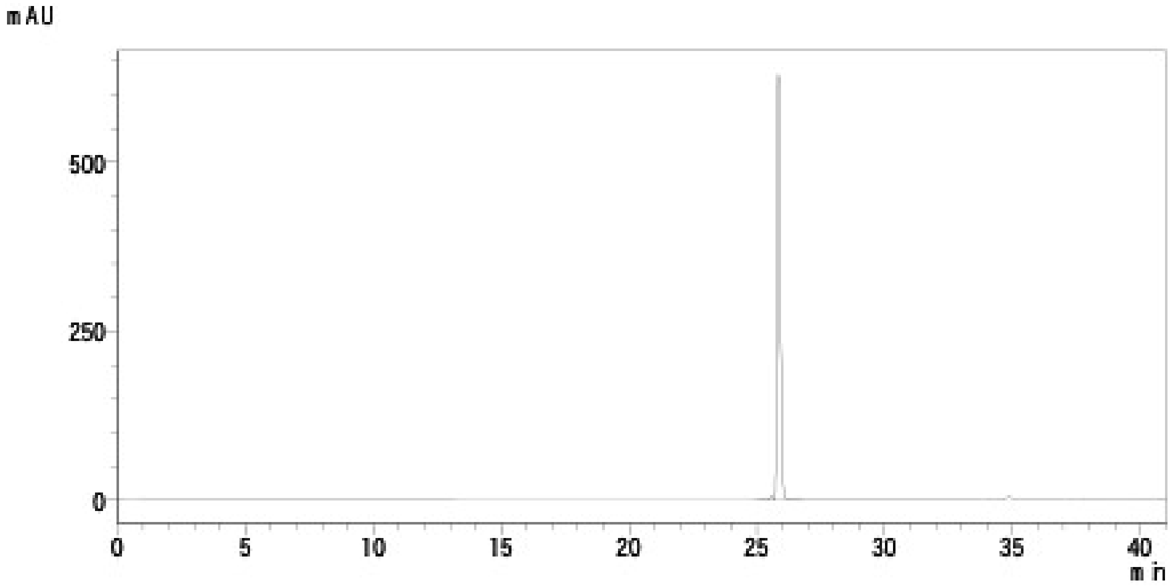

MALDI-TOF MS (**ON12**)

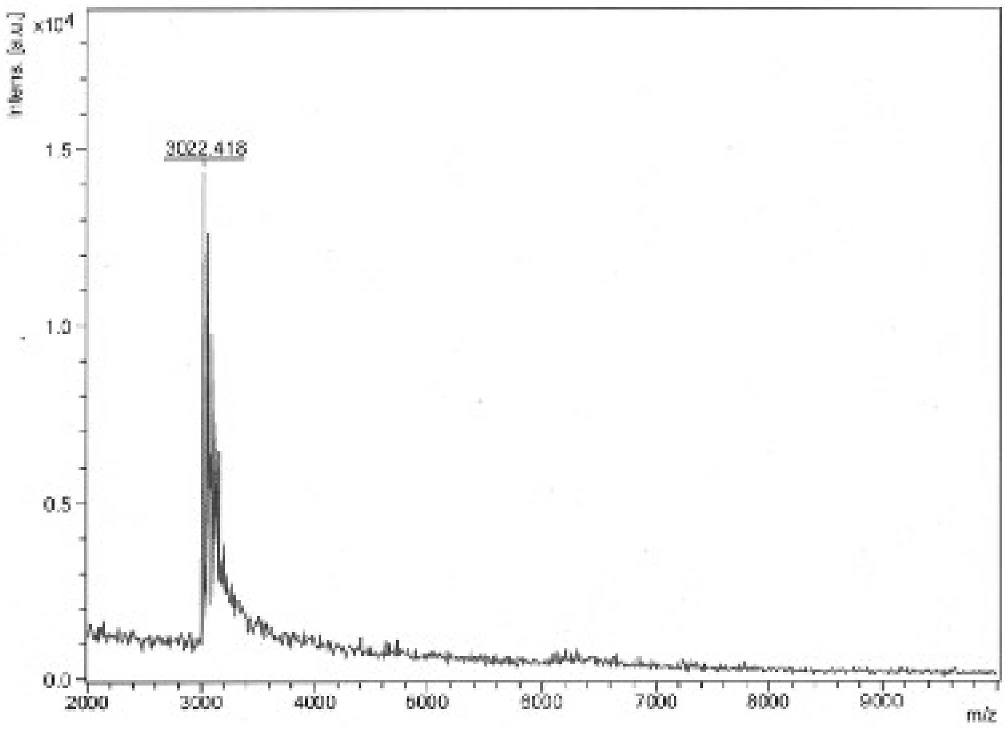

Supplement: CB-007-D6CB00034G-s001 [file CB-007-D6CB00034G-s001.pdf]
